# Integrated two-photon and photoacoustic microscopy for single-cell neurometabolic imaging

**DOI:** 10.1038/s41467-026-75603-7

**Published:** 2026-07-15

**Authors:** Jiaxiao Han, Youngseop Lee, Ziang Feng, Yue Wu, Zhuoying Wang, Allison Martinez Mejia, Adam Bauer, Manu Goyal, Jin-Moo Lee, Peinan Zhao, Hao F. Zhang, Cheng Sun, Song Hu

**Affiliations:** 1 Department of Biomedical Engineering, Washington University in St. Louis, St. Louis, MO USA; 2 Department of Biomedical Engineering, Northwestern University, Evanston, IL USA; 3 Department of Mechanical Engineering, Northwestern University, Evanston, IL USA; 4 Department of Radiology, Washington University in St. Louis, St. Louis, MO USA; 5 Department of Neurology, Washington University in St. Louis, St. Louis, MO USA; 6 Department of Obstetrics and Gynecology, Washington University in St. Louis, St. Louis, MO USA

**Keywords:** Multiphoton microscopy, Optical imaging, Neuro-vascular interactions

## Abstract

Understanding how neuronal activity couples with local energy metabolism is fundamental to brain function. Oxygen exchange between individual neurons and red blood cells (RBCs) is central to this process, yet no existing method can simultaneously capture their dynamics at single-cell resolution in vivo. Here, we introduce integrated two-photon and photoacoustic microscopy (TPM-PAM), which enables real-time imaging of single-neuron calcium activity alongside oxygen release from individual RBCs in awake mice. In TPM-PAM, a transparent micro-ring resonator-based ultrasound sensor breaks the long-standing tradeoff between optical access and acoustic sensitivity, while dual-wavelength kymography simultaneously quantifies single-RBC oxygenation and flow to derive the oxygen release rate. Incorporating nonlinear optical manipulation of the neurovascular unit with cellular precision, TPM-PAM reveals distinct neurometabolic responses to whisker stimulation, single-capillary occlusion, and single-neuron stimulation. This work establishes a powerful platform for dissecting neurometabolic coupling at the cellular scale and understanding oxygen-metabolic regulation in brain health and disease.

## Abstract (web summary)

Brain function relies on oxygen exchange between neurons and red blood cells, yet this is hard to capture at single-cell resolution in vivo. Here, the authors develop integrated two-photon and photoacoustic microscopy to image single-neuron activity and red blood cell oxygen release in awake mice.

## Introduction

Normal brain function relies on the tight coordination between neuronal activity and local energy metabolism. The brain consumes over 20% of the body’s oxygen but holds minimal reserves, relying on continuous oxygen delivery through dense capillary networks to sustain neuronal survival and signaling^1^. This process is governed by neurovascular coupling (NVC), in which neuronal activity drives local hemodynamic changes^2,3^. NVC forms the foundation of functional neuroimaging^4–6^ and plays a critical role in various neurological disorders^7^.

Wide-field optical imaging has been instrumental in neurovascular research, enabling simultaneous mapping of neuronal activity, blood oxygenation, and flow^8,9^. However, its mesoscopic resolution is inadequate to resolve individual neurons, which exhibit substantial functional heterogeneity^10^. Moreover, since oxygen delivery occurs primarily at the capillary level via individual red blood cells (RBCs)^11^, single-RBC resolution is essential for accurate quantification of local oxygen supply but is beyond the reach of wide-field methods.

Two-photon microscopy (TPM) provides subcellular resolution for imaging neuronal activity and microvascular flow^12–14^. When combined with oxygen-sensitive phosphorescent probes, TPM can also measure local oxygen partial pressure (pO_2_)^15^. However, the long lifetimes of these probes limit imaging speed and restrict measurements to sparse sites, preventing real-time mapping of oxygen dynamics. As a result, TPM offers only a spatiotemporally limited view of neurometabolic coupling (NMC)—the metabolic processes underlying NVC.

Photoacoustic microscopy (PAM) complements TPM by exploiting blood absorption for label-free, high-resolution, high-speed imaging of hemoglobin concentration (C_Hb_), blood flow (V_f_), and oxygen saturation (sO_2_)^16,17^. Despite its broad applications in functional microvascular imaging, PAM remains poorly suited for neuronal recording. Although genetically encoded calcium indicator-based photoacoustic tomography has detected neural activity at the regional level^18^, the signal is too weak to scale down for single-neuron measurements in vivo.

Efforts to integrate PAM with TPM or confocal microscopy for neurovascular imaging have shown promise but face intrinsic optical-acoustic tradeoff. Conventional PAM uses opaque piezoelectric transducers, which block the light path and complicate its integration with TPM. Existing strategies, such as optical-acoustic combiners reflecting ultrasound to side-mounted transducers or ring-shaped transducers with central openings for light transmission^19^, require the use of long-working-distance microscope objectives with low numerical apertures (NAs), compromising both TPM resolution and fluorescence collection efficiency. Miniaturized piezoelectric transducers positioned beneath the objective or at an oblique angle improve optical access but reduce acoustic sensing area and, in the latter case, disrupt optical-acoustic confocal alignment—thereby limiting ultrasound sensitivity^20–22^. Transparent piezoelectric transducers^23,24^ and optical-based acoustic sensors, such as Fabry-Perot^25^ and fiber laser sensors^26^, facilitate the integration of PAM with other optical modalities. Yet, their limited detection bandwidth hinders functional imaging of single RBCs, which generate broadband, high-frequency acoustic signals. Photoacoustic remote sensing microscopy (PARS) enables non-contact imaging for straightforward multimodal integration^27,28^. However, accurate derivation of blood oxygenation from the measured reflectivity modulation warrants further investigation, as potential nonlinear effects involved in the PARS signal generation should be carefully accounted for^29^. Consequently, current multimodal systems cannot achieve functional PAM at single-RBC level. Moreover, they lack the ability to manipulate the neurovascular unit with cellular precision, restricting studies to passive observation or global perturbation^21^.

To overcome these limitations, we developed an integrated two-photon and photoacoustic microscopy (TPM-PAM) platform. By exploiting an optically transparent micro-ring resonator (MRR) as an ultrasound sensor^30^, TPM-PAM achieves high-sensitivity, broadband acoustic detection while preserving full optical access. This enables simultaneous imaging of calcium activity of individual neurons and oxygen-metabolic dynamics of single RBCs, including C_Hb_, V_f_, sO_2_, and oxygen release rate (rO_2_). Moreover, TPM-PAM incorporates nonlinear optical manipulation, allowing targeted neurovascular perturbation with micron-level precision. In awake mice, TPM-PAM revealed distinct neurometabolic responses to physiological and targeted perturbations, including whisker stimulation, single-capillary occlusion, and single-neuron stimulation, all at single-cell resolution.

## Results

### Design of TPM-PAM

Aiming for all-optical neurometabolic imaging and manipulation at single-cell resolution, we designed a TPM-PAM system that addressed three major technical challenges.

To overcome the optical-acoustic tradeoff in the integration of TPM and PAM, we developed a transparent polymer MRR-based ultrasound sensor. The polymer MRR, with a matching bus waveguide, was fabricated on a spin-on-glass (SOG)-coated glass substrate using soft nanoimprint lithography (Supplementary Fig. 1). We employed comprehensive optical-mode engineering by incorporating adiabatically tapered waveguide structures to minimize mode mismatch with the single-mode fiber input and by optimizing the cladding and substrate materials to reduce optical propagation loss within the micro-ring waveguide while improving acoustic impedance matching at the water-waveguide interface (Supplementary Note 1). These design innovations yielded a four-fold improvement in the coupling efficiency (i.e., the optical power transmission efficiency from the input fiber to the output fiber through the MRR), and a five-fold increase in its quality factor (Q-factor) (Supplementary Note 1), collectively leading to more than one order of magnitude enhancement in ultrasound detection sensitivity compared with the previous design^30^. The fully packaged MRR incorporates a single-mode fiber input and a multi-mode fiber output for reliable integration with the interrogation system. It is optically transparent and has a total thickness of 370 μm, including the carrier glass substrate. This compact formfactor allows the MRR to be directly positioned beneath a high-NA (1.0) objective with a short working distance (2 mm). The MRR enables broadband ultrasound detection up to 100 MHz with high sensitivity, without introducing measurable optical aberrations to TPM imaging in vivo (Supplementary Fig. 2).

To extend PAM-based oxygen-metabolic measurements from tissue scale^17^ to single-RBC level, we combined spectroscopic and kymographic PAM measurements to simultaneously quantify the sO_2_ and V_f_ of individual RBCs. This enabled direct estimation of the amount of oxygen released from single RBCs to surrounding tissue per unit time (i.e., rO_2_). Specifically, kymographic scanning was performed along multiple capillaries within the field of view (FOV) by following a user-defined trajectory, with the excitation wavelength alternated between two wavelengths on a pulse-by-pulse basis. In the obtained sO_2_-encoded kymograph, individual RBCs or RBC clusters were segmented, and the oxygen release of each cluster was derived as the product of the sO_2_ gradient along the flow direction, the flow speed, and the number of RBCs within the cluster (Supplementary Fig. 3). Summation of the rO_2_ values across all RBC clusters traversing a given capillary yielded the vessel’s total rO_2_. Applying this method across all capillaries and computationally diffusing the vascular rO_2_ to surrounding tissue enabled spatial mapping of oxygen delivery within the FOV (see the Methods for details).

Dissecting neurometabolic coupling requires the ability to not only image but also perturb the neurovascular unit with cellular precision. Conventional approaches, such as electrical stimulation or optogenetics, have major limitations when combined with TPM-PAM. Electrical stimulation lacks spatial specificity and typically requires anesthesia, whereas optogenetics introduces spectral overlaps with PAM excitation and creates crosstalk. To circumvent these issues, TPM-PAM used femtosecond (fs) laser pulses confocally aligned with the imaging beams to induce targeted single-capillary occlusion^31^ or single-neuron stimulation^32,33^ in the awake mouse brain. This nonlinear optical manipulation offered micron-scale spatial precision and avoided the use of exogenous agents.

**Fig. 1:**
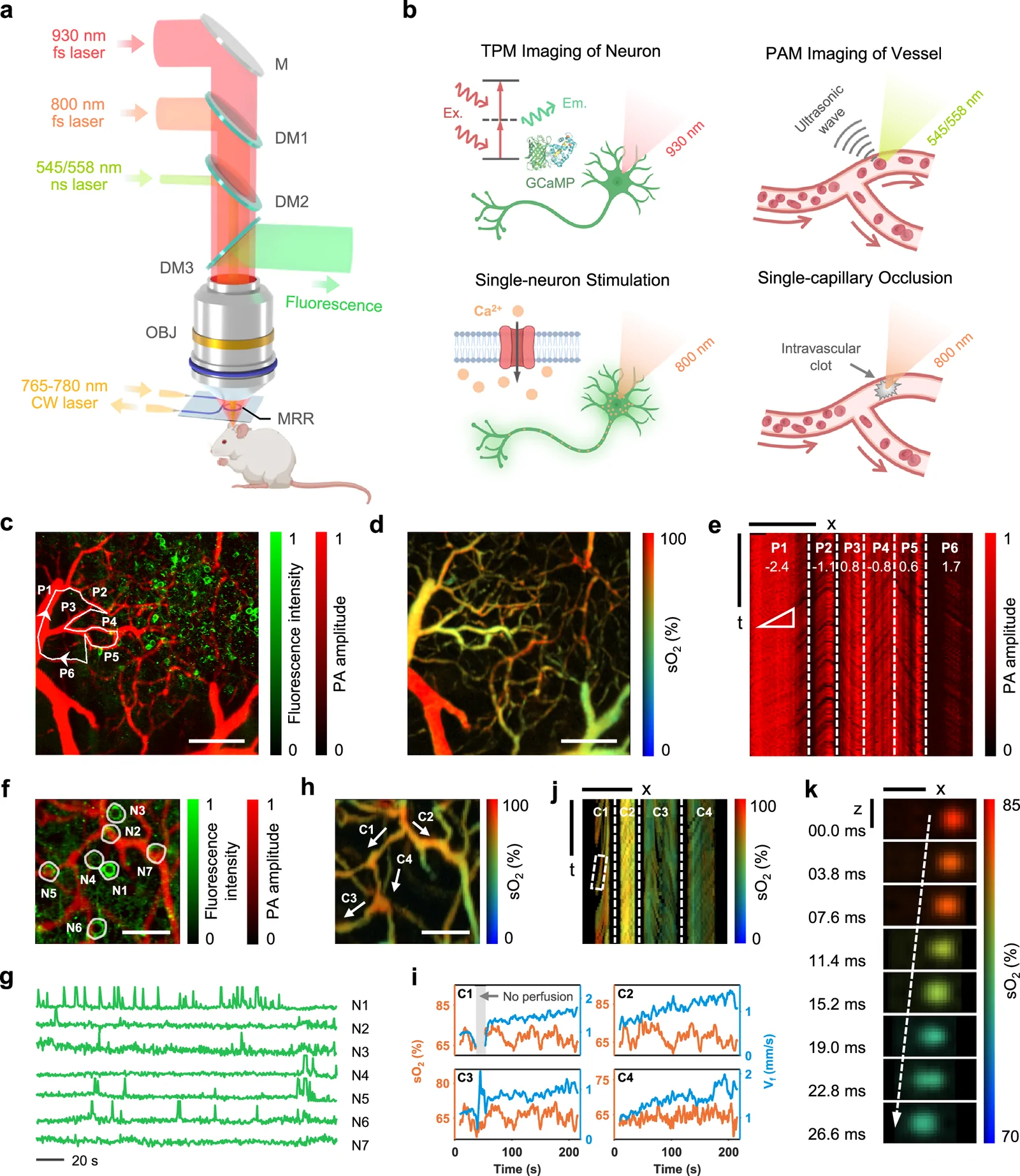
Schematic of TPM-PAM and in vivo performance in the awake GCaMP mouse brain. **a** Simplified schematic of the TPM-PAM system. M, mirror; DM, dichroic mirror; OBJ, objective; MRR, micro-ring resonator. **b** Diagram illustrating the capabilities of TPM-PAM, including dual-modal imaging (top) and nonlinear optical manipulation (bottom) at the single-neuron (left) and single-capillary (right) level. **c** Fused TPM-PAM image of neuronal calcium activity (fluorescence) and vascular hemoglobin concentration (C_Hb_), which is derived from the photoacoustic (PA) amplitude acquired at the isosbestic wavelength of 545 nm. **d** Corresponding map of hemoglobin oxygen saturation (sO_2_). **e** PAM-based kymograph acquired along the trajectory indicated by the white line in (**c**). Numbers are the measured flow speeds in each vessel segment separated by dotted lines. Positive values indicate flow in the scanning direction, while negative values indicate flow in the opposite direction. **f** Fused TPM-PAM image over a reduced field of view. **g** Calcium activity traces of seven neurons circled in (**f**). The fluorescence intensity in each trace is normalized to its maximum value. **h** sO_2_ map corresponding to (**f**) with arrows indicating flow directions. **i** Temporal dynamics of sO_2_ and V_f_ in four capillaries marked in (**h**). **j** sO_2_-encoded dual-wavelength kymograph. **k** Zoom-in view of the white-dashed box in (**j**) showing oxygen release from a single RBC during its transit through capillary C1. Scale bars: **c, d** 100 µm; **e** x = 200 µm, t = 200 ms; **f**, **h** 50 µm; **j** x = 100 µm, t = 50 ms; **k** x = 10 µm, z = 30 µm. Panels **a** and **b** contain elements created in BioRender. Han, J. (https://BioRender.com/wa5vvjv).

Integrating these design components, we developed a TPM-PAM platform capable of single-cell neurometabolic imaging and manipulation (Fig. 1a and Supplementary Fig. 4). A detailed description of this system can be found in the Methods. Briefly, TPM-PAM used four laser lines: 930-nm fs pulses for TPM of single-neuron calcium activity; dual 545/558-nm nanosecond (ns) pulses for PAM of single-RBC oxygen dynamics; 800-nm fs pulses for targeted single-capillary or single-neuron manipulation (Fig. 1b); and a wavelength-tunable (765–780 nm), continuous-wave (CW) laser beam for MRR interrogation. The system achieved lateral/axial resolutions of 0.7/2.4 μm for TPM and 2.5/15 μm for PAM (Supplementary Fig. 5) and enabled simultaneous imaging of single-neuron calcium activity, alongside single-RBC C_Hb_, sO_2_, V_f_, and rO_2_, over a 200 × 200 μm^2^ FOV at ~3 Hz.

### Single-cell neurometabolic imaging in awake mice

We first performed TPM-PAM imaging over a relatively large FOV of 400 × 400 μm^2^ with a 1-μm step size in an awake, head-fixed GCaMP mouse through an open-skull cranial window. Raster scanning simultaneously mapped neuronal activity (fluorescence intensity in Fig. 1c), C_Hb_ (PA amplitude in Fig. 1c), and sO_2_ (Fig. 1d). Kymographic scanning was then performed along a user-defined trajectory (white line with arrows in Fig. 1c) to measure V_f_ (Fig. 1e), which was quantified for each vessel using the Radon transform (Supplementary Fig. 6). These in vivo measurements demonstrate that TPM-PAM provides high resolution and sensitivity for multi-parametric characterization of the neurovascular unit at single-cell level.

We next reduced the FOV to 150 × 150 μm^2^ to demonstrate the capability of TPM-PAM for dynamic neurometabolic recording (Supplementary Movie 1). In this configuration, the frame rate was increased to 3 Hz, capturing spontaneous calcium activity in seven neurons (circled in Fig. 1f, with activity traces shown in Fig. 1g) and concurrent sO_2_ and V_f_ dynamics in four capillaries (labeled in Fig. 1h, with time courses shown in Fig. 1i). To quantify oxygen release from individual RBCs, we applied dual-wavelength, ns-pulse excitation during kymographic scanning to track sO_2_ variations of single RBCs or RBC clusters along their flow trajectories (Fig. 1j). The zoom-in view of the boxed region in Fig. 1j reveals a gradual sO_2_ decline as a single RBC traverses a capillary, directly visualizing the oxygen release process (Fig. 1k).

### Neurometabolic responses to whisker stimulation

**Fig. 2:**
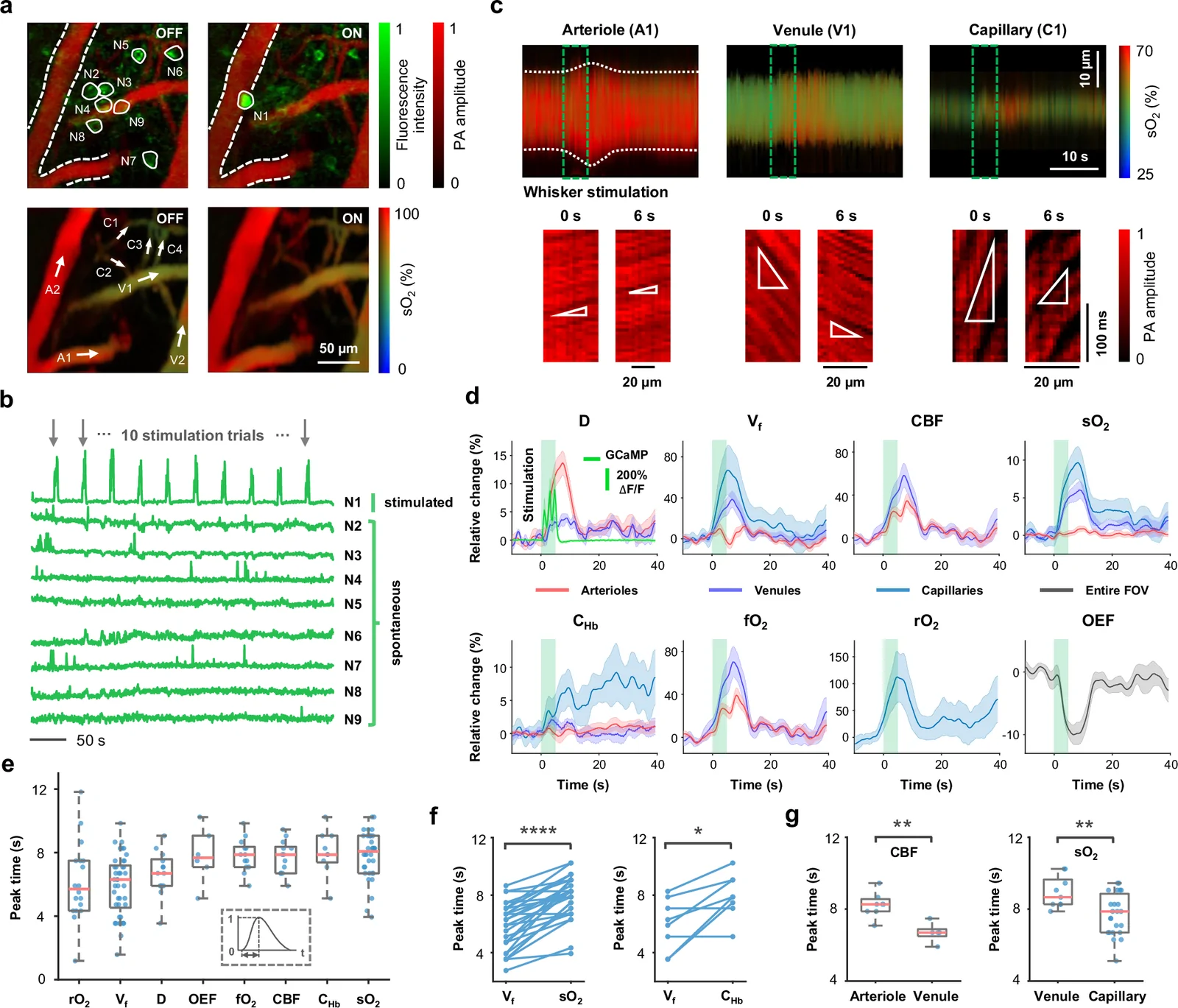
Neurometabolic responses to whisker stimulation in the awake GCaMP mouse brain. **a** Representative TPM-PAM images acquired before (left) and after (right) a single stimulation trial. Top: overlay of neuronal activity and vascular C_Hb_. Dotted outline marks the boundary of a dilated arteriole after stimulation. Bottom: sO_2_ map, with arrows indicating flow directions. **b** Calcium activity traces of nine neurons circled in (**a**). The fluorescence intensity in each trace is normalized to its maximum value. Arrows denote stimulation onset. **c** Temporal dynamics of sO_2_-encoded vessel cross-section during stimulation (top), and corresponding flow kymographs at 0 s and 6 s after stimulation onset (bottom) in arteriole A1 (left), venule V1 (middle) and capillary C1 (right). **d** Relative changes in eight oxygen-metabolic parameters averaged across ten trials for arterioles (red), venules (purple), capillaries (blue) and the entire FOV (gray). Green shadows indicate the stimulation period. **e** Peak times of the eight parameters measured across all vessels over the ten trials, ranked in an ascending order based on their median values. **f** Paired *t*-tests comparing V_f_ with sO_2_ (left) and C_Hb_ (right) (**** *p* < 0.0001, * 0.01 ≤ *p* < 0.05). **g** Unpaired *t*-tests comparing CBF between arterioles and venules (left) and sO_2_ between venules and capillaries (right) (** 0.001 ≤ *p* < 0.01).

To validate the applicability of TPM-PAM for studying NMC under physiological conditions, we performed whisker stimulation in an awake, head-fixed GCaMP mouse. A 5-s stimulation trial was repeated 10 times with a 40-s inter-trial interval, and neurometabolic responses were recorded throughout the process (Supplementary Movie 2). Representative TPM-PAM images acquired before and after stimulation are shown in Fig. 2a, with a total of nine neurons (N1–N9) and eight vessels (two arterioles A1–A2, two venules V1–V2, and four capillaries C1–C4) resolved within a FOV of 200 × 200 μm^2^. Among these neurons, N1 showed a robust and consistent response across trials, whereas others exhibited activities that were not synchronized with the stimulation pattern, indicating spontaneous activity (Fig. 2b). Microvascular responses varied by vessel types, with arterioles presenting marked vasodilation while venules and capillaries showing elevated sO_2_ and V_f_ (Fig. 2c).

We performed quantitative analysis on eight oxygen-metabolic parameters: diameter (D), V_f_, volumetric flow (CBF), sO_2_, C_Hb_, oxygen flux rate (fO_2_, amount of oxygen flowing through the vessel per unit time), and rO_2_ for individual vessels, as well as oxygen extraction fraction (OEF) over the entire FOV. Detailed definitions of these parameters are provided in the Methods. Figure 2d summarizes the mean responses of each vessel type across the trials, while the data acquired from individual vessels are presented in Supplementary Fig. 7. Because RBC passage in capillaries is intermittent, the diameter—as well as CBF and fO_2_, which depend on D—were not assessed for capillaries. Conversely, rO_2_ was quantified only for capillaries, where oxygen release primarily occurs.

Distinct vessel-type-specific responses were observed. In arterioles, V_f_ remained largely unchanged, whereas D increased by 14% ± 2% (mean ± standard deviation)—resulting in a 35% ± 7% increase in CBF. Given the minimal changes in sO_2_ and C_Hb_, the elevated CBF directly translated into a 39% ± 7% rise in fO_2_, indicating that vasodilation is the dominant mechanism to enhance arteriolar oxygen supply. In venules, CBF increased by 58% ± 11%, driven mainly by a marked rise in V_f_ (38% ± 8%) and a modest dilation in D (4% ± 1%). Combined with a 6% ± 1% increase in sO_2_, these changes yielded a 70% ± 14% rise in fO_2_, reflecting elevated oxygen drainage because of surplus oxygen delivery from upstream arterioles. Regionally, OEF decreased by 10% ± 2%, suggesting that the oxygen supply exceeded the immediate metabolic demand. In capillaries, V_f_ increased by 67% ± 24%, C_Hb_ by 9% ± 4%, and sO_2_ by 10% ± 2%, all consistent with elevated blood and oxygen inflow from arterioles. Capillary rO_2_ surged by 113% ± 48%, directly indicating enhanced local oxygen release from RBCs to support the evoked neuronal activation.

We next analyzed the temporal dynamics of these oxygen-metabolic responses. For each parameter, the peak time, defined as the interval from stimulus onset to maximum amplitude of the response, was extracted across trials and ranked in an ascending order by the median value (Fig. 2e). Pairwise comparisons using paired *t*-tests (Supplementary Fig. 8) revealed that V_f_ responses occurred significantly earlier than those of both sO_2_ and C_Hb_ (Fig. 2f). Between vessel types, unpaired *t*-tests revealed that venular CBF peaked earlier than arteriolar CBF, while capillary sO_2_ responses preceded those in venules (Fig. 2g). Additional analyses of the rise time and decay time are presented in Supplementary Figs. 9 and 10.

### Neurometabolic responses to single-capillary occlusion

To demonstrate the capability of TPM-PAM for microvascular manipulation and concurrent neurometabolic recording at the cellular scale, we induced targeted occlusion of a single capillary in an awake, head-fixed GCaMP mouse using high-energy ultrashort pulses delivered by the 800-nm fs laser line. This approach achieved localized vessel occlusion without exogenous agents, while enabling simultaneous monitoring of neurometabolic responses throughout the process (Supplementary Movie 3). The occlusion was achieved by focusing the fs-laser beam on the target capillary for 1–5 s, followed by imaging to assess the occlusion status. This alternating procedure was repeated with progressively increased laser exposure until complete blockage was confirmed.

**Fig. 3:**
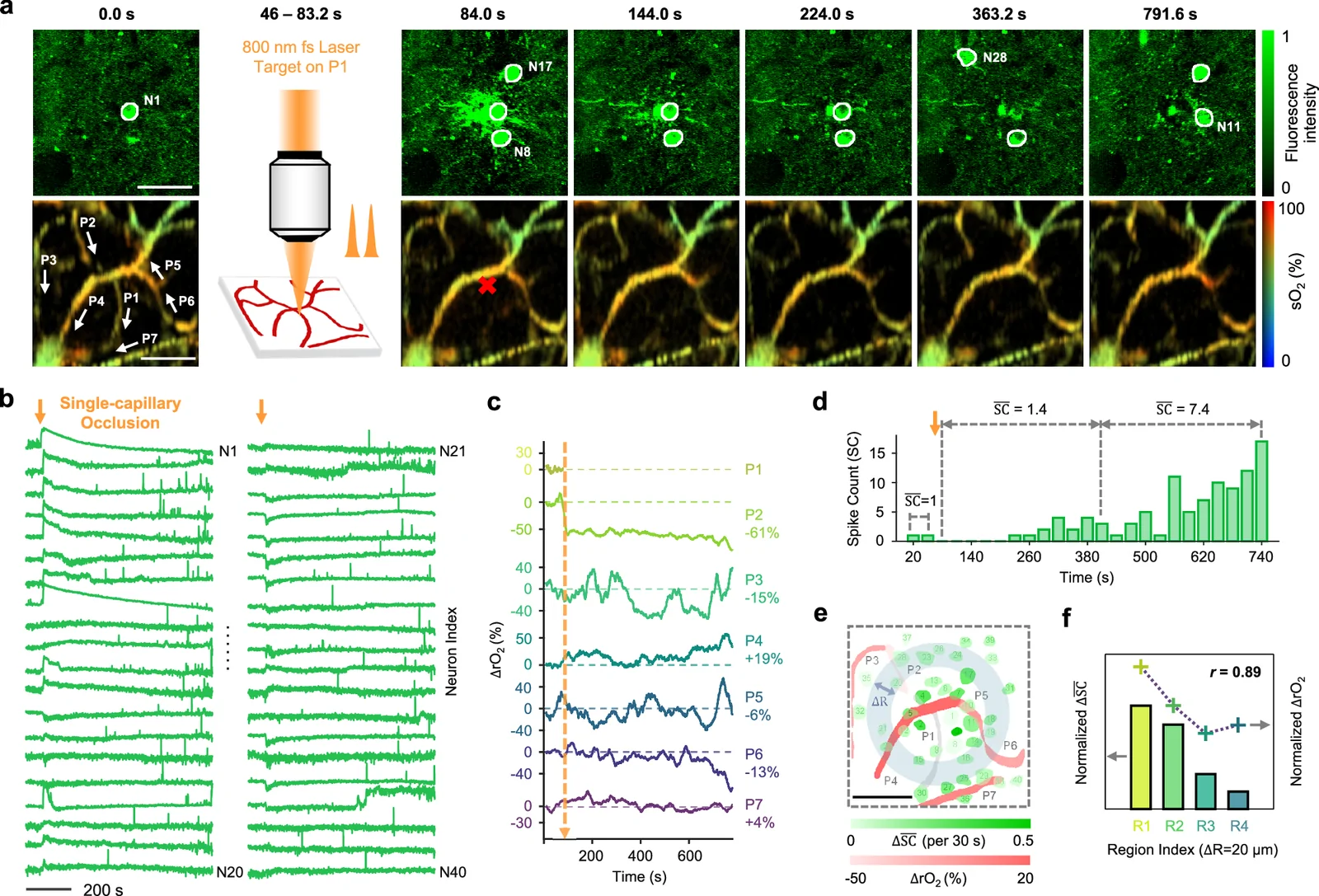
Neurometabolic responses to single-capillary occlusion in the awake GCaMP mouse brain. **a** Schematic of targeted occlusion in capillary P1, along with TPM-PAM images before and after occlusion. Top: TPM images of neuronal activity. Bottom: PAM images of sO_2_, with arrows indicating flow directions. Red cross marks the occlusion site. **b** Calcium activity traces from forty neurons within the FOV. The fluorescence intensity in each trace is normalized to its maximum value. **c** Temporal dynamics of rO_2_ from seven capillaries, expressed in relative changes. Numbers represent the average change in rO_2_ following occlusion. **d** Time course of summed spike count (SC) per 30 s across all neurons. $\small \overline{\rm{SC}}$ represents an average of SC over time. **e** Overlay of neuronal $\small \Delta\overline{\rm{SC}}$ map and vascular ∆rO_2_ map after occlusion. Individual neurons are numbered corresponding to the indices in (**b**). Blue shadow highlights annulus R3, which has a width of 20 µm. **f** Average responses of neuronal spike (bars) and vascular rO_2_ (connected markers) within each annulus. Correlation coefficient (*r*) quantifies the spatial coupling between neuronal activity and oxygen release. Scale bars: **a**, **e** 50 µm.

After occlusion, the target capillary (P1) disappeared from the vascular map, accompanied by sustained calcium activity in nearby neurons (Fig. 3a). Calcium traces were extracted from forty neurons within the FOV (Fig. 3b), along with concurrent time courses of rO_2_ measured in seven microvessels (Fig. 3c). Quantifying the total spike count across all neurons revealed a progressive elevation in the firing rate: the average spike count ($\small \overline{\rm{SC}}$) per 30 s increased from 1 before occlusion to 1.4 and 7.4 in the early and late post-occlusion phases, respectively (Fig. 3d).

Overlaying the maps of post-occlusion changes in average neuronal spike count ($\small \Delta\overline{\rm{SC}}$) and RBC oxygen release (ΔrO_2_) revealed a spatial gradient: neurons closer to the occlusion site (red cross in Fig. 3a) showed stronger activation (Fig. 3e). To quantify the spatial relationship between neuronal activity and rO_2_, the FOV was divided into four concentric annuli (R1–R4 in Fig. 3e; each 20 µm wide, with R3 highlighted in blue) centered on the occlusion site. The average responses of $\small \overline{\rm{SC}}$ and rO_2_ within each annulus were calculated and compared, and the high correlation coefficient (0.89) indicates a strong spatial coupling between enhanced neuronal activity and compensatory RBC oxygen release following focal ischemia (Fig. 3f).

### Neurometabolic responses to single-neuron stimulation

We next examined neuronally driven neurometabolic responses by applying single-neuron stimulation in awake, head-fixed GCaMP mice (Supplementary Movie 4). The stimulation was achieved by focusing high-energy, 800-nm, fs-laser pulses onto the membrane of a single neuron for 1 s, triggering localized calcium influx and subsequent neuronal activation.

**Fig. 4:**
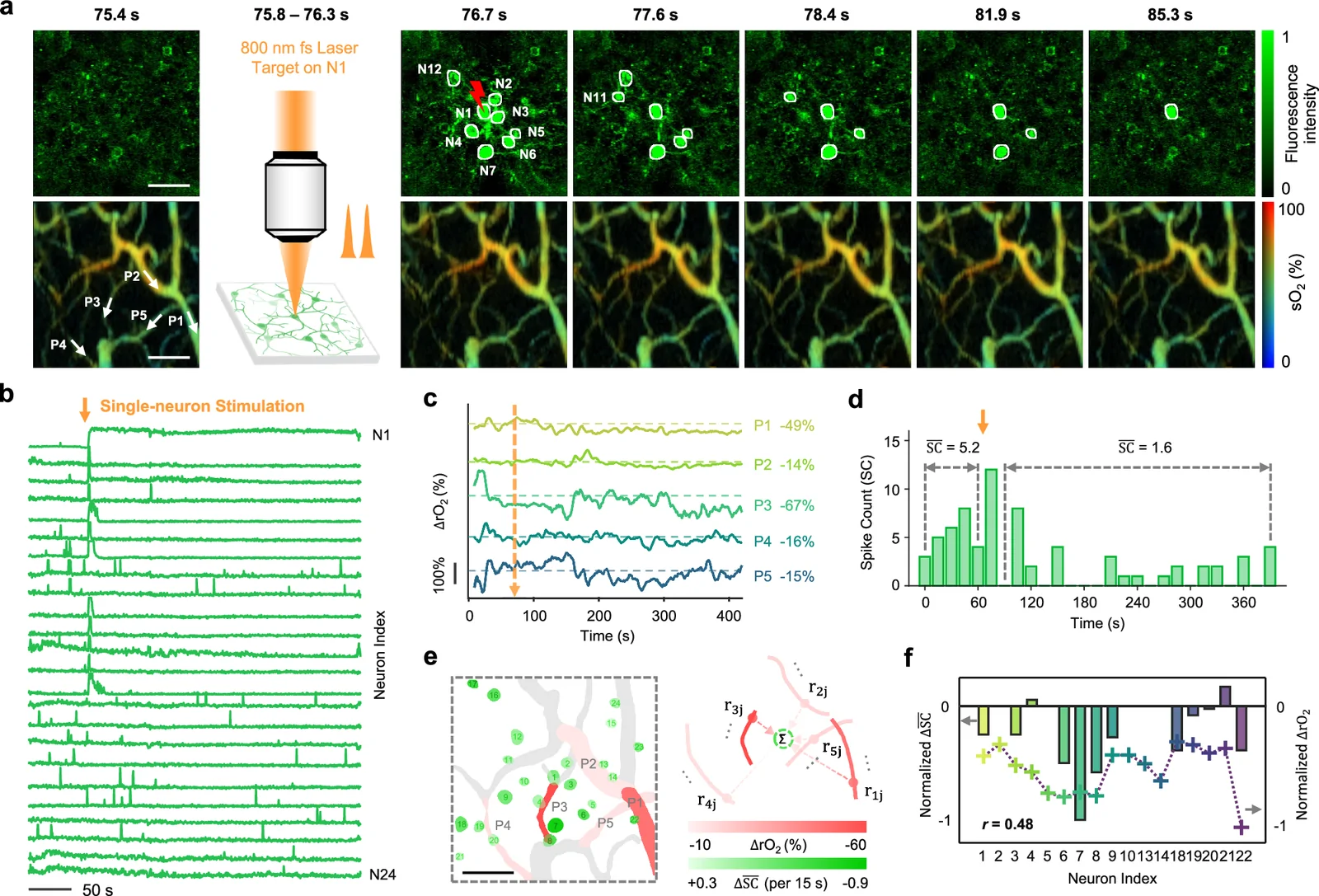
Neurometabolic responses to single-neuron stimulation in the awake GCaMP mouse brain. **a** Schematic of targeted stimulation in neuron N1, along with TPM-PAM images before and after stimulation. Top: TPM images of neuronal activity. Bottom: PAM images of sO_2_, with arrows indicating flow directions. Red lightning denotes the stimulation site. **b** Calcium activity traces from twenty-four neurons within the FOV. The fluorescence intensity in each trace is normalized to its maximum value. **c** Temporal dynamics of rO_2_ from five capillaries, expressed in relative changes. Numbers represent the average change in rO_2_ following stimulation. **d** Time course of summed spike count (SC) per 15 s across all neurons. $\small \overline{\rm{SC}}$ represents an average of SC over time. **e** Left: overlay of neuronal $\small \Delta\overline{\rm{SC}}$ map and vascular ∆rO_2_ map after stimulation. Individual neurons are numbered corresponding to the indices in (**b**). Right: schematic of computational diffusion to estimate oxygen delivery from nearby capillaries to a specific neuron. **f** Stimulation-induced changes in the neuronal spike count of individual neurons (bars) and the oxygen supply from nearby capillaries (connected markers). Correlation coefficient (*r*) quantifies the spatial coupling between neuronal activity and oxygen release. Scale bars: **a**, **e** 50 µm. Panel **a** contains elements created in BioRender. Han, J. (https://BioRender.com/4hepyhv).

As shown in the representative TPM-PAM images (Fig. 4a), stimulation of the target neuron (N1) elicited not only its own activation but also calcium responses in neighboring neurons. Given the highly confined nonlinear optical absorption volume, these secondary responses were likely due to synaptic network propagation rather than direct photostimulation. The calcium activity of twenty-four neurons and the oxygen release from five capillaries were recorded simultaneously (Fig. 4b, c). The summed neuronal spike count over time revealed a transient population-level surge immediately after stimulation, followed by a prolonged suppression phase (Fig. 4d). The average spike count ($\small \overline{\rm{SC}}$) per 15 s reduced from 5.2 before stimulation to 1.6 afterward, in contrast to the enhanced firing rate observed following the single-capillary occlusion (Fig. 3d).

To further examine the relationship between neuronal activity and RBC oxygen release, we overlaid the $\small \Delta\overline{\rm{SC}}$ and ΔrO_2_ maps following single-neuron stimulation (Fig. 4e). The oxygen released from individual capillaries was computationally diffused to the locations of nearby neurons (see the Methods for details) to estimate the relative change in oxygen supply to each neuron after stimulation (Fig. 4e). The resulting correlation between the neuronal and vascular responses yielded a coefficient of 0.48 (Fig. 4f), indicating a moderate coupling between reduced neuronal activity and decreased local oxygen release. Data from neurons whose oxygen supply originated from capillaries with unmeasured rO_2_ (i.e., the four in the upper left and three in the upper right regions of the FOV)—due to limited acquisition time (see the Discussion for details)—were excluded from the analysis.

## Discussion

Neurometabolic coupling (NMC), the dynamic process by which neuronal activity is matched with local oxygen delivery from nearby RBCs, forms the physiological basis of brain function. While global features of NMC have been extensively studied using macroscopic imaging^34,35^, the cellular mechanisms governing oxygen exchange between individual neurons and RBCs remain poorly understood. Bridging this spatial scale in neurometabolic imaging is critical for understanding how the brain allocates metabolic resources to active neurons and how disruptions in this process contribute to neurological disorders^36^.

Our method directly addresses this challenge by integrating high-resolution optical and photoacoustic imaging within a unified platform. Through the incorporation of a transparent MRR as an ultrasound detector, TPM-PAM overcomes the long-standing optical-acoustic tradeoff in multimodal imaging, achieving high photoacoustic sensitivity while preserving full optical access for two-photon excitation and fluorescence collection. This design enables not only simultaneous visualization of individual neurons and RBCs in vivo, but also quantitative measurements of calcium activity and multiple oxygen-metabolic parameters.

Building upon this light-sound synergy, we further established a quantitative framework to measure the oxygen release rate from individual RBCs, allowing direct assessment of local oxygen delivery dynamics. Correlating RBC oxygen release with neuronal calcium activity opens new opportunities to interrogate the metabolic underpinning of the neurovascular unit in awake animals. Moreover, the incorporation of ultrafast laser-based optical manipulation further extends the versatility of this platform, allowing precise induction of targeted single-capillary occlusion and single-neuron stimulation. Together, these capabilities enable both monitoring and perturbation of neurovascular interactions under precisely controlled conditions, establishing an experimental platform for causal dissection of neurometabolic regulation at the cellular level in vivo.

The TPM-PAM platform revealed neurometabolic insights inaccessible to existing methods. In whisker stimulation, TPM-PAM not only replicated prior TPM findings of arteriolar dilation and capillary flow increase^12,37,38^, but also provided the first comprehensive quantification of oxygen dynamics across different vessel types. Arterial oxygen supply, reflected by fO_2_, increased significantly following neuronal activation, driven primarily by vasodilation. This upstream response elevated both sO_2_ and V_f_ in downstream capillaries and venules, resulting in a 113% ± 48% increase in capillary oxygen release to meet the metabolic demand of activated neurons. The excess oxygen delivery beyond local consumption drained through venules. Moreover, we observed that V_f_ responses preceded changes in both sO_2_ and C_Hb_, providing direct experimental support for Buxton’s balloon model^39^, which suggests that flow speed rises immediately with arteriolar dilation, while blood oxygenation evolves more gradually due to vascular compliance, finite blood transit time, and diffusion-limited oxygen exchange. In both single-capillary occlusion and single-neuron stimulation, the RBC oxygen release rate (rO_2_) showed a moderate to strong correlation with local neuronal activity—providing a first quantitative link between oxygen consumption and neuronal metabolic demand at the cellular scale. Moreover, the distinct neuronal responses following the vascular vs. neuronal perturbations imply different compensatory mechanisms, such as network-driven collateral recruitment during vascular blockade^40^ vs. feed-forward neurovascular signaling during neuronal activation^6^. Generalizing these local, micro-scale neurovascular responses will require future studies explicitly designed to map, rather than average out, the intrinsic heterogeneity of synaptic connectivity and capillary network topology in NVC and NMC^41^. In addition, these manipulation experiments hold the potential to establish bidirectional neurovascular causality and provide mechanistic insights that refine the interpretation of hemodynamics-based neuroimaging signals. Furthermore, targeted single-capillary occlusion provides a controllable model of microvascular ischemia for studying neurovascular dysfunction and evaluating therapeutic strategies in neurodegenerative disease^42^.

Future developments could further enhance the capabilities of TPM-PAM. On the TPM side, dynamic imaging is currently restricted to a fixed depth because mechanical axial scanning is too slow to capture volumetric calcium dynamics. As a result, the current implementation might miss information from neurons located within a capillary’s oxygen diffusion volume but outside the TPM focal plane. This mismatch in imaging volume between TPM and PAM might lead to partial sampling of neurovascular interactions and thus compromise the accuracy of spatial analysis. This limitation could be addressed by implementing advanced strategies, such as axially extended point spread function^43–45^ or temporal multiplexing^46,47^, to enable simultaneous recording of neuronal activity across multiple depths. On the PAM side, light scattering at visible wavelengths limits the imaging depth to less than 0.5 mm. Near-infrared (NIR) PAM may alleviate this issue^48^, but its performance in multi-parametric microvascular imaging is hindered by weaker blood absorption in this spectral range and thus lower signal-to-noise ratio. The development of next-generation MRR-based ultrasound sensors with greater sensitivity may overcome this barrier. In addition, while dual-wavelength kymographic scanning enables quantitative rO_2_ mapping across multiple capillaries, it requires sequential interrogation of individual vessels. Because PAM and TPM currently share the same scanning scheme, the number of vessels covered by the kymographic scanning is constrained by the temporal resolution required for TPM-based calcium imaging. This limitation could be mitigated by decoupling the TPM and PAM scanning schemes—using dual galvanometer mirrors for high-speed raster scanning in TPM, while alternating between raster and kymographic scanning in PAM—to increase the throughput of rO_2_ measurements without compromising the speed of calcium imaging.

Future refinement of the optical manipulation approaches would broaden the utility of TPM-PAM. The current fs laser-based perturbations produce irreversible vascular or neuronal effects, limiting studies of recovery or repetitive stimulation. Optogenetics offers a promising alternative for reversible and cell-type-specific modulation of neuronal^49,50^ and vascular^51,52^ activity. However, most opsins have broad absorption spectra that overlap with the green excitation used in PAM, introducing spectral crosstalk between imaging and manipulation. This challenge could be mitigated by engineering red-shifted or narrow-spectrum opsins or implementing NIR PAM to increase the spectral separation between imaging and stimulation.

In summary, TPM-PAM establishes a versatile, quantitative platform for all-optical imaging and manipulation of the neurovascular unit at single-cell resolution in awake mouse brains. By directly linking single-neuron activity with localized oxygen release from individual RBCs, this method opens new avenues for mechanistic studies of neurometabolic coupling at an unprecedented spatial scale. With continued advances in instrumentation and analysis, TPM-PAM may enable broader investigations into cellular energy metabolism, not only in the brain but potentially in other organ systems.

## Methods

### Integrated TPM-PAM

The optical layout of the integrated TPM-PAM system is illustrated in Supplementary Fig. 4. In the light path of TPM, a fs laser tuned at 930 nm (Coherent Chameleon Discovery) is expanded and collimated using a pair of lenses (L1 and L2; Thorlabs AC254-030-AB and AC254-200-AB, respectively). A water-immersion objective (OBJ; Olympus XLPlan N, 25×, NA = 1.05) is utilized to focus the beam for two-photon excitation. The emitted fluorescence is collected via the same objective, separated by a dichroic mirror (DM3; Semrock Di03-R532-t1) and passes through an electrical shutter (ES2; Thorlabs SHBH1T). The green fluorescence from GCaMP is then reflected by a dichroic mirror (DM5; Chroma T560lpxr), filtered through a bandpass filter (BPF3; Semrock FF01-503/40-25), and focused onto a photomultiplier tube (PMT1; Thorlabs PMT2101) by a lens (L7; Thorlabs AC254-030-AB). The red fluorescence from Texas Red, which is used only in the single-capillary occlusion experiment for TPM of the microvascular structure, passes through DM5, is filtered by a bandpass filter (BPF4; Semrock FF01-632/148-25), and is focused on a photomultiplier tube (PMT2; Thorlabs PMT2101) by a lens (L8; Thorlabs AC254-030-AB). For the collection of red fluorescence, DM3 is replaced by another dichroic mirror (DM6; Thorlabs DMLP650R). In the light path of PAM, a 532-nm ns laser (Spectra-Physics VGEN-G-20), operating at a repetition rate of 80 kHz, is directed through an electro-optic modulator (EOM; Conoptics 350-50-01-RP) for polarization modulation. A half-wave plate (HWP1; Thorlabs WPHSM05-532) is placed before the EOM to rotate the laser polarization to the horizontal direction, with the EOM’s crystal axis oriented at 45° relative to the input polarization. When zero voltage is applied to the EOM, the output light is still horizontally polarized and passes through the polarizing beamsplitter (PBS1; Thorlabs PBS121). A second pair of HWP2 and PBS2 (Thorlabs WPHSM05-532 and PBS121) is used to control the transmitted light pulse energy, with the reflected light absorbed by a beam blocker (BB1; Thorlabs LB1). The transmitted light is then coupled into a polarization-maintaining fiber (PMF1; Thorlabs PM-S405-XP) for wavelength conversion via stimulated Raman scattering and filtered by a bandpass filter (BPF1; Chroma ET546/10x) to isolate the 545-nm component. When a high voltage is applied to the EOM, the polarization of the output light is rotated by 90° and thus reflected by PBS1 to the second Raman path to isolate the 558-nm component through another bandpass filter (BPF2; Thorlabs FBH560-10). The dual-wavelength laser pulses are combined by a dichroic mirror (DM4; Semrock FF552-Di02) and alternated pulse-by-pulse during imaging to quantify sO_2_. A beam sampler (BS; Thorlabs BSN10R) and a photodetector (PD1; Thorlabs PDA36A) are utilized to monitor the pulse energy fluctuation. The PAM and TPM excitation beams are combined through a dichroic mirror (DM2; Chroma ZT647rdc) and focused by the same microscope objective onto the mouse brain. The diameter of PAM excitation beam is 1 mm, corresponding to an effective NA of 0.1. The foci of the PAM and TPM excitation are aligned in both lateral and axial directions, ensuring co-registered imaging across the entire FOV. The generated ultrasonic wave is detected by a transparent micro-ring resonator (MRR; Supplementary Fig. 2) placed directly beneath the objective. The space between the objective and the MRR, as well as that between the MRR and the mouse brain, is filled with water. A CW laser (Newport TLB-6712) is coupled into the MRR for signal interrogation, and the transmitted light intensity is detected by a photodetector (PD2; Thorlabs PDB465A-AC). The polarization of the CW input is modulated by a polarization controller (PC; Thorlabs FPC030) to optimize the modulation depth of the MRR resonance. The AC output of PD2 is sampled via a high-speed data acquisition card (AlazarTech ATS9350) after being filtered by an electrical high-pass filter (HPF; Thorlabs EF125), while the DC output is monitored by a field programmable gate array (FPGA, National Instruments PXIe-7842r) to provide feedback for real-time tuning of CW laser wavelength to compensate for the thermal drift of the MRR. In the optical manipulation part, a second fs laser tuned at 800 nm (Coherent Chameleon Vision-S) is expanded and collimated using a pair of lenses (L3 and L4; Thorlabs AC254-030-AB and AC254-200-AB, respectively), combined with the imaging laser through a dichroic mirror (DM1; Chroma T810lpxr) and focused by the same objective onto the mouse brain for single-capillary occlusion or single-neuron stimulation. All three light foci are aligned both laterally and axially. An electrical shutter (ES1; Thorlabs SH05) is placed in the light path to control the ON/OFF state of the optical manipulation. In the TPM-PAM system, a dual-axis galvanometer (GM; Thorlabs QS10XY-AG) is used to scan the light beams for imaging. The beams are relayed to the pupil plane of the objective by a pair of lenses (L5 and L6; Thorlabs AC508-180-AB and AC508-300-AB, respectively) in a 4f configuration. All hardware in this system is controlled and synchronized by the FPGA, and all imaging data are acquired using the data acquisition card (Supplementary Fig. 11).

### MRR-based ultrasound detection

The polymer MRR-based ultrasound sensor consists of a micro-ring and a bus waveguide fabricated on a SOG dielectric material-coated glass substrate using soft nanoimprint lithography (Supplementary Fig. 2a). A single-mode fiber and a multi-mode fiber are precisely aligned to the input and output waveguide facets, respectively, to couple the wavelength-tunable CW laser into the MRR and to route the transmitted light to a photodetector for monitoring ultrasound-induced resonance shift. For ultrasound detection, the wavelength of the CW laser, which serves as the light source for MRR interrogation, is tuned to the point of maximum slope on the resonance spectrum (Supplementary Fig. 2b). Incident ultrasound pressure deforms the polymer MRR, changing the effective optical path length and shifting its resonance spectrum. This shift modulates the intensity of the transmitted light at the interrogation wavelength, and the modulated signal is recorded by the photodiode. This detection mechanism provides a broad bandwidth up to 100 MHz (Supplementary Fig. 2c) and a high sensitivity over a considerable FOV (Supplementary Fig. 2d). The packaged MRR is optically transparent and only 370 μm thick. Thus, it can be directly placed beneath the high-NA objective without degrading the optical focus (Supplementary Fig. 2e). In the TPM-PAM system, the packaged MRR is mounted on a 3D-printed holder (Supplementary Fig. 12) attached to a 3-axis translation stage. Before in vivo imaging, the MRR is positioned at the center of the FOV by imaging a piece of black tape and adjusting the translation stage to maximize the photoacoustic signal amplitude at the center. During imaging, thermal effects can gradually shift the MRR’s resonance wavelength, leading to signal instability. To address this issue, the DC output of the photodiode is sampled by the FPGA every 0.5 ms. When the DC signal deviates from the preset value beyond a defined threshold, the FPGA adjusts the CW laser wavelength via a PZT motor in small steps to track the resonance position. The tuning direction is determined by the side of the resonance slope used for locking and the relative difference between the current DC voltage and the set value (Supplementary Fig. 11b). This real-time feedback control effectively compensated for the thermal drift, ensuring stable and high-fidelity ultrasound detection throughout imaging sessions.

### Kymographic scanning

In this work, raster scanning was used to image neuronal activity, as well as microvascular C_Hb_ and sO_2_, across the entire FOV, while kymographic scanning was applied to measure single-RBC sO_2_, flow speed, and oxygen release rate within user-selected microvessels. All experiments used a combined scanning strategy, interleaving one kymographic scan after every two consecutive raster scans.

Here is the procedure for designing the kymographic scanning path:

Step 1: Perform an initial raster scan to obtain a PAM image of the vasculature.

Step 2: Visualize the acquired image in MATLAB and manually define an initial kymographic path. This path forms a closed loop covering the central axes of all vessels of interest, typically consisting of 300–600 uniformly spaced sampling points.

Step 3: Load the path into LabVIEW to drive the galvanometer for kymographic scanning, where the light focus is repeatedly scanned along the path to evaluate whether the blood flow in each vessel is resolvable (i.e., whether a clear RBC motion slope is visible in the kymograph). With an 80-kHz laser pulse repetition rate, a path with 300 sampling points corresponds to a line rate of 267 Hz. Assuming a maximum distinguishable RBC displacement of 10 µm between adjacent lines, this line rate translates to a maximum resolvable flow speed of ~2.7 mm/s. While sufficient for most capillaries, this may fall short for measuring faster flow in some arteries.

Step 4: Refine the path design by assigning an independent closed loop to each vessel whose flow is too fast to be resolved in Step 3, while grouping the remaining vessels into a separate loop (e.g., allocating 100 sampling points for a fast-flow vessel and 200 for the rest). Each loop is repeatedly scanned for ~40 cycles before the system proceeds to the next loop. This multi-loop strategy effectively increases the line rate for fast-flow vessels (from 267 Hz to 800 Hz in this work), thereby extending the maximum resolvable flow speed from ~2.7 mm/s to ~8 mm/s. The final path typically consists of 1–4 loops with step sizes of 2–5 µm. Notably, simply applying a high line rate (e.g., 800 Hz) to all vessels via a single large loop is not preferred, as the galvanometer may not reliably track the large loop at such a high speed. Instead, assigning a high line rate only to fast-flow vessels within relatively small spatial regions ensures galvanometer stability.

Step 5: Perform TPM-PAM imaging experiment using the optimized multi-loop path, with every two raster scans followed by one kymographic scan.

The scanning parameters and paths for all imaging experiments in this work are summarized in Supplementary Table 1 and Supplementary Fig. 13. Using the parameters from Fig. 1f-k as an example, the imaging field of view (FOV) is 150 × 150 µm^2^ with 150 × 150 pixels, corresponding to a step size of 1 µm. Given a PAM laser repetition rate of 80 kHz, each raster scan takes 0.28 s. After two raster scans, a kymographic scan is performed using two closed loops with 100 and 350 sampling points (pts.), which correspond to line rates of 800 Hz and 230 Hz, respectively. Each loop is repeated for 40 cycles (c.). Each kymographic scan takes 0.23 s. Together, this combined scanning strategy yields an effective raster frame rate of 2.54 Hz and an effective kymography frame rate of 1.27 Hz.

### Imaging parameters

For all experiments in this work, the PAM nanosecond laser operated at a repetition rate of 80 kHz with a pulse energy of 100 nJ. The TPM femtosecond laser operated at 80 MHz with an average power of 40 mW. The FOV for all dynamic imaging was set to 150 × 150 or 200 × 200 µm^2^ with a fixed pixel number of 150 × 150, corresponding to an imaging frame rate of 3.56 Hz and an effective imaging frame rate of 2.54 Hz when considering kymographic scanning. A detailed summary of the key parameters for each experiment is provided in Supplementary Table 1. The imaged neurons and blood vessels all located within a depth range of approximately 100–300 µm below the dura (cortical layers II/III) in all experiments presented in this work.

For whisker stimulation, we performed imaging and 10 trials of stimulation on 1 mouse on the same day. The results in Fig. 2 are derived from responses of 9 neurons and 8 vessels across all 10 trials. For single-capillary occlusion and single-neuron stimulation, we performed imaging and 1 trial of manipulation on 1 mouse in each experiment. The results in Figs. 3 and 4 are derived from responses of 40 neurons and 7 capillaries, and 24 neurons and 5 capillaries, respectively.

### Field-of-view selection

For whisker stimulation, imaging was performed in the primary somatosensory barrel cortex. To identify an optimal FOV, we manually surveyed the cortical tissue using a 3D translation stage during real-time imaging and repeated whisker stimulation. The final FOV was selected based on the following criteria: (1) at least one neuron within the FOV exhibited robust activation in response to stimulation; (2) the FOV contained all major vessel types (i.e., arterioles, venules, and capillaries) to enable comparative analysis of their distinct responses.

For single-capillary occlusion and single-neuron stimulation, the FOV was selected based on a single criterion: the vascular network within the FOV was predominantly composed of capillaries rather than large vessels, as the primary focus here was to analyze oxygen release from capillaries to neurons.

### Whisker stimulation

Whisker stimulation was performed using a foam brush mounted on a rotary stepper motor (Lin Engineering ZH417-11-06) to deflect the whiskers of an awake GCaMP mouse at 4 Hz for 5 s (Supplementary Fig. 12). The motor was driven by a function generator operating in burst mode, which was triggered by the FPGA. The round-trip rotation angle was set to 20°, providing consistent whisker deflection across trials. In this experiment, 10 stimulation trials were continuously conducted with a 40-s interval.

### Single-capillary occlusion

Previous studies have shown that nonlinear absorption of ultrashort laser pulses by biological tissues can induce mechanical perturbations, including cavitation bubble formation and shock wave generation, with their size and strength dependent on laser energy. High-energy fs pulses can stress endothelial cells and the basement membrane, trigger the endogenous clotting cascade, and ultimately lead to intravascular clot formation^31^. Because of the femtoliter-scale nonlinear absorption volume, the interaction can be spatially confined within a single vessel without damaging the surrounding tissue. In this work, a fs laser tuned at 800 nm was utilized to induce intravascular clot in a single capillary. To ensure the laser focus precisely targeted on the capillary of interest, the fluorescein-dextran (Texas Red, 70000 MW, 0.3 ml, 5% wt/vol in saline) was used to label the blood plasma of the experimental mouse through intraocular injection. The fs laser was then utilized for TPM of the microvascular structure in the red fluorescence channel, during which the target capillary was positioned at the center of the FOV, both laterally and axially, by manually adjusting the mouse position using a 3-axis translation stage. Afterward, TPM-PAM imaging was initiated to monitor the neurometabolic dynamics before the onset of the capillary occlusion. Although PAM can also provide vascular information to position the target capillary in the lateral direction, its axial resolution was larger than the nonlinear absorption range and thus insufficient for accurate axial localization. To induce occlusion, the electrical shutter in the fs laser path was opened for 1 s, with the laser focus fixed at the center of the FOV. The laser power was set to ~100 mW, with a pulse repetition rate of 80 MHz. Then, the shutter was closed, and TPM-PAM imaging resumed to monitor the status of the target capillary. The alternating sequence of occlusion induction and imaging was automatically controlled by the FPGA and repeated with progressively increased duration until successful occlusion was achieved. During occlusion induction, the shutter in the fluorescence channel remained closed to prevent possible strong light leakage or fluorescence that could trip or even damage the photodetector. After successful single-capillary occlusion, TPM-PAM imaging continued for 10 min to monitor the neurometabolic responses. This study was performed in an awake GCaMP mouse.

### Single-neuron stimulation

Targeted stimulation of single-neuron using a focused fs laser has been demonstrated in cultured neuronal networks^32,33^. Transient disruption and restoration of the neuronal membrane at the laser spot by nonlinear optical absorption can induce an influx of extracellular Ca^2+^ to trigger neuronal activation^32^. In this work, the same 800-nm fs laser used for single-capillary occlusion was utilized for single-neuron stimulation in an awake GCaMP mouse. The laser power was set to ~100 mW, and the target neuron was positioned at the center of the FOV. The laser focus was maintained on the neuron for 1 s to achieve stimulation. Following the stimulation, TPM-PAM imaging was performed for 5 min to monitor the neurometabolic responses.

### Resolution calibration

An absorption resolution target (provided by Dr. Jeeseong Hwang at the National Institute of Standards and Technology) and a fluorescent target (Edmund 57-855) were used to calibrate the spatial resolution of PAM and TPM in the integrated platform, respectively (Supplementary Fig. 5). The lateral resolution was quantified as the full-width-half-maximum (FWHM) value of the line spread function (LSF) derived from the edge spread function (ESF). PAM’s axial resolution was defined as the FWHM of the Hilbert-transformed A-line signal, while TPM’s axial resolution was quantified by scanning a 0.5 μm fluorescent bead (Invitrogen FluoSpheres carboxylate) along the axial direction using a stepper motor (PI L-509) and extracting the FWHM value of the fluorescence intensity profile.

### Mouse preparation

All experiments in this work were performed on GCaMP mice under a craniotomy preparation for in vivo imaging. Prior to craniotomy, the mouse was first anesthetized with vaporized isoflurane and secured in a stereotaxic surgical station with 1.5% isoflurane. The body temperature was kept at 37 °C using a heating pad. Carprofen (5 mg/kg) was administered to mitigate inflammation and swelling of the brain in response to craniotomy. The scalp was disinfected and then removed with surgical scissors, and a ~ 3-mm-diameter cranial window was created on the right side of the brain using a dental drill. For the whisker stimulation experiment, the window was centered over the primary somatosensory barrel cortex ( ~ 1 mm posterior and ~3 mm lateral to the bregma). After skull removal, surgical sponges were applied to stop bleeding, and epoxy resin was used along the edge of the cranial window to prevent bone regrowth. A 100 µm-thick acrylic film (Emco Industrial Plastics) was then placed over the exposed brain and sealed around the edge with cyanoacrylate glue. After the glue was cured for 10 minutes, the mouse was returned to its home cage for recovery. Buprenorphine (0.1 mg/kg) was administered daily for 3 days post-surgery to alleviate pain and inflammation. Ten days after craniotomy, the mouse skull was gently scraped with a dental drill to improve adhesion. Then, a 3D-printed metal head frame (Supplementary Fig. 12c) was adhered to the skull using C&B-METABOND (Parkell; mixture of Radiopaque L-Powder, “B” Quick Base, and “C” Universal TBB Catalyst) followed by 2 hours of curing. Throughout the procedure, the mouse was maintained under anesthesia with 1.5% isoflurane and kept at 37 °C with a heating pad. One day later, the mouse was acclimated to head fixation by training on an air-floating treadmill for 3 h, with its head restrained by the frame. Awake-brain imaging was performed after three consecutive days of training. All experimental procedures were carried out in conformity with the animal protocol approved by the Institutional Animal Care and Use Committee at Washington University in St. Louis.

### Data analysis

#### Multi-parametric quantification of the oxygen-metabolic dynamics

- Diameter (D, mm): A binary mask is generated for each vessel by applying a photoacoustic amplitude threshold defined as twice the noise level. The vessel skeleton is extracted from the mask, and the diameter is calculated as the mask area divided by the skeleton length.
- Blood flow speed (V_f_, mm/s): The kymograph acquired in this work represents the spatiotemporal trajectories of RBCs, with the horizontal axis corresponding to spatial position (x-axis) and the vertical axis representing time (t-axis). As RBCs move, they create distinct diagonal streaks across the kymograph. Therefore, the orientation angles of these motion streaks directly encode the RBC flow speed. The Radon transform is a mathematical tool applied to extract the dominant orientation angle objectively and accurately^53^. As shown in Supplementary Fig. 6, the Radon transform is applied to project the kymograph over a range of angles, and the variance is then calculated for each projection. The angle corresponding to the maximum variance, denoted as ${{\rm{\theta }}}_{{\rm{peak}}}$, represents the dominant streak orientation. Finally, the flow speed is calculated based on the orientation angle by ${{\rm{V}}}_{{\rm{f}}}=\Delta {\rm{x}}/\Delta {\rm{t}}/\tan ({{\rm{\theta }}}_{{\rm{peak}}})$, where ∆x and ∆t are the pixel size along the x and t axes, respectively.
- Volumetric blood flow speed (CBF, mm^3^/s): ${\rm{CBF}}={\rm{\pi }}{{\rm{D}}}^{2}{{\rm{V}}}_{{\rm{f}}}/8$.
- Oxygen saturation of hemoglobin (sO_2_, %): Spectral analysis of the dual-wavelength (545 nm and 558 nm) A-line pair is used to differentiate oxy-hemoglobin (HbO_2_) and deoxy-hemoglobin (HbR), from which sO_2_ is computed.
- Relative concentration of hemoglobin (C_Hb_, a.u.): C_Hb_ is represented by the photoacoustic amplitude at 545 nm, which is an isosbestic point for HbO_2_ and HbR.
- Oxygen flux rate (fO_2_, a.u.): ${{\rm{fO}}}_{2}={\rm{CBF}}\times {{\rm{C}}}_{{\rm{Hb}}}\times {{\rm{sO}}}_{2}$. fO_2_ represents the amount of oxygen flowing through a vessel per unit time.
- Oxygen release rate (rO_2_, a.u.): The rO_2_ quantification procedure consists of 7 steps (S1–S7), as illustrated in Supplementary Fig. 3. S1: Compute V_f_ through the kymographic analysis. S2: Separate individual RBC clusters and generate boundary masks using the algorithm described in Supplementary Fig. 14. S3: Sum the photoacoustic amplitudes within each cluster, and the result n_(i)_ represents the relative RBC content in individual clusters. S4: Generate an sO_2_ kymograph through spectral analysis of the dual-wavelength raw data. S5: Apply the boundary masks generated in S2 to the sO_2_ kymograph. S6: Perform linear fitting on the sO_2_ variation along the x axis to compute ${\nabla {\rm{sO}}}_{2({\rm{i}})}$ for each cluster. The ${\nabla {\rm{sO}}}_{2}$ is defined as the sO_2_ drop per unit distance along the flow direction. S7: ${{\rm{rO}}}_{2}=\mathop{\sum }\limits_{{\rm{i}}=1}^{{\rm{N}}}{\rm{k}}\times {\rm{c}}\times \nabla {{\rm{sO}}}_{2({\rm{i}})}\times {{\rm{V}}}_{{\rm{f}}}\times {{\rm{n}}}_{({\rm{i}})}$, where k and c are constants that represent the oxygen-binding capacity of hemoglobin and the hemoglobin content in RBC, respectively, and N is the number of clusters. rO_2_ stands for the amount of oxygen released from a specific capillary to surrounding tissues per unit time and is computed at the single-RBC level.
- Oxygen extraction fraction (OEF, %): ${\rm{OEF}}=({{\rm{s}}}_{{\rm{a}}}{{\rm{O}}}_{2}-{{\rm{s}}}_{{\rm{v}}}{{\rm{O}}}_{2})/{{\rm{s}}}_{{\rm{a}}}{{\rm{O}}}_{2}$, where ${{\rm{s}}}_{{\rm{a}}}{{\rm{O}}}_{2}$ and ${{\rm{s}}}_{{\rm{v}}}{{\rm{O}}}_{2}$ are the average sO_2_ values in feeding arteries and draining veins, respectively. OEF represents the ratio of oxygen consumption to total oxygen supply across the entire FOV.

To clarify, among the above parameters, vessel diameter (D), flow speed (V_f_), oxygen saturation (sO_2_), hemoglobin concentration (C_Hb_), and oxygen release rate (rO_2_) are directly measured, while volumetric blood flow (CBF), oxygen flux rate (fO_2_), and oxygen extraction fraction (OEF) are derived from those direct measurements.

#### Quantification of the response times of the individual oxygen-metabolic parameters

- Peak time: Taking the stimulation onset as time zero, the peak time is defined as the time point at which the response reaches its maximum amplitude (V_max_). If multiple peaks with amplitudes exceeding 90% of V_max_ occur within [0, 15] s, the peak time is calculated as the average of these time points. The peak time value will be regarded as unreliable in the following cases and will not be used for subsequent statistical analysis: (i) the peak of the spontaneous response within [−10, 0] s is larger than 50% of V_max_; (ii) the V_max_ is less than 3 times the standard deviation of the baseline fluctuation, indicating a very weak response.
- Rise time: Rise time is defined as the duration over which the response increases from 10% to 90% of V_max_. It will be excluded from statistical analysis if the peak time in the same trial is deemed unreliable.
- Decay time: Decay time is defined as the duration over which the response decreases from 90% to 10% of V_max_. It will be excluded from statistical analysis if the peak time in the same trial is deemed unreliable.

#### Statistical analysis of the oxygen-metabolic response times

- Paired *t*-test between two parameters: The response time of two specific parameters in the same trial and from the same vessel is regarded as paired data. The paired *t*-test is performed to determine whether a significant difference exists between different parameters.
- Unpaired *t*-test between two vessel types under the same parameter: All vessels in the FOV are classified into three distinct vessel types (i.e., arteriole, venule, and capillary). If more than one vessel type shows a response for the same parameter, an unpaired *t*-test is performed to determine whether a significant difference exists between different vessel types.
- To ensure the validity of the statistical model, we assessed the normality of individual data distributions before performing the *t*-tests shown in Fig. 2f, g. Specifically, the normality was assessed using the Lilliefors test (*lillietest* function in MATLAB), which provides a test decision for the null hypothesis that the data comes from a distribution in the normal family against the alternative that it does not come from such a distribution. In all cases, the test returned a value of 0, indicating that the null hypothesis cannot be rejected at the 5% significance level. These results support the assumption of a normal distribution and the validity of applying the *t*-test in our analysis.

#### Time gating for fluorescence crosstalk removal

The ns laser used for PAM excitation can generate strong crosstalk in the TPM fluorescence detection channel, primarily arising from two sources: (i) stray light (e.g., the backscattered light at the air-lens interfaces of the microscope objective) of the 545/558 nm laser bypassing the rejection band of the TPM spectral filter (503/40) due to non-normal incidence angles, and (ii) anti-Stokes fluorescence^54^ from the GCaMP fluorophore (emission peak near 509 nm, within the transmission band of the TPM spectral filter) excited by the 545/558 nm laser. Because the trigger timing of each ns pulse is known, this crosstalk can be removed through time gating (Supplementary Fig. 15a). In this work, the PAM laser has a pulse repetition rate of 80 kHz, corresponding to a 12.5 μs interval between adjacent crosstalk signals. Thus, a gating window of 1 μs is applied, which is sufficient to eliminate crosstalk during in vivo imaging (Supplementary Fig. 15b).

#### Detection of neuronal spikes

A maximum-intensity projection is first applied across frames to visualize all active neurons. Then, the center of each neuron is manually identified, and a circular ROI (20 μm in diameter) is defined. The mean fluorescence intensity within each ROI was extracted over time, and peaks meeting the following two criteria were identified as neuronal spikes: (i) the peak intensity exceeded twice the noise level; (ii) the activity duration was shorter than 2 s to exclude the non-transient events.

#### Analysis of neurometabolic coupling

- Single-capillary occlusion: The entire FOV is divided into four concentric annuli (R1-R4), each with a width of 20 μm, centered on the occlusion site. The average neuronal spike response after the occlusion is computed for each annulus and normalized to its area. The mean vascular rO_2_ response is then computed and correlated with the neuronal response.
- Single-neuron stimulation: The average rO_2_ value of each microvessel before stimulation is first calculated as rO_2(i)_, $1\le i\le 5$. The vessel skeleton is extracted as a binary mask and then encoded with rO_2(i)_. To quantify the total amount of oxygen delivered to a specific neuron, oxygen diffusion from each vascular pixel on the coded mask to the centroid of the neuron is modeled by following the exponential oxygen diffusion model^55^ as $\mathop{\sum }\limits_{{\rm{i}}=1}^{5}\mathop{\sum }\limits_{{\rm{j}}=1}^{{{\rm{N}}}_{{\rm{i}}}}{{\rm{rO}}}_{2({\rm{i}},{\rm{j}})}\times \exp (-{{\rm{d}}}_{{\rm{ij}}}/{\rm{L}})$, where ${{\rm{N}}}_{{\rm{i}}}$ is the number of pixels along the skeleton of the i^th^ vessel, ${{\rm{d}}}_{{\rm{ij}}}$ is the distance from the vascular pixel (${\rm{i}},{\rm{j}}$) to the neuron’s centroid, and ${\rm{L}}$ is oxygen diffusion length in tissue, which is set to 40 μm. This calculation is performed for each neuron, both before and after stimulation. The ratio change is used to quantify alterations in oxygen supply relative to neuronal spike responses.

## Supplementary information

Supplementary Information

Description of Additional Supplementary Files

Supplementary Movie 1

Supplementary Movie 2

Supplementary Movie 3

Supplementary Movie 4

Transparent Peer Review file

## Source data

Source Data

## Data Availability

The raw imaging data acquired in this study are available in the BioImage Archive (10.6019/S-BIAD3705). The source data underlying all graphs in this study are provided in the Source Data file. Source data are provided with this paper.
