# Supplementary material for: Integrated two-photon and photoacoustic microscopy for single-cell neurometabolic imaging
Source: Nat Commun. 2026 Jul 15;17:8670. doi: 10.1038/s41467-026-75603-7 (PMC13490390; doi:10.1038/s41467-026-75603-7)
Supplement: Supplementary file 1 [file 41467_2026_75603_MOESM1_ESM.pdf]

Supplementary Information

Integrated two-photon and photoacoustic microscopy for single-cell neurometabolic imaging

Jiaxiao Han[1], Youngseop Lee[2,3], Ziang Feng[1], Yue Wu[1], Zhuoying Wang[1], Allison Martinez Mejia[1], Adam Bauer[4], Manu Goyal[4], Jin-Moo Lee[5], Peinan Zhao[6], Hao F. Zhang[2], Cheng Sun[3], Song Hu[1,*]

[1]*Department of Biomedical Engineering, Washington University in St. Louis, St. Louis, MO 63130, USA*

[2]*Department of Biomedical Engineering, Northwestern University, Evanston, IL 60208, USA*

[3]*Department of Mechanical Engineering, Northwestern University, Evanston, IL 60208, USA*

[4]*Department of Radiology, Washington University in St. Louis, St. Louis, MO 63130, USA*

[5]*Department of Neurology, Washington University in St. Louis, St. Louis, MO 63130, USA*

[6]*Department of Obstetrics and Gynecology, Washington University in St. Louis, St. Louis, MO 63130, USA*

**Correspondence should be addressed to Dr. Song Hu (songhu@wustl.edu)*

Supplementary Note 1. Optimization of the optically transparent micro-ring resonator for ultrasound detection in integrated two-photon and photoacoustic microscopy.

Supplementary Fig. 1. Configuration of the micro-ring resonator.

Supplementary Fig. 2. Characterization of the MRR-based ultrasound sensor.

Supplementary Fig. 3. Schematic illustration of the method for quantifying the oxygen release rate of individual red blood cells.

Supplementary Fig. 4. Detailed schematic of the TPM-PAM system.

Supplementary Fig. 5. Resolutions of the TPM-PAM system.

Supplementary Fig. 6. Quantification of blood flow speed by kymographic analysis.

Supplementary Fig. 7. Neurometabolic responses of individual vessels to whisker stimulation in the awake GCaMP mouse brain.

Supplementary Fig. 8. Statistical analysis of the peak time of oxygen-metabolic responses to whisker stimulation.

Supplementary Fig. 9. Statistical analysis of the rise time of oxygen-metabolic responses to whisker stimulation.

Supplementary Fig. 10. Statistical analysis of the decay time of oxygen-metabolic responses to whisker stimulation.

Supplementary Fig. 11. System control and image acquisition.

Supplementary Fig. 12. Experimental setup for whisker stimulation in awake mice.

Supplementary Fig. 13. Kymographic scanning paths used in all imaging experiments.

Supplementary Fig. 14. Flow chart of data processing for extracting individual RBC clusters.

Supplementary Fig. 15. Time gating to remove crosstalk of PAM laser on TPM imaging.

Supplementary Table 1. Scanning parameters used in all imaging experiments.

Supplementary Note 1. Optimization of the optically transparent micro-ring resonator for ultrasound detection in integrated two-photon and photoacoustic microscopy.

We developed a polymer-based micro-ring resonator (MRR) for high-sensitivity ultrasound detection while preserving full optical access for fluorescence excitation and collection in the integrated two-photon and photoacoustic microscopy (TPM-PAM) system. The MRR was fabricated using soft nanoimprint lithography (sNIL) based on our previous work[1,2] and then packaged with two optical fibers on a glass carrier substrate (Supplementary Fig. 1a).

Briefly, a primary silicon (Si) master template was first fabricated using electron beam lithography, followed by reactive ion etching. A polydimethylsiloxane (PDMS) soft mold was then replicated from the Si master template. Using the PDMS mold, we imprinted the MRR onto a spin-on-glass (SOG) dielectric material-coated borosilicate substrate via sNIL, with polystyrene as the constructing material. The fabricated MRR has a radius of 40 μm and a cross-section of 850 nm × 850 nm, ensuring single-mode operation (Supplementary Fig. 1b-c). A 6-μm-thick polymer layer (MY Polymer Ltd. MY-131-MC; refractive index, ~1.31) was spin-coated on top of the MRR as the upper cladding, which also prevented surface contamination from biological tissues (Supplementary Fig. 1d). Finally, the MRR was packaged with a single-mode fiber (SMF; Thorlabs S630-HP) and a multi-mode fiber (MMF; Thorlabs GIF625) on the transparent glass substrate. The SMF and MMF were aligned to the input and output waveguides, respectively, to couple wavelength-tunable continuous-wave (CW) light into the MRR and to connect with a photodetector for monitoring resonance shifts induced by ultrasonic waves.

To enhance the ultrasound detection sensitivity and thus the signal-to-noise ratio (SNR) of PAM in the integrated system for functional measurements at single-red-blood-cell (RBC) resolution, we optimized the coupling efficiency and quality factor (Q-factor) of the MRR through two new strategies. Here, coupling refers to the ratio of the optical power at the output multimode fiber to that at the input single-mode fiber, accounting for coupling losses at both the input and output ports of the MRR waveguide. Because the coupling loss from the MRR waveguide to the multimode fiber (with a large collection numerical aperture of 0.4) is significantly smaller than that from the single-mode fiber to the MRR waveguide, the overall coupling loss is predominantly from the input port due to the mode mismatch. Accordingly, the coupling improvement in this work is primarily focused on reducing the input coupling loss.

First, in our previous MRR design[1], the SMF with a core diameter of 3.5 μm (corresponding to a mode field diameter of ~5.0 μm at 780 nm) was directly coupled to a straight bus waveguide (straight WG) with a cross-section of 850 nm × 850 nm. The large mode mismatch between the SMF and straight WG resulted in a low coupling efficiency of less than 5%, significantly limiting ultrasound sensitivity and SNR. To mitigate the mode mismatch, we introduced adiabatically tapered waveguides (Tapered WGs) for mode conversion. The tapered WG was 3-μm wide at the entrance and gradually narrowed to 850 nm to match the straight WG. Finite-element simulation (COMSOL Inc. COMSOL Multiphysics 6.0) of the fundamental mode profiles in the SMF, straight WG, and tapered WG at 780 nm confirmed that this new design can effectively reduce the mode mismatch. Experimentally, the coupling efficiency increased from 5% to 19%.

Second, we improved the MRR's Q-factor in the presence of the protection layer by optimizing both the cladding and substrate materials. This optimization minimized optical propagation loss within the micro-ring waveguide and enhanced acoustic impedance matching between water and the waveguide material. Specifically, we changed the top cladding from PDMS (refractive index: ~1.40) to an optical acrylic resin with a low refractive index (~1.31) and the bottom cladding from quartz (~1.45) to a polysiloxane-based SOG dielectric material (~1.43).

The ultrasound detection sensitivity of the MRR is closely related to the Q-factor of the whispering-gallery-mode resonance, which is governed by optical energy loss in the resonator. While PDMS is widely used as an elastomeric material, it is not optimized for low-loss optical applications. As a result, the optical loss associated with PDMS cladding limits the achievable Q-factor of the MRR. To mitigate this limitation, we replaced PDMS with a low refractive index optical acrylic resin (MY-131-MC) that was developed for optical coating applications. Although detailed optical loss parameters are not available, such materials are generally associated with reduced absorption compared with elastomeric polymers and provide improved optical confinement due to increased refractive index contrast relative to the polystyrene core (~1.58). Together, these factors contribute to reduced cavity loss in the ring resonator. Consistent with this expectation, MRRs employing the low-index optical cladding exhibit substantially higher Q-factors than those using PDMS cladding, with average Q factors of 1.7×10^5 and 3.4×10^4 for MY-131-MC and PDMS, respectively. In addition, the improved mode confinement reduces evanescent field leakage, making the MRR less susceptible to environmental perturbations and enhancing device reproducibility under *in vivo* imaging conditions.

In addition to optical considerations, acoustic impedance mismatch was also considered. Acoustic impedance (Z) is defined as $Z = \rho \cdot v$, where ρ is material's density and v is acoustic velocity within the material. Standard PDMS (e.g., Sylgard 184) has an acoustic impedance of ~1.1 MRayl[3], which differs significantly from both water (Z ~1.5 MRayl)[4] and the polystyrene MRR (Z ~2.5 MRayl)[5]. Although the acoustic impedance of MY-131-MC is not explicitly reported, it can be estimated from its density (~1.74 g/cm^3)[6], Young's Modulus (~2 MPa)[7], and typical longitudinal sound velocities of soft elastomer or fluorinated polymers[3,8], yielding an expected Z range of ~1.7–2.3 MRayl. This range is closer to that of polystyrene, improving impedance matching at the cladding-resonator interface and reducing internal acoustic reflections, while maintaining only a moderate mismatch with water.

Furthermore, employing the SOG material as the bottom cladding enabled the use of borosilicate glass as the substrate instead of quartz, providing more stable handling during the process of dicing the MRR substrate into proper-sized pieces. The amorphous structure and lower hardness of the borosilicate glass allow cracks to propagate uniformly, enabling controlled and clean dicing with minimal chipping.

In summary, innovations in the waveguide design and material selection enhanced the MRR's coupling efficiency by 3.8× and the Q-factor by 5.0×, collectively resulting in more than one order of magnitude increase in the ultrasound sensitivity and SNR. This new generation of MRR with substantially boosted SNR enables TPM-PAM to perform functional measurements at the single-RBC level.

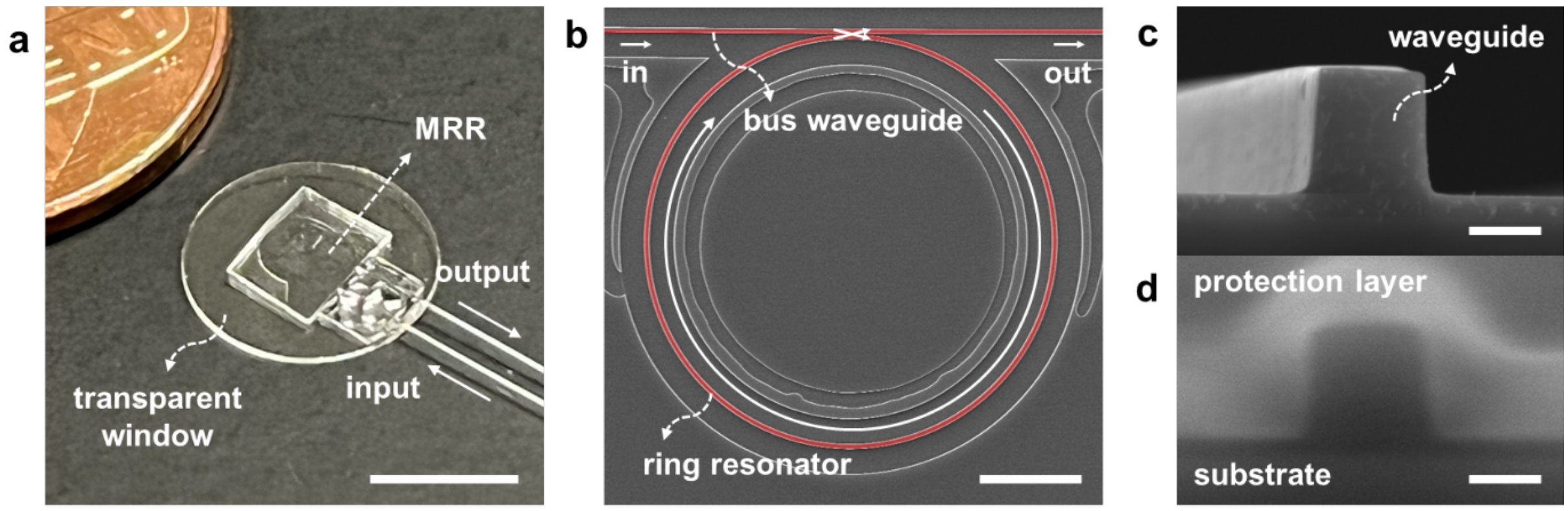

Supplementary Fig. 1 | Configuration of the micro-ring resonator. a, Photograph of the packaged MRR with a single-mode fiber and a multi-mode fiber on a glass coverslip. **b,** Scanning electron microscopy (SEM) image of the MRR. **c,** Cross-sectional SEM image of the MRR without the protection layer. **d,** Cross-sectional SEM image of the MRR with the protection layer. Scale bars: **a,** 5 mm; **b,** 20 μm; **c, d,** 500 nm.

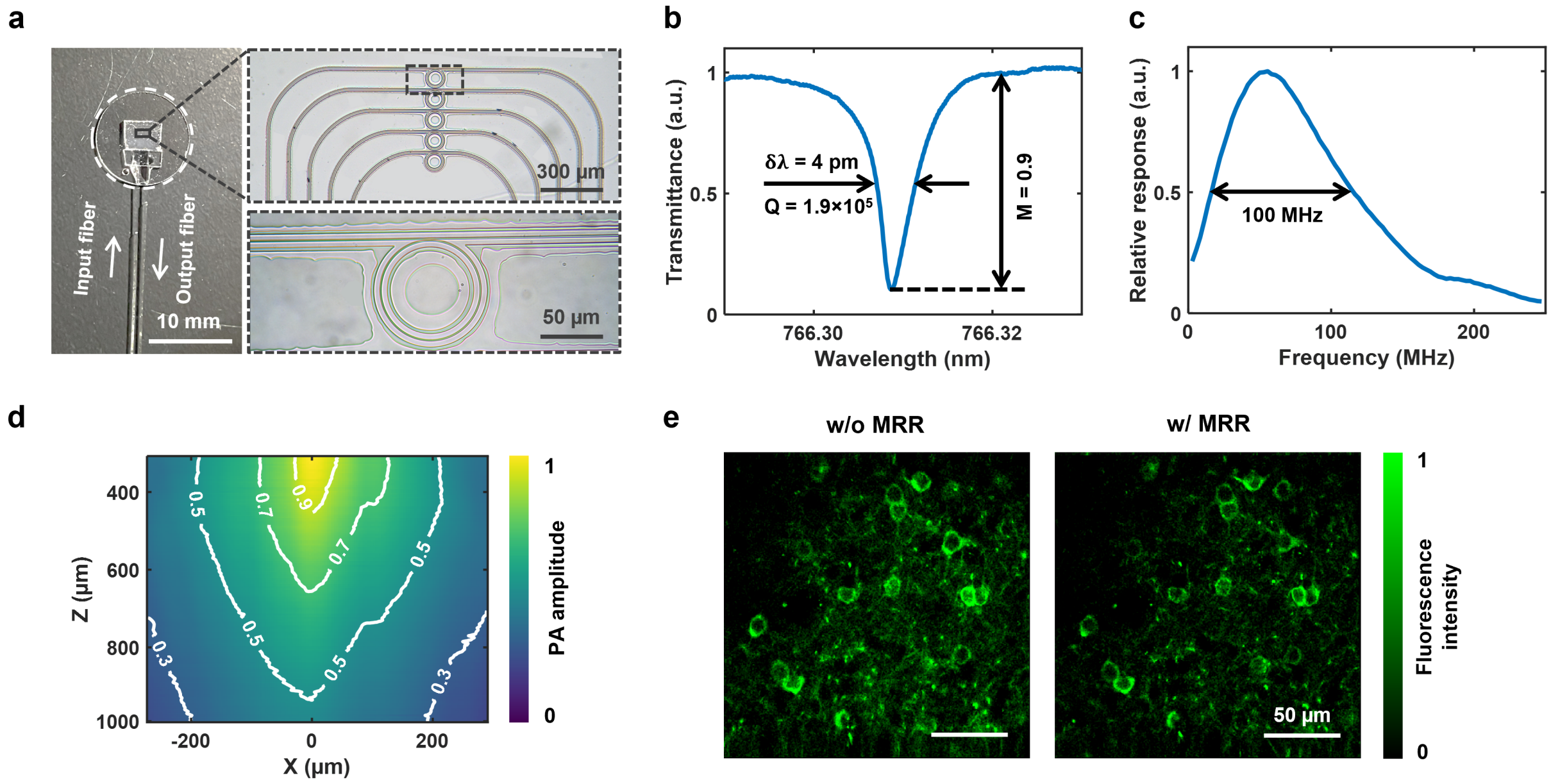

Supplementary Fig. 2 | Characterization of the MRR-based ultrasound sensor. a, Photograph of a packaged polymer MRR (left) and optical micrographs showing five pairs of micro-rings (diameter 80 μm) and matching bus waveguides fabricated via soft nanoimprint lithography (right). The pair with optimal Q-factor and modulation depth is selected for photoacoustic detection, with the input and output fibers precisely aligned and fixed to the two end facets of the waveguides for light butt-coupling. **b,** Transmission spectrum of the MRR around a resonant wavelength, exhibiting a Q-factor of 1.9×10^5 and a modulation depth (M) of 0.9. **c,** Frequency response of the MRR, derived from the Fourier transform of the photoacoustic A-line signals generated by an ultra-thin metal layer, shows a 3-dB bandwidth of 100 MHz. **d,** Sensitivity map of the MRR in the lateral (X) and axial (Z) directions. The Z position represents the distance between the MRR and the imaging target plane (black tape in this case). **e,** *In vivo* TPM images of neurons acquired without (left) and with (right) the MRR placed beneath the microscope objective. All imaging parameters, including laser power and field of view, were kept the same. The high optical transparency of the MRR resulted in negligible optical aberration and only a modest 20% reduction in detected fluorescence intensity.

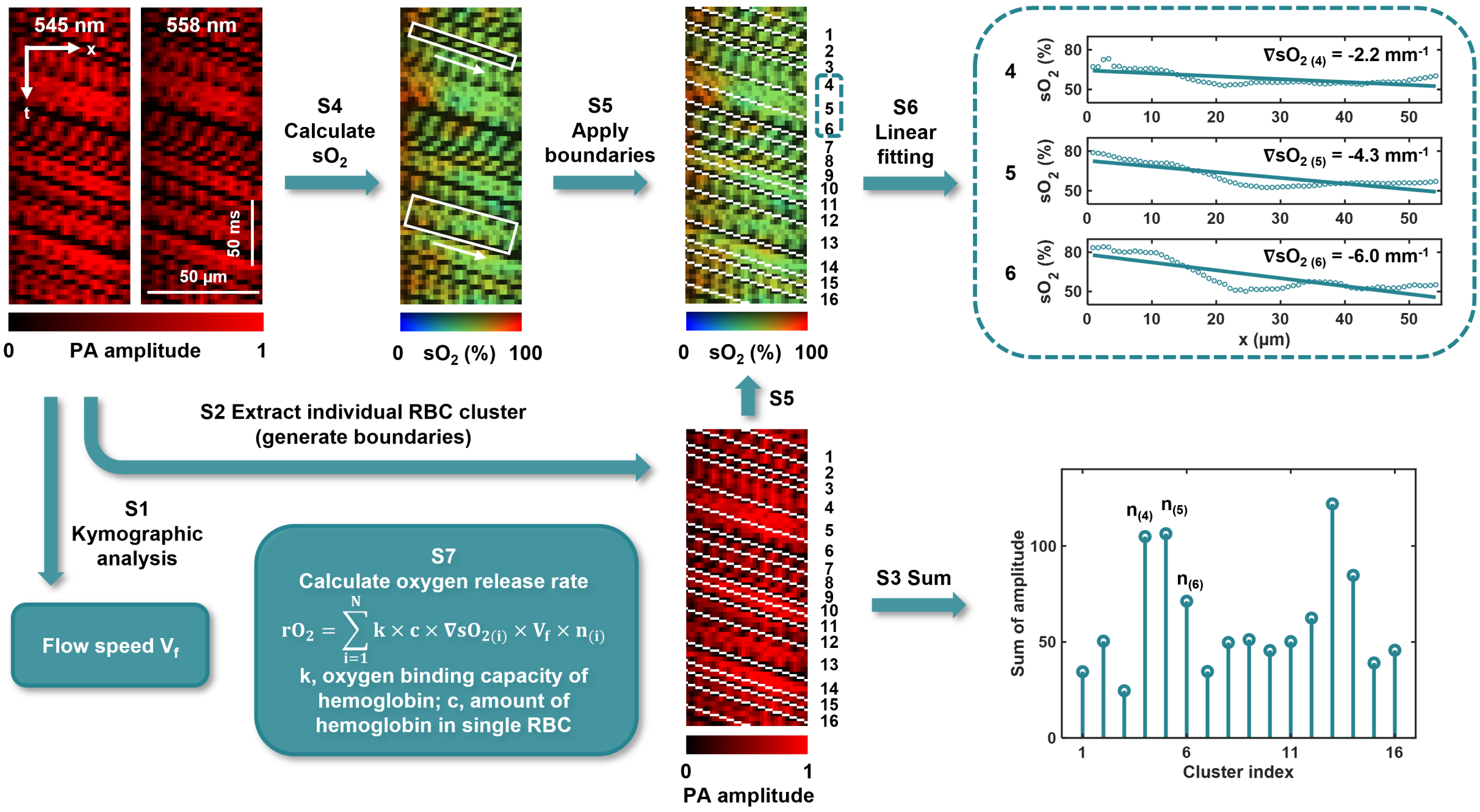

$$rO_2 = \sum_{i=1}^{N} k \times c \times \nabla sO_{2(i)} \times V_f \times n_{(i)}$$

Supplementary Fig. 3 | Schematic illustration of the method for quantifying the oxygen release rate of individual red blood cells. Detailed description of the computational method is provided in the Methods.

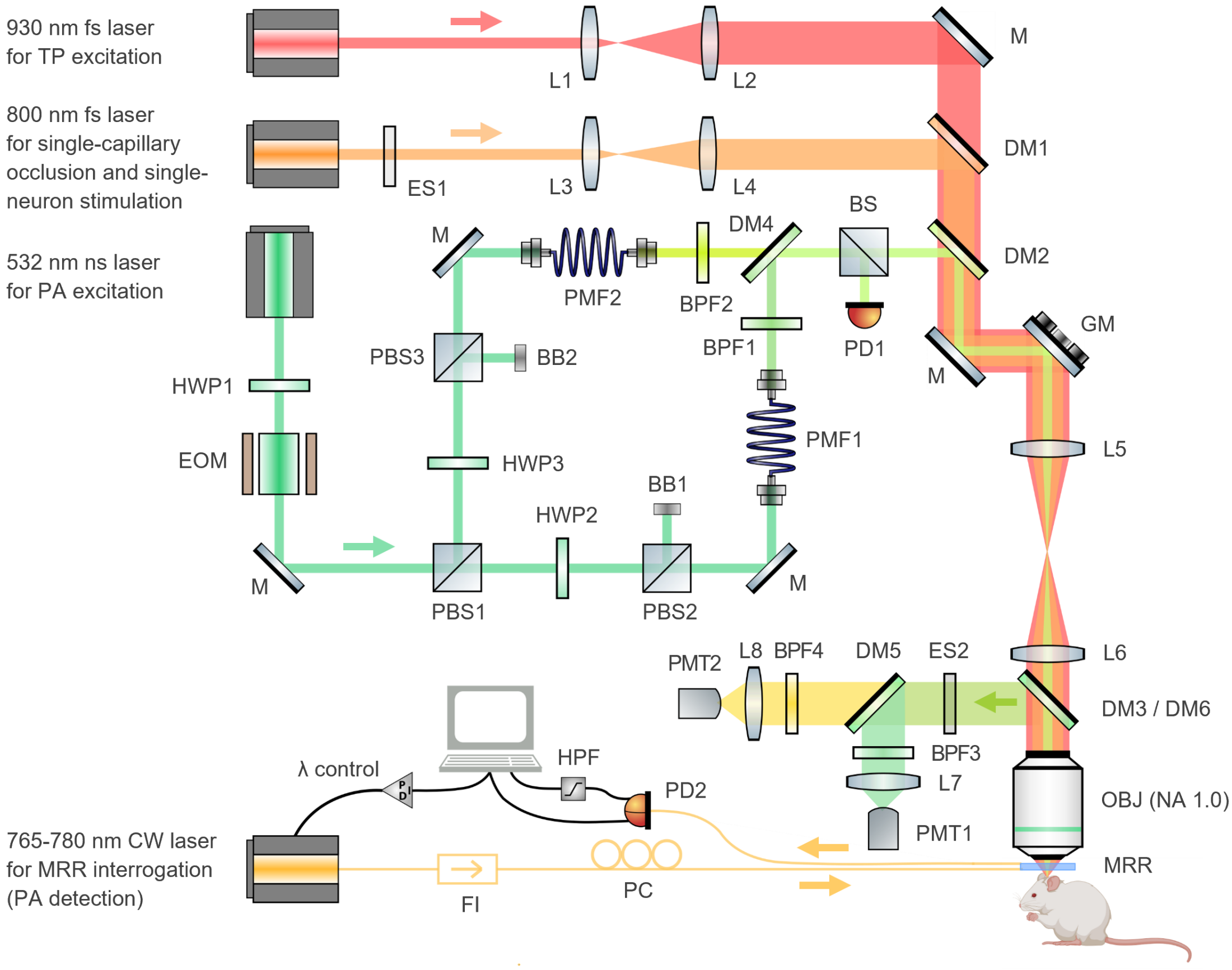

Supplementary Fig. 4 | Detailed schematic of the TPM-PAM system. fs, femtosecond; ns, nanosecond; CW, continuous wave; L, lens; M, mirror; DM, dichroic mirror; GM, galvanometer; HWP, half-wave plate; EOM, electro-optic modulator; PBS, polarizing beamsplitter; BB, beam block; PMF, polarization maintaining fiber; BPF, optical bandpass filter; BS, beam sampler; PD, photodetector; OBJ, objective; MRR, micro-ring resonator; ES, electrical shutter; PMT, photomultiplier tube; BPD, balanced photodetector; HPF, electrical high-pass filter; FI, fiber isolator; PC, polarization controller; PID, proportional-integral-derivative controller; λ, wavelength. Detailed description of the system design is provided in the Methods. This figure contains elements created in BioRender. Han, J. (2026) https://BioRender.com/11f3rr8.

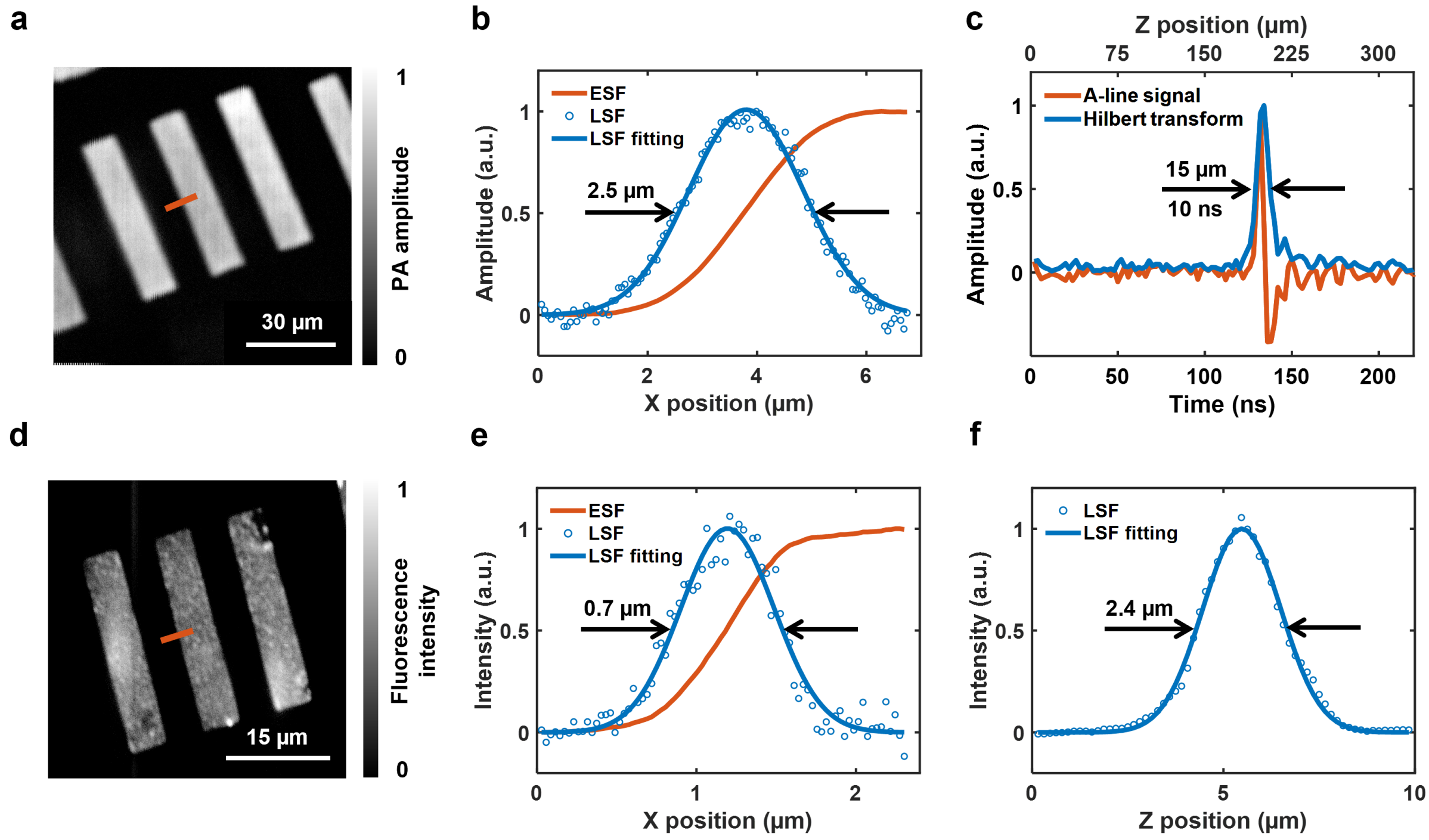

Supplementary Fig. 5 | Resolutions of the TPM-PAM system. a, Photoacoustic (PA) image of a bar pattern on an absorption resolution target. **b,** Edge spread function (ESF) extracted from the edge of the bar pattern (orange line in **a**). Line spread function (LSF) was derived by taking the derivative of the ESF. PAM's lateral resolution was estimated to be 2.5 µm based on the full-width-half-maximum (FWHM) value of the Gaussian fit to the LSF. **c,** Photoacoustic A-line signal generated by an ultra-thin metal layer. PAM's axial resolution was estimated to be 15 µm based on the FWHM value of the Hilbert-transformed signal. **d,** TPM image of a bar pattern on a fluorescent resolution target. **e,** ESF across the edge of the bar pattern (orange line in **d**) and the derived LSF. TPM's lateral resolution was estimated to be 0.7 µm. **f,** Intensity profile of a 0.5-µm-diameter fluorescent bead scanned along the axial (Z) axis. TPM's axial resolution was estimated to be 2.4 µm.

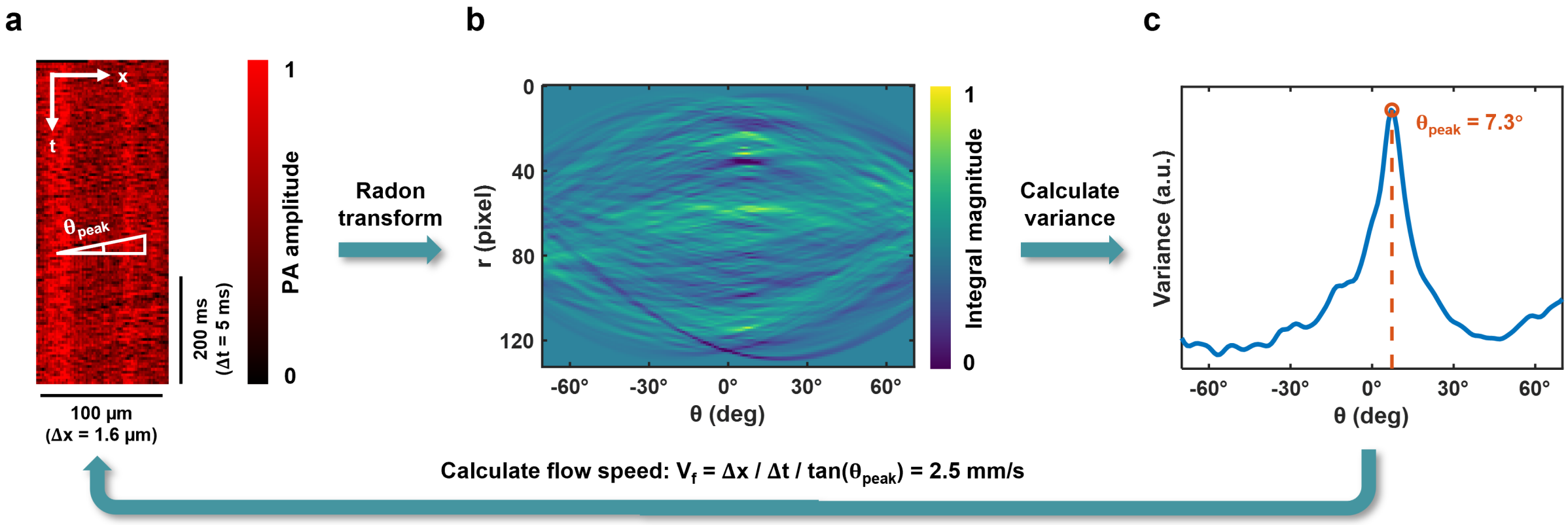

Supplementary Fig. 6 | Quantification of blood flow speed by kymographic analysis. a, Representative flow kymograph acquired by TPM-PAM from a microvessel in the mouse brain *in vivo*. The pixel size along the x and t axes are 1.6 μm and 5 ms, respectively. **b,** Radon transform of the flow kymograph. **c,** Variance at different angles derived from Radon transform results. The angle corresponding to the peak variance (θ_{peak}) is used to quantify blood flow speed through $V_f = \Delta x / \Delta t / \tan(\theta_{peak})$.

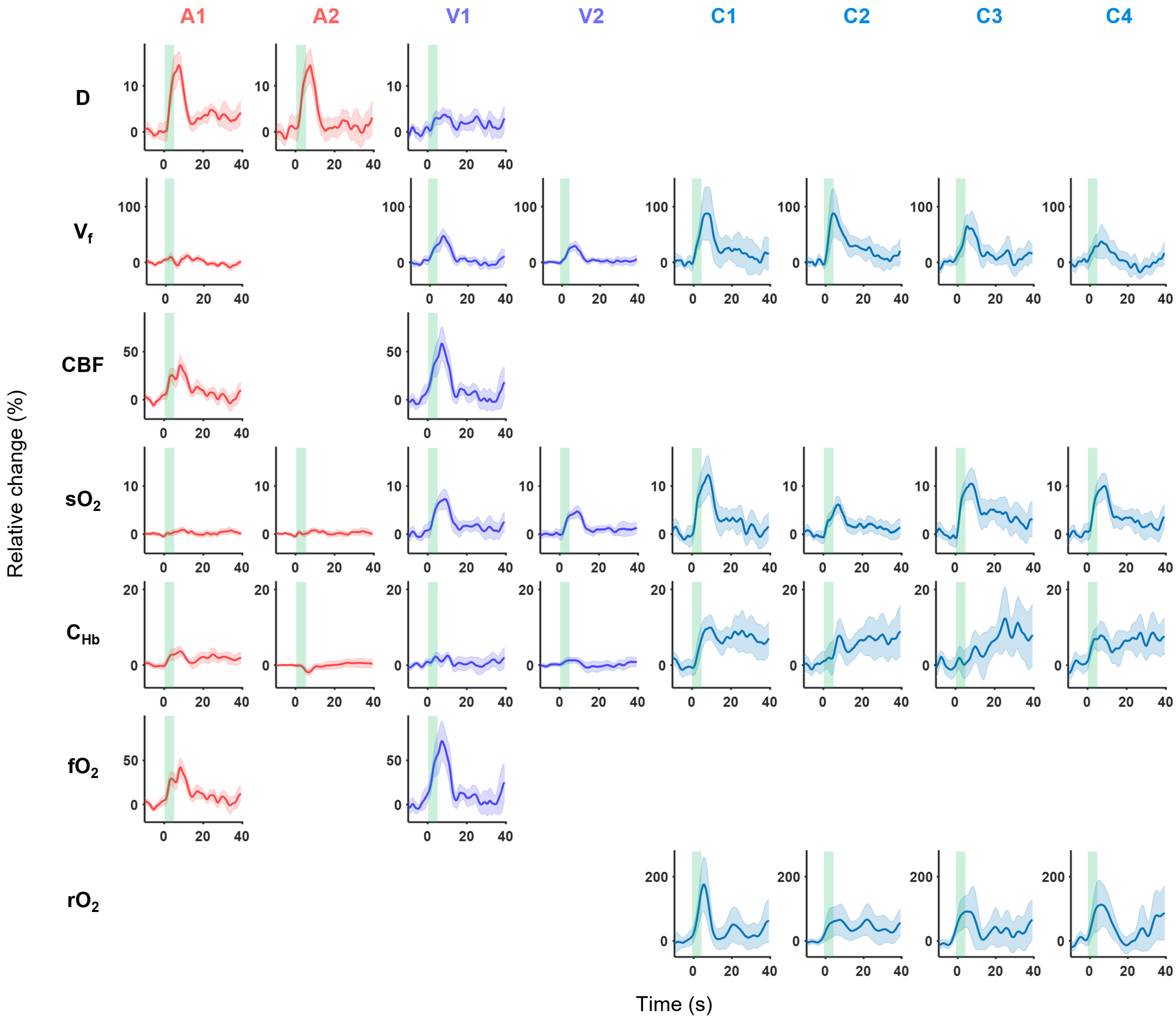

Supplementary Fig. 7 | Neurometabolic responses of individual vessels to whisker stimulation in the awake GCaMP mouse brain. All responses were averaged over ten stimulation trials and plotted in relative changes. Green shadows indicate the stimulation period. The diameter of vessel V2 could not be measured because it was located near the edge of the field of view. Capillary diameters were also not measured because intermittent passage of red blood cells prevented reliable quantification. Consequently, capillary CBF and fO_2, both dependent on D, were not quantified. The V_f in arteriole A2 exceeded the maximal speed that the kymograph could resolve, which was limited by the scanning speed of galvanometer mirror. The rO_2 was quantified only for capillaries, where oxygen release primarily occurs.

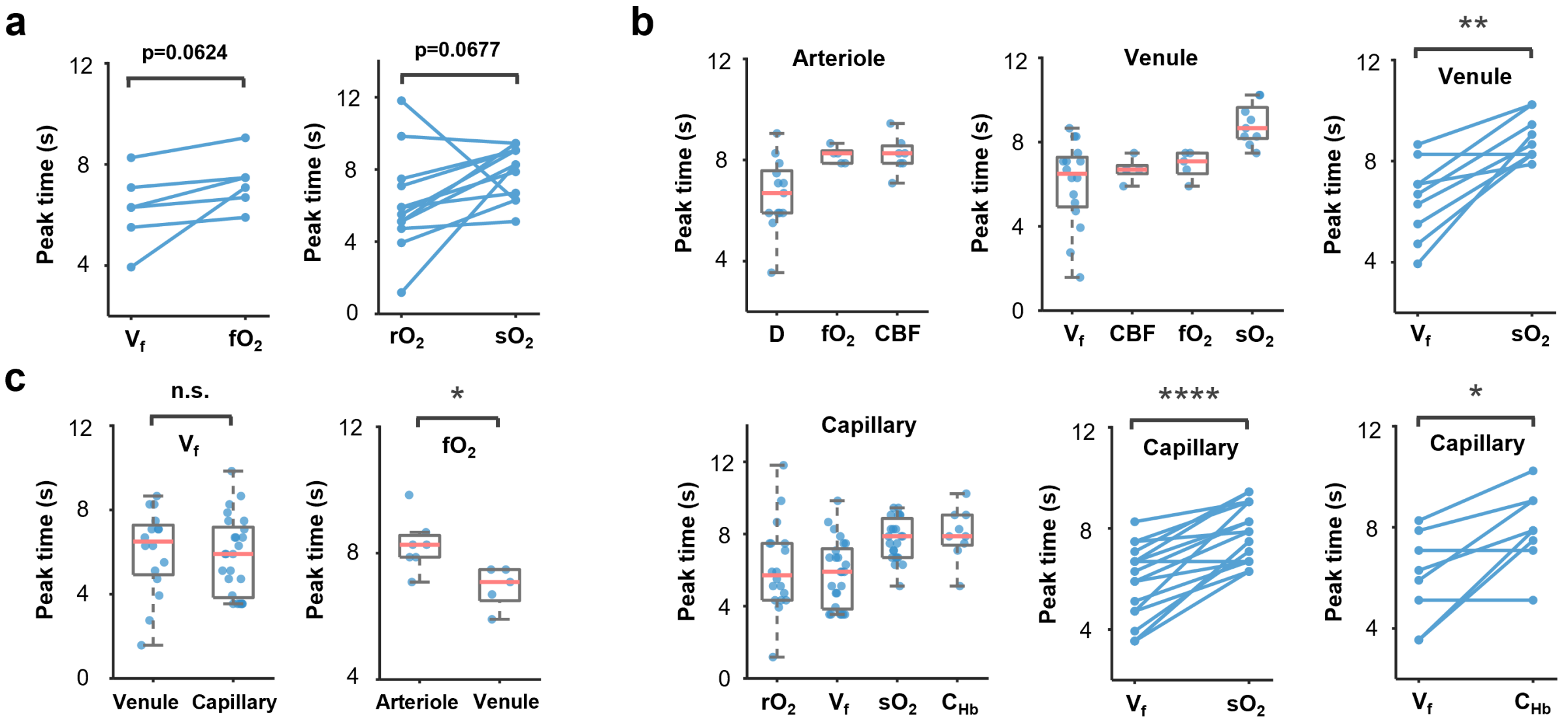

Supplementary Fig. 8 | Statistical analysis of the peak time of oxygen-metabolic responses to whisker stimulation. Peak time is defined as the interval between the stimulus onset to the maximum amplitude of the response. Fig. 2e shows the peak times of the eight parameters measured across all vessels over the ten trials, ranked in an ascending order based on their median values. **a,** Paired *t*-tests were performed for all pairwise combinations of parameters. Four most informative comparisons are shown here and in Fig. 2f, indicating that the response of V_f peaked significantly earlier than those of sO_2 and C_{Hb} (**** $p < 0.0001$, *** $0.0001 \leq p < 0.001$, ** $0.001 \leq p < 0.01$, * $0.01 \leq p < 0.05$). **b,** Peak times of all responsive parameters categorized by the vessel type, with corresponding paired *t*-tests. The results show that V_f peaked earlier than sO_2 in both venules and capillaries. Moreover, V_f peaked earlier than C_{Hb} in capillaries. **c,** Unpaired *t*-tests between different vessel types for the same parameter are displayed here and in Fig. 2g, revealing that sO_2 peaked earlier in capillaries than in venules, whereas CBF and fO_2 responses peaked earlier in venules than in arterioles.

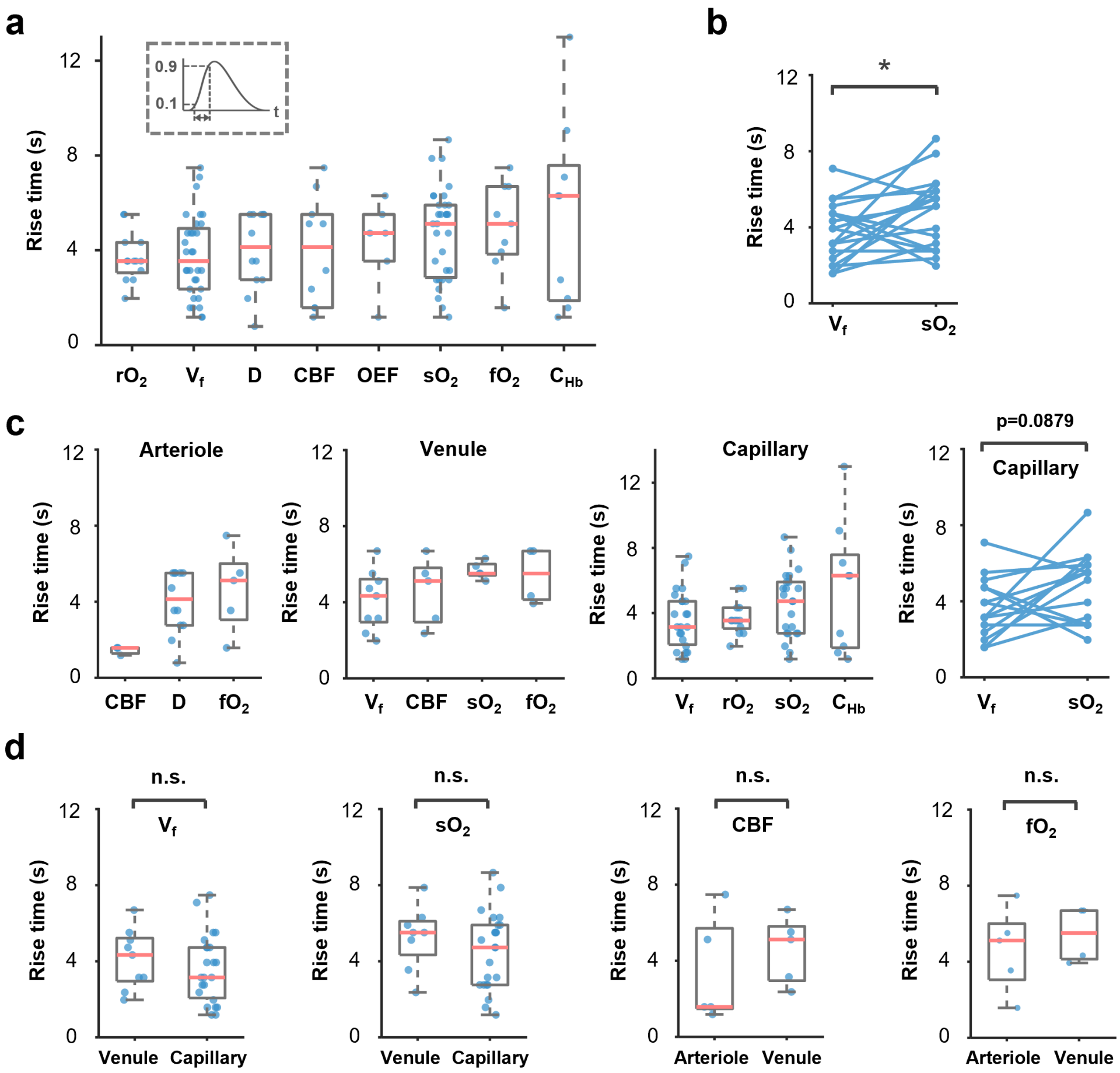

Supplementary Fig. 9 | Statistical analysis of the rise time of oxygen-metabolic responses to whisker stimulation. Rise time is defined as the interval between 10% to 90% of the maximum response amplitude measured on the rising edge. **a,** Rise times of the eight parameters measured across all vessels over the ten trials, ranked in an ascending order based on their median values. **b,** Paired *t*-tests were performed for all pairwise combinations of parameters. The only significant comparison is shown, indicating that the response of V_f rose faster than sO_2 (* $0.01 \le p < 0.05$). **c,** Rise times of all responsive parameters categorized by the vessel type, with the paired *t*-test between V_f and sO_2 in capillaries. **d,** Unpaired *t*-tests between different vessel types for the same parameter reveal no significant inter-vessel differences.

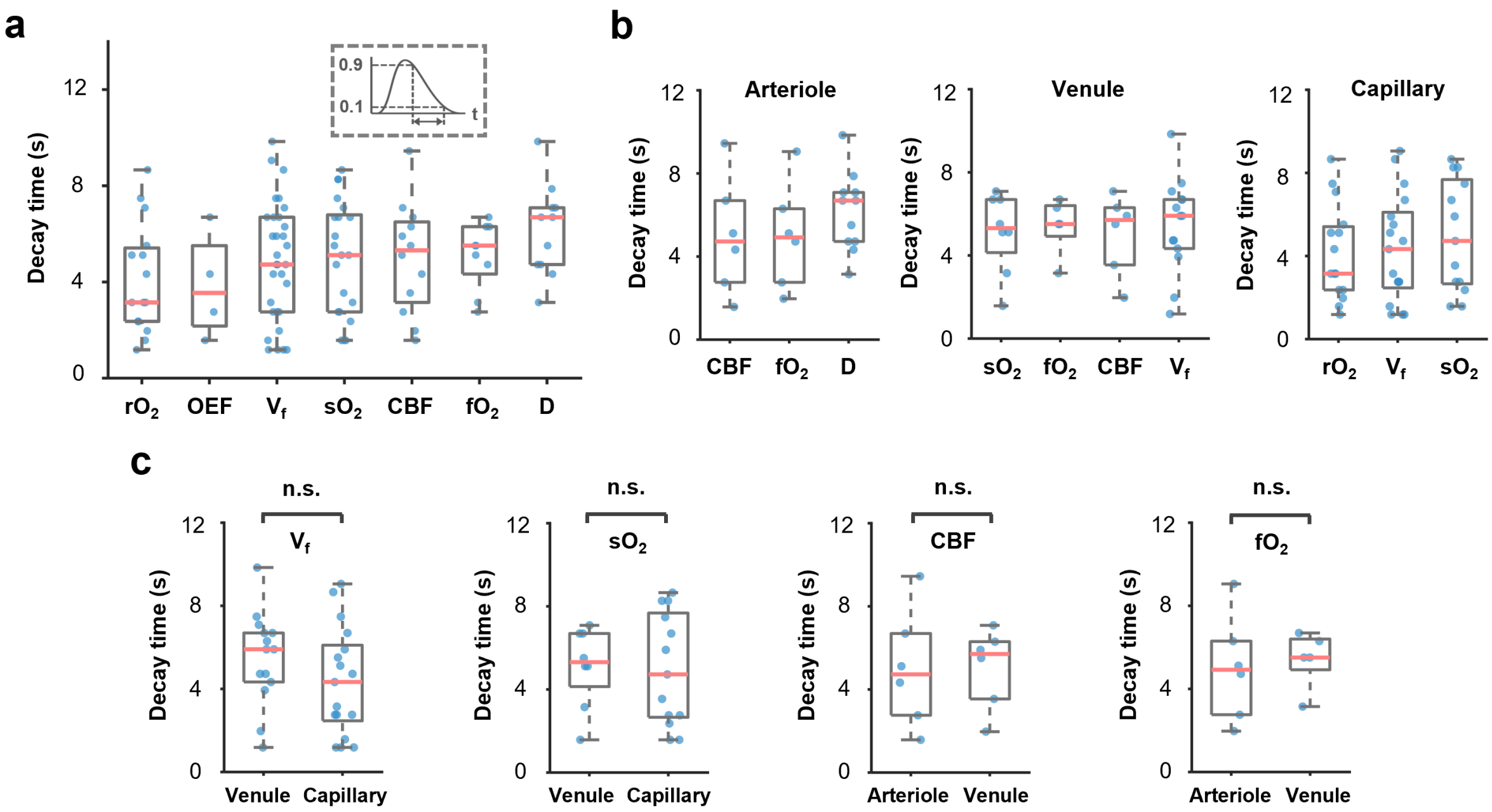

Supplementary Fig. 10 | Statistical analysis of the decay time of oxygen-metabolic responses to whisker stimulation. Decay time is defined as the interval between 90% to 10% of the maximum response amplitude measured on the falling edge. **a,** Decay times of the seven parameters measured across all vessels over the ten trials, ranked in an ascending order based on their median values. The decay time of C_{Hb} was not quantified because its response in capillaries did not return to the baseline within the imaging period after stimulation. Paired *t*-tests were performed for all pairwise combinations of parameters, and no significant differences were observed. **b,** Decay times of all responsive parameters categorized by the vessel type. **c,** Unpaired *t*-tests between different vessel types for the same parameter reveal no significant inter-vessel differences.

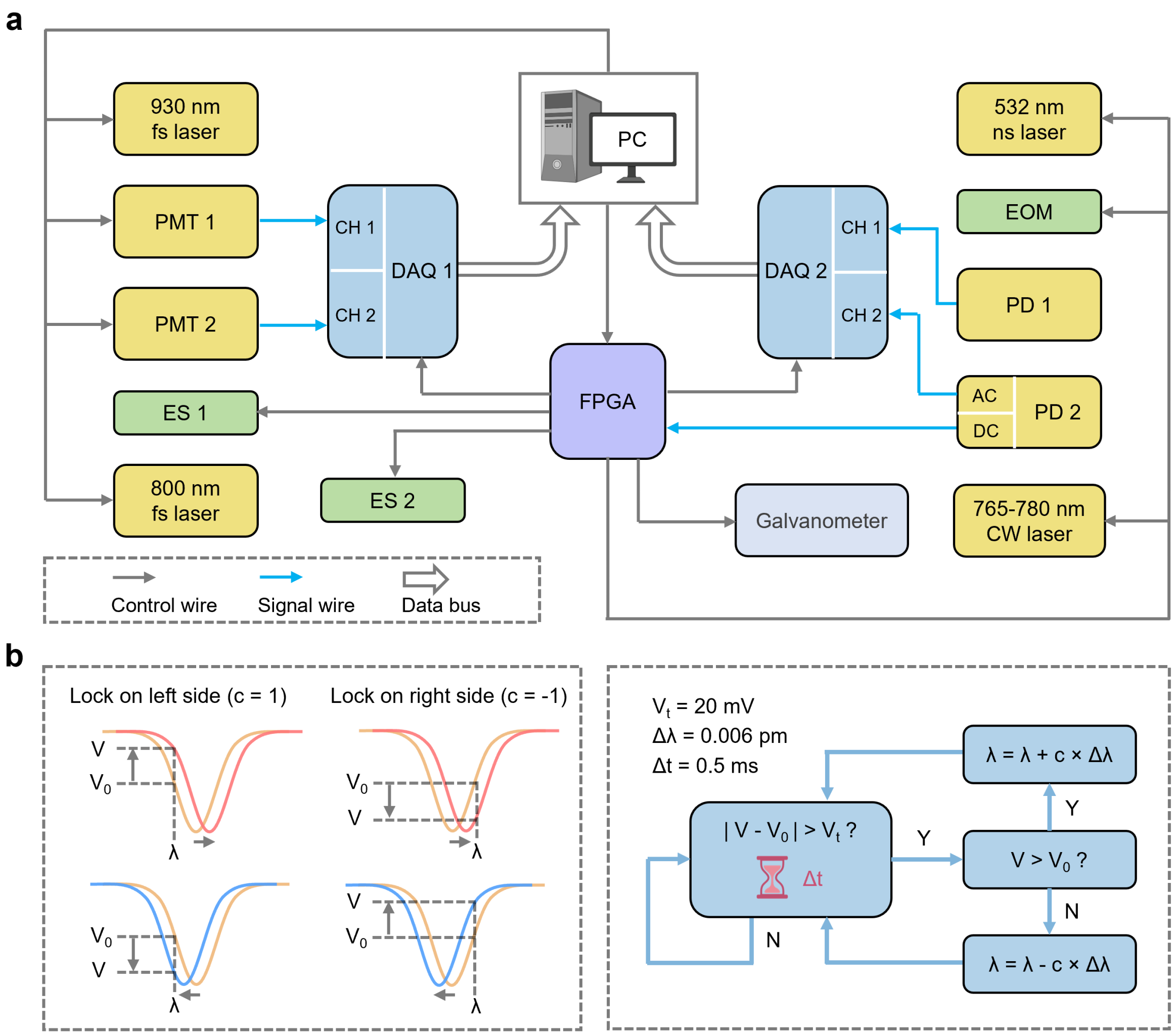

Supplementary Fig. 11 | System control and image acquisition. a, Block diagram of the TPM-PAM platform. This system enables real-time, synchronized dual-modal imaging by incorporating an FPGA for hardware synchronization, a dual-channel DAQ 1 to digitize the TPM signals from two PMTs, a dual-channel DAQ 2 to compensate for fluctuations in laser pulse energy recorded by a PD 1 and to digitize the PAM signal from the AC channel of PD 2, and a dual-axis galvanometer mirror for laser scanning. **b,** Real-time compensation for thermal drifts of the micro-ring resonator (MRR). Left: four different thermal-drift conditions (i.e., red-shift vs. blue-shift, and locking on the left vs. right side of resonance dip). Right: schematic of the compensation strategy. The DC output of PD 2 is monitored by the FPGA every 0.5 ms. Once it deviates from the preset value by more than the threshold V_t, the FPGA tunes the wavelength (λ) of the CW laser via a PZT control by a small step Δλ to track the MRR's resonance drift. This real-time feedback control effectively eliminates the impact of thermal drift on imaging stability. fs, femtosecond; ns, nanosecond; PMT, photomultiplier tube; ES, electrical shutter; DAQ, data-acquisition card; CH, channel; FPGA, field programmable gate array; EOM, electro-optic modulator; PD, photodetector.

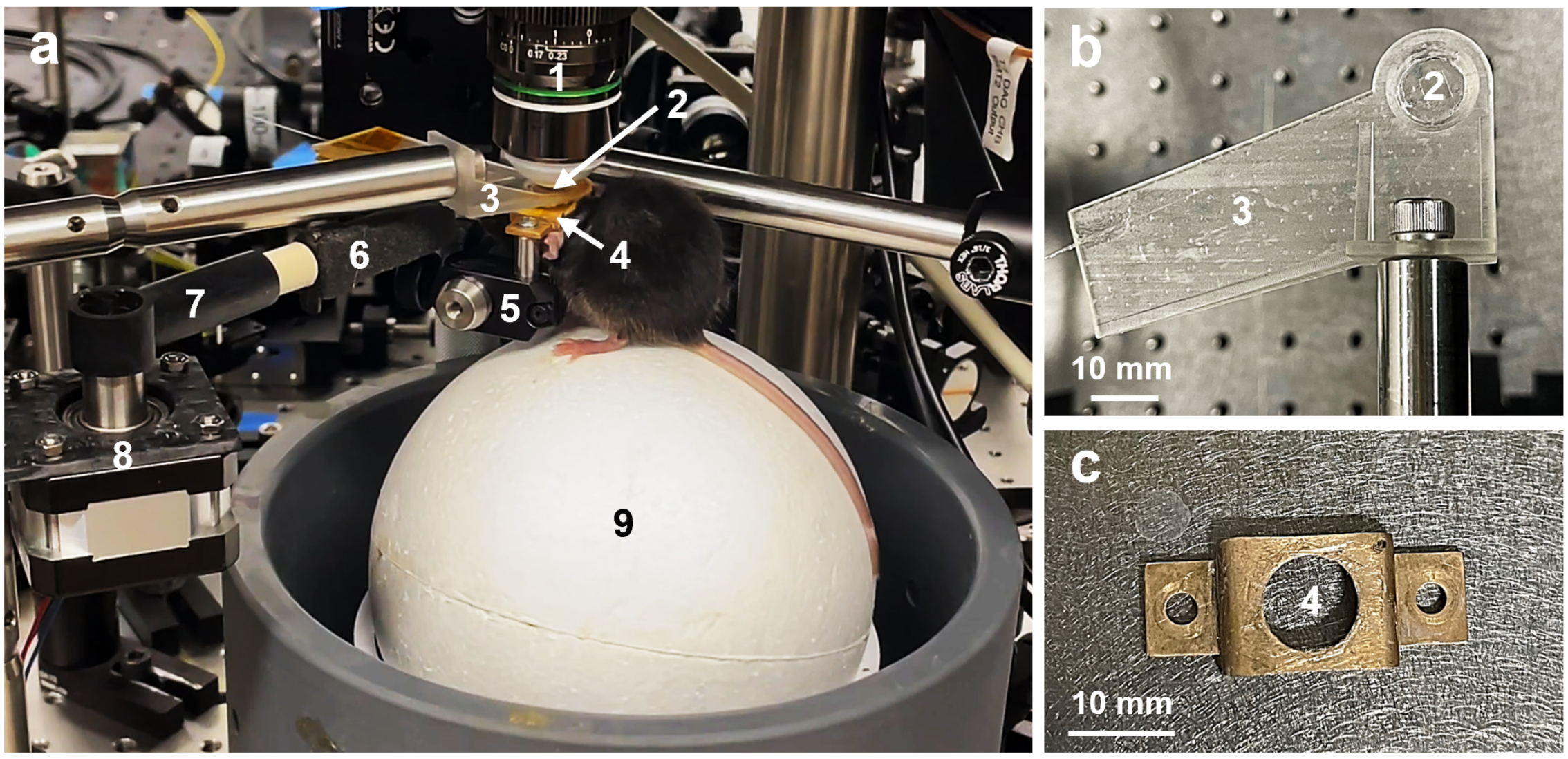

Supplementary Fig. 12 | Experimental setup for whisker stimulation in awake mice. a, Photograph of the complete setup. The mouse runs freely on an air-floated treadmill with its head fixed in position by a custom head frame. A foam brush, driven by a rotation motor, is used to deflect the whiskers. 1, objective; 2, MRR; 3, MRR holder; 4, head frame; 5, head frame holder; 6, foam brush; 7, foam brush adapter; 8, rotation motor; 9, air-floated treadmill. **b,** Photograph of the 3D-printed MRR holder. **c,** Photograph of the 3D-printed head frame.

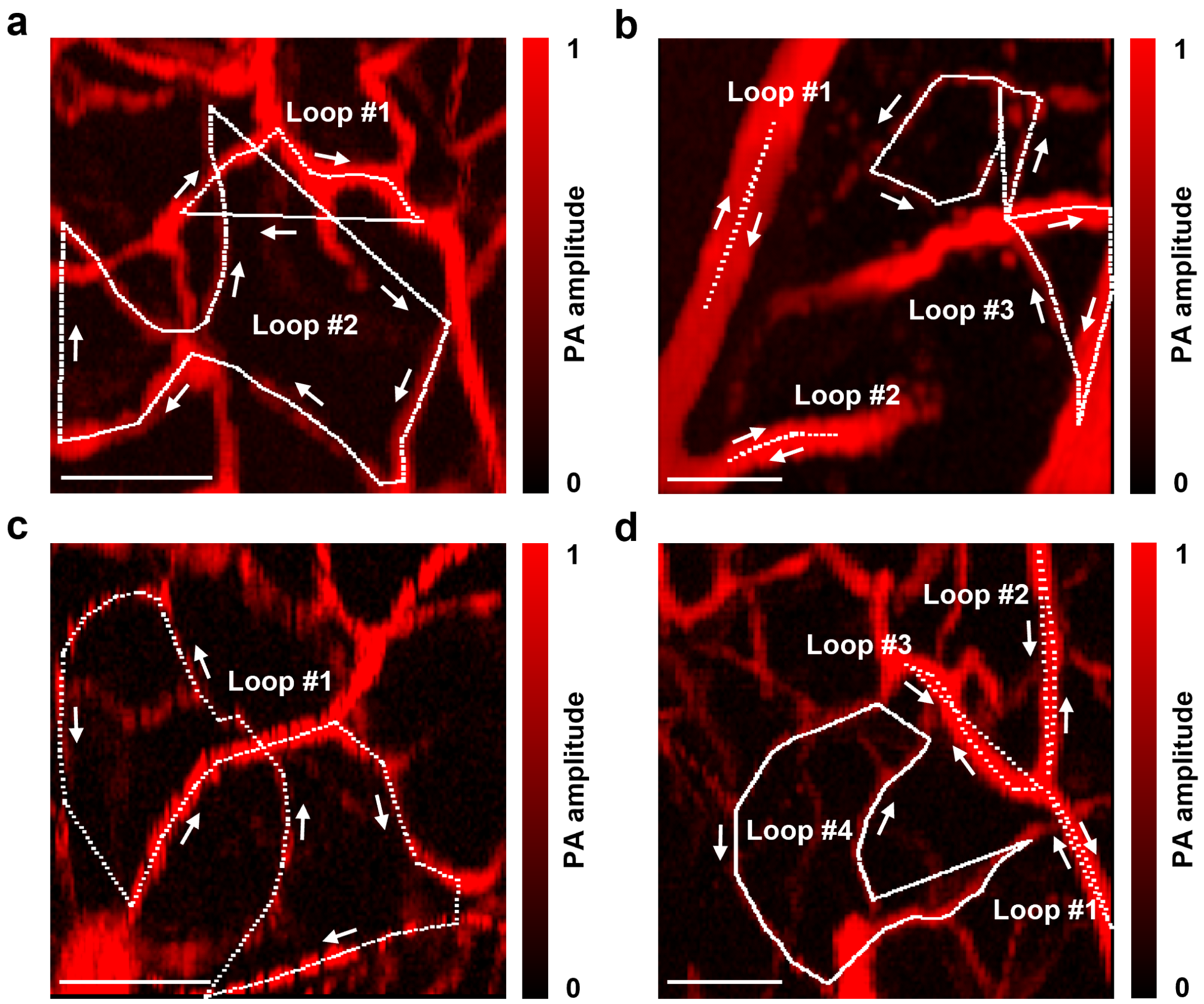

Supplementary Fig. 13 | Kymographic scanning paths used in all imaging experiments. a, Scanning path used in Fig. 1f-k. **b,** Scanning path used in the whisker stimulation experiment (Fig. 2). **c,** Scanning path used in the single-capillary occlusion experiment (Fig. 3). **d,** Scanning path used in the single-neuron stimulation experiment (Fig. 4). White arrows indicate scanning directions. Scale bar: 50 μm.

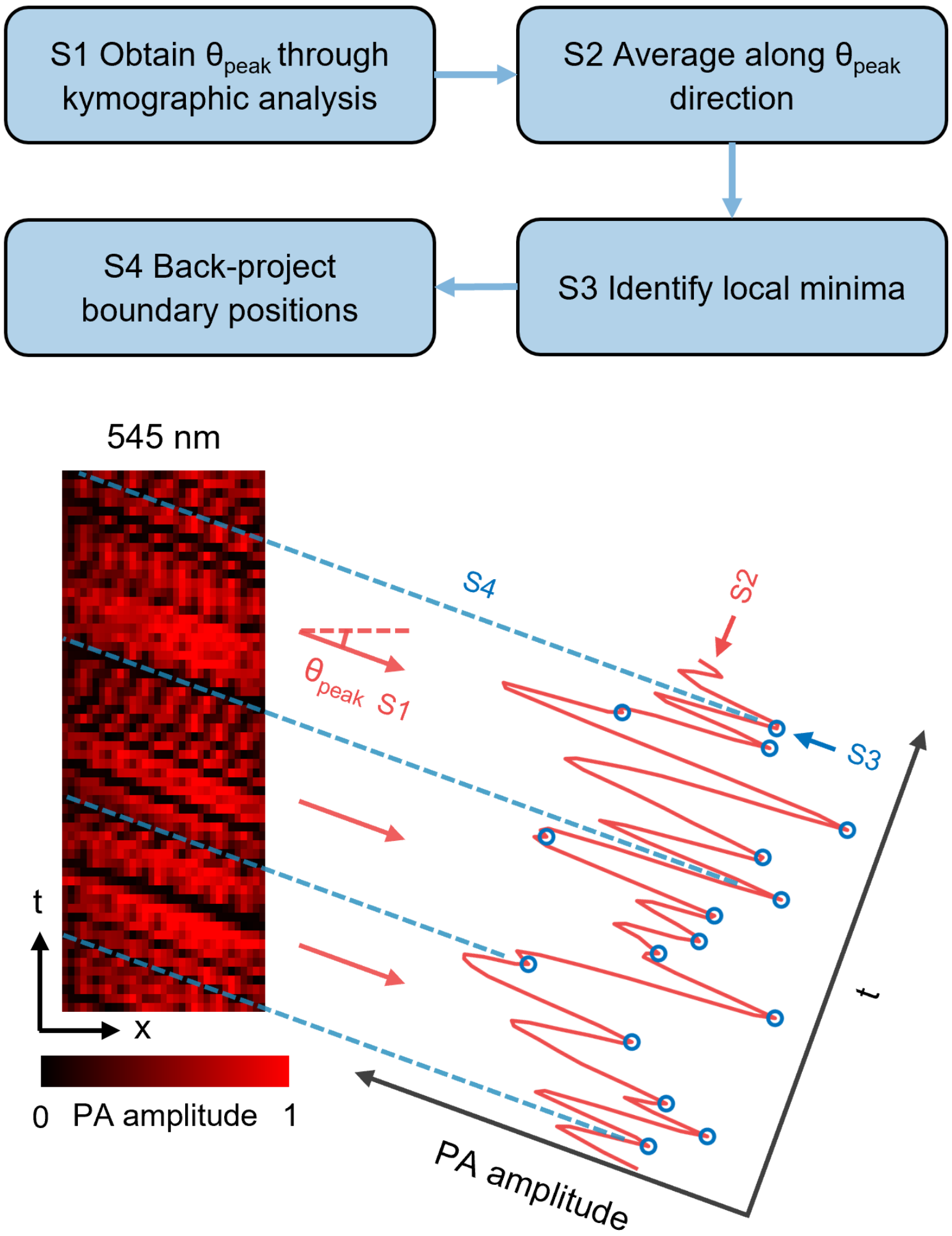

Supplementary Fig. 14 | Flow chart of data processing for extracting individual RBC clusters. S1, Apply the Radon transform to the kymograph acquired at 545 nm to determine θ_{peak} (Supplementary Fig. 6). S2, Average the photoacoustic amplitude along the θ_{peak} direction to obtain the red curve. S3, Identify all local minima, labeled by blue circles, which correspond to boundary positions of RBC clusters. S4, Back-project these boundary positions onto the kymograph to delineate individual clusters. Scale bars, x = 20 μm, t = 40 ms.

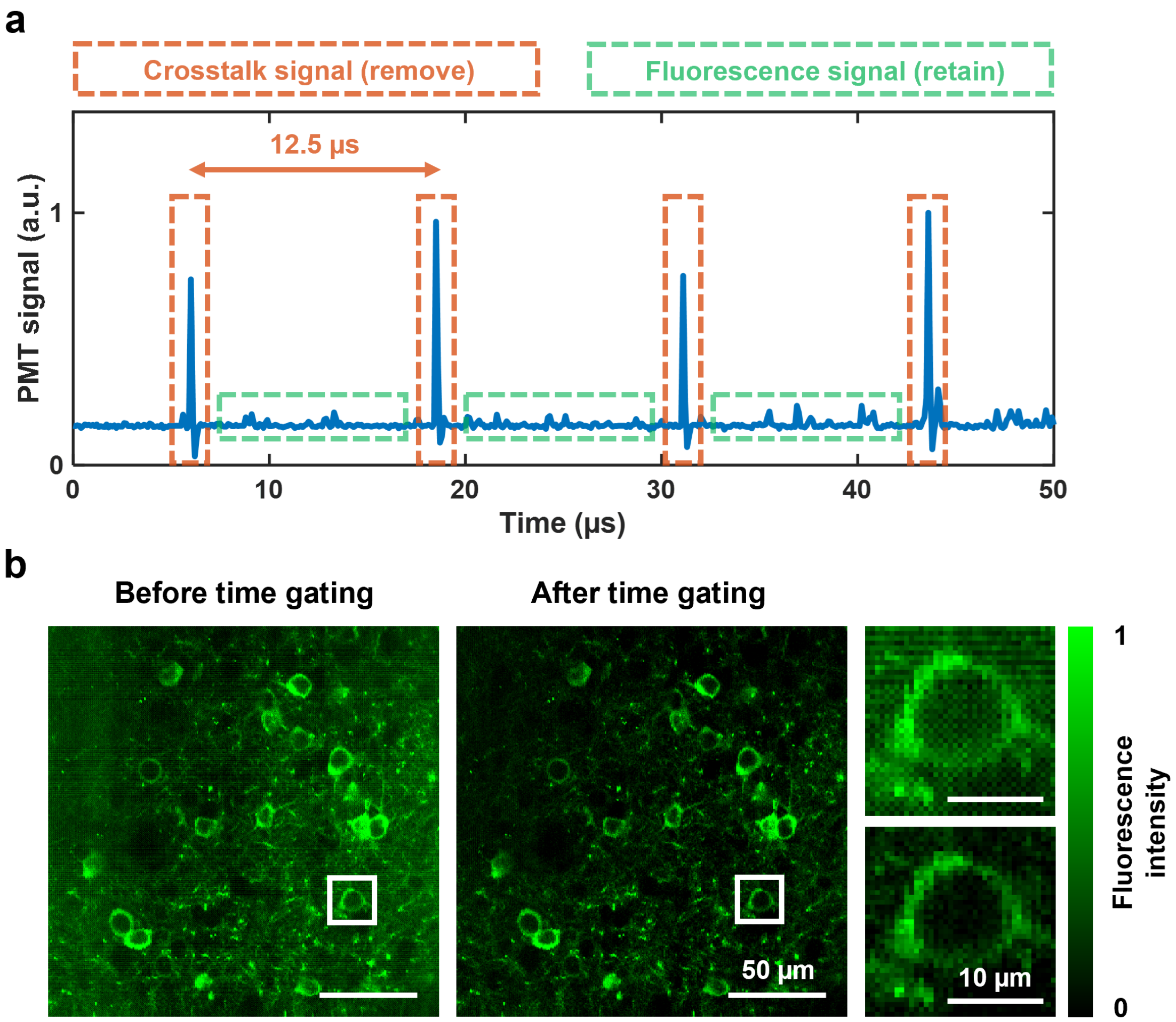

Supplementary Fig. 15 | Time gating to remove crosstalk of PAM laser on TPM imaging. a, Representative crosstalk signals induced by the PAM nanosecond laser during TPM fluorescence detection by the photomultiplier tube (PMT). The crosstalk signals occur every 12.5 μs, corresponding to the 80 kHz repetition rate of PAM laser, which can be removed by precise time gating. **b,** TPM images of neurons *in vivo* before and after time gating. The right column shows a zoomed inset of a single neuron (indicated by white boxes in the two images on the left), showcasing enhanced image quality after removing the crosstalk.

Supplementary Table 1. Scanning parameters used in all imaging experiments

	Raster scanning				Kymographic scanning									Effective frame rate (Hz)
	FOV (μm)	pixel number	step size (μm)	time (s)	loop #1		loop #2		loop #3		loop #4		time (s)	
					pts.	c.	pts.	c.	pts.	c.	pts.	c.		
**Fig.1 c-e**	400	400	1.0	2.00	400	120							0.60	0.43
**Fig.1 f-k**	150	150	1.0	0.28	100	40	350	40					0.23	2.54
**Fig.2**	200	150	1.3	0.28	50	40	40	40	360	40			0.23	2.54
**Fig.3**	150	150	1.0	0.28	300	60							0.23	2.54
**Fig.4**	200	150	1.3	0.28	40	40	50	40	50	40	460	40	0.30	2.32

Supplementary References

1. Li, H. *et al.* Disposable ultrasound-sensing chronic cranial window by soft nanoimprinting lithography. *Nat Commun* **10**, 4277 (2019).

2. Rong, Q. *et al.* High-Frequency 3D Photoacoustic Computed Tomography Using an Optical Microring Resonator. *BME Frontiers* **2022**, 9891510 (2022).

3. Xu, G. *et al.* Acoustic Characterization of Polydimethylsiloxane for Microscale Acoustofluidics. *Phys. Rev. Appl.* **13**, 054069 (2020).

4. Lin, D.-S., Zhuang, X., Wong, S. H., Kupnik, M. & Khuri-Yakub, B. T. Encapsulation of Capacitive Micromachined Ultrasonic Transducers Using Viscoelastic Polymer. *Journal of Microelectromechanical Systems* **19**, 1341–1351 (2010).

5. Xie, Y. *et al.* Acoustic Cell Separation Based on Density and Mechanical Properties. *J Biomech Eng* **142**, 0310051–0310059 (2020).

6. MY-131-MC: Optical Coating Material. *MY Polymers* https://www.mypolymers.com/p/2255/.

7. MY-130: Optical Coating / Adhesive. *MY Polymers* https://www.mypolymers.com/p/2268/.

8. Rae, P. j. & Brown, E. n. Some Observations on Measuring Sound Speeds in Polymers Using Time-of-Flight. *Experimental Techniques* **n/a**,.
