# Supplementary material for: Integrated two-photon and photoacoustic microscopy for single-cell neurometabolic imaging
Source: Nat Commun. 2026 Jul 15;17:8670. doi: 10.1038/s41467-026-75603-7 (PMC13490390; doi:10.1038/s41467-026-75603-7)
Supplement: Supplementary file 2 [file 41467_2026_75603_MOESM2_ESM.pdf]

Description of Additional Supplementary Files

Supplementary Movie 1. This video corresponds to Fig. 1f–k in the main manuscript. It presents simultaneous recordings of neuronal activity, vascular hemoglobin concentration (CHb), oxygen saturation (sO2), and sO2-encoded kymograph in the awake GCaMP mouse brain. The extracted traces show calcium dynamics from seven neurons, along with sO2 and blood flow dynamics from four capillaries. The brief pause in the video highlights the oxygen release process from a single red blood cell (RBC) during its passage through capillary C1. This result demonstrates the capability of the TPM-PAM platform for high-resolution, high-sensitivity monitoring of neurometabolic dynamics at the single-cell level in vivo.

Supplementary Movie 2. This video corresponds to Fig. 2 in the main manuscript. It presents simultaneous recordings of neuronal activity, vascular hemoglobin concentration (CHb), oxygen saturation (sO2), and sO2-encoded kymograph in the awake GCaMP mouse brain during ten repeated trials of whisker stimulation. The extracted traces show calcium dynamics from nine neurons, along with responses of eight oxygen-metabolic parameters derived from arterioles (red), venules (dark blue), capillaries (light blue), and the entire field of view (FOV, gray). The green bars indicate the stimulation periods.

Supplementary Movie 3. This video corresponds to Fig. 3 in the main manuscript. It presents simultaneous recordings of neuronal activity, vascular hemoglobin concentration (CHb), oxygen saturation (sO2), and sO2-encoded kymograph in the awake GCaMP mouse brain during targeted single-capillary occlusion. The extracted traces show calcium dynamics from forty neurons, along with oxygen release rate (rO2) dynamics from seven capillaries. The brief pause in the video highlights the occlusion of capillary P1. Following occlusion, the target capillary disappeared from the vascular map, accompanied by sustained calcium activity in nearby neurons.

Supplementary Movie 4. This video corresponds to Fig. 4 in the main manuscript. It presents simultaneous recordings of neuronal activity, vascular hemoglobin concentration (CHb), oxygen saturation (sO2), and sO2-encoded kymograph in the awake GCaMP mouse brain during targeted single-neuron stimulation. The extracted traces show calcium

dynamics from twenty-four neurons, along with oxygen release rate (rO2) dynamics from five capillaries. The brief pause in the video highlights the stimulation of neuron N1. This targeted stimulation induced not only prolonged activation of the neuron itself but also transient calcium responses in neighboring neurons, likely through synaptic network propagation.
