# Supplementary material for: Integrated two-photon and photoacoustic microscopy for single-cell neurometabolic imaging
Source: Nat Commun. 2026 Jul 15;17:8670. doi: 10.1038/s41467-026-75603-7 (PMC13490390; doi:10.1038/s41467-026-75603-7)
Supplement: Supplementary file 3 [file 41467_2026_75603_MOESM7_ESM.pdf]

nature portfolio

Peer Review File

Integrated two-photon and photoacoustic microscopy for single-cell neurometabolic imaging

Corresponding Author: Professor Song Hu

This file contains all reviewer reports in order by version, followed by all author rebuttals in order by version.

Version 0:

Reviewer comments:

Reviewer #1

(Remarks to the Author)
The authors introduce integrated two-photon and photoacoustic microscopy (TPM-PAM) based on their developed novel micro-ring resonator ultrasound sensor, which enables simultaneous imaging of neuronal dynamics and oxygen metabolism at single-cell resolution in vivo. They also incorporate nonlinear optical manipulation of the neurovascular unit into the imaging system, making it a powerful platform for neurometabolic coupling research. In vivo experiments on awake mice have demonstrated the unique performance of this platform as an effective tool for imaging and modulation of neurometabolic coupling. This work presents a significant methodological advance and is insightful for understanding oxygen-metabolic regulation in brain health and disease. I am very much enthusiastic about the work and support the publication in Nature Communications. Here are some suggestions to strengthen the manuscript.
Major comments:
1. Integrated photoacoustic/fluorescence microscopy has been proven to be an effective tool for NVC imaging. Several approaches have been reported or shown potential for the integration of these two modalities. In addition to the scheme based on miniaturized ultrasonic transducers mentioned in the Introduction section, there are other schemes which could be realized based on transparent ultrasound transducers (TUTs)[1][2], photoacoustic remote sensing (PARS)[3] or optical sensors such as fiber Bragg gratings (FBGs) [4] . Please include a full comparison of these different technical approaches.

Ref:
[1] Mirg, S. et al. Awake mouse brain photoacoustic and optical imaging through a transparent ultrasound cranial window. Opt. Lett. 47, 1121 (2022).
[2] Qiu, C. Transparent ultrasonic transducers based on relaxor ferroelectric crystals for advanced photoacoustic imaging. Nature Communications 15, 10580 (2024).
[3] Zhou, J., Wang, W., Jing, L. & Chen, S.-L. Dual-modal imaging with non-contact photoacoustic microscopy and fluorescence microscopy. Opt. Lett. 46, 997 (2021).
[4] Zhong, X. et al. Free-moving-state microscopic imaging of cerebral oxygenation and hemodynamics with a photoacoustic fiberscope. Light Sci Appl 13, 5 (2024).

2. Descriptions of the scanning method adopted in the imaging experiments are not quite clear. Please clarify it in the manuscript or the supplementary videos. Were all the imaging experiments performed using a combination of raster scanning and kymographic scanning? Specifically, how was the sequence designed? Was kymographic scanning performed after each frame of raster scanning, or after several consecutive frames of imaging? Additionally, what is the stability of the combination of raster scanning and kymographic scanning? Will kymographic scanning cause disturbances to the stability of the galvanometer during raster scanning at a 3 Hz frame rate? Moreover, when kymographic scanning needs to scan different numbers of blood vessels (such as 4 capillaries in Fig.1h and 7 capillaries in Fig.3a) in different experiments, will it affect the imaging frame rate?

3.The independent and active modulation of individual neurons or microvessels represents an impactful experimental paradigm for investigating NVC. Moreover, this exploration holds substantial significance for investigating the mechanisms, diagnosis and treatment of cerebral disorders such as ischemic stroke. The manuscript presents the experimental procedures and results technically, while lacking elaboration on the research background. It is recommended to add the scientific significance and broader implications of the modulation experiments to make the manuscript more attractive to a wider research community.

4.The spatial analysis of NVC illustrated in Fig.3e and Fig.4e is impressive and insightful. However, since the number of neurons and capillaries recorded in the FOV are relatively small, it may raise concerns about the accuracy of the analysis. Is the coupling characteristic presented in the figures derived solely from this single observation, or is it generalizable to some extent? It is recommended to provide some discussions and future improvements in the Discussion section.

5.What are the advantages and disadvantages of directly stimulating neurons by using focused femtosecond laser beams compared to adopting optogenetic protocols? The latter is the most common approach for optical neuromodulation. Will the high-energy focused laser beam utilized in the single-neuron stimulation experiment have adverse effects on the target neuron?

6. The authors mention optimizing the cladding materials of the MRR to enhance the ultrasound detection sensitivity of PAM. Specifically, they changed the top cladding from PDMS (refractive index ≈1.40) to a low-refractive-index optical acrylic resin (~1.31). Could the authors provide specific parameters of this optical acrylic resin and directly compare it with PDMS? Additionally, should the acoustic matching between the material and water also be considered, rather than focusing solely on improving the Q-factor? A more detailed discussion on this point would be helpful.

7. The authors refer to anti-Stokes signals induced by the PAM nanosecond laser as a source of crosstalk in TPM imaging. Are these anti-Stokes signals generated by stimulated Raman scattering in the fiber? Can such signals not be removed by narrow-band optical filters used in the paper? In my understanding, could the crosstalk actually arise from back-reflected Stokes light from the sample? Could the authors provide additional experimental data to support their conclusion and further clarify this mechanism?

Minor comments:
1.The colors of the upper part of Fig.3c and Fig.4c are too pale to see clearly. Please revise them.

2.It is recommended to provide detailed imaging parameters such as the pixel numbers, pulse repetition rate and pulse energy for the in vivo imaging experiment in the Methods section or the supplementary file.

3.What are the depths of field of TPM and PAM in the imaging system? What are the depths of the neurons and blood vessels, respectively?

4.In the experiment of single-capillary occlusion, supplementary video 3 shows that an abnormal fluorescence signal appeared at 83.2 s (marked by the red rectangle in the following image). How to explain this interesting phenomenon? It is quite similar to the imaging result of single-neuron stimulation shown in supplementary video 4. Does the 800-nm fs laser beam used to occlude the blood vessel have an impact on the nearby neurons? Or is it the physiological response of the neurons by the moment when the blood vessel (marked by P1) is occluded?

5. Please ensure the numerical labeling (e.g., DM1, DM2, …) for "DM" is consistent between Fig. 1 and Extended Fig. 3 to improve readability.

Reviewer #2

(Remarks to the Author)
This manuscript presents a comprehensive study of neurometabolic coupling (NMC) at the level of single microvessels and individual neurons, enabled by an innovative dual-modal imaging platform that unified two-photon fluorescence microscopy (for GCaMP-based neural activity imaging) with photoacoustic imaging for quantitative hemodynamic assessment. A key technical innovation is the development of a thin, optically transparent, and acoustically sensitive micro-ring resonator (MRR) ultrasound detector, which permits the passage of a tightly focused excitation beam for two-photon imaging (as well as beams for single-capillary occlusion and single-neuron stimulation).
In vivo quantitative imaging demonstrates the feasibility of this unified platform for assessing multiple NMC-related parameters, including arteriole diameter, blood flow velocity, volumetric flow rate, oxygen saturation, oxygen flux, oxygen release rate, and oxygen extraction fraction. The study design is scientifically sound, the results are comprehensive, and the conclusions are well supported by appropriate analyses. Overall, this platform is highly valuable and has strong potential to enable fundamental investigations of neurological diseases.
While this is an excellent manuscript, a few points would benefit from clarification prior to publication. Most of the concerns are minor:
1. Sample size and experimental design.
Please specify the number of mice used for each type of experiment. Were experiments repeated across different days? If so, how many sessions were performed per mouse on each day? These details are important for determining the appropriate statistical model.
2. Imaging depth and cortical layer.
Which cortical layer(s) of the somatosensory cortex were imaged? Please provide the approximate imaging depth below the dura.
3. Field-of-view (FOV) selection.
Please briefly describe the criteria used to select imaging FOVs. If FOVs were selected randomly, please state it explicitly in the manuscript.
4. Data presentation.

Where appropriate, consider presenting quantitative results in the form of (mean ± SD).
5. Line 95–96: “four-fold improvement in coupling.”
Please clarify what “coupling” refers to (e.g., for the single-model light in and out of the MRR for acoustic signal detection?). Additionally, what specific design features contributed to this improvement—MRR material properties, thickness, geometric dimensions, or fabrication precision (e.g., ring dimensional control)?
6. Line 137: axial resolution.
The reported axial resolution of 2.4 µm appears unusually high for the stated 0.7 µm lateral resolution. Please clarify the method used to characterize axial resolution, as the value may depend on the measurement approach or robustness.
7. Line 147: Radon transform.
Please briefly explain what the Radon transform was used for in kymography-based flow speed measurements, which can be valuable to for readers who may be less familiar with this approach.
8. Lines 184–189: quantitative changes.
The manuscript reports a 35% increase in CBF and a 70% rise in fO_2. Were these values directly measured, or analytically derived from other measured parameters? Please clarify.
9. Lines 197–204: statistical analysis.
Given the expected inter-animal variability and potentially limited sample size, the assumption of Gaussian distribution may not hold and thus it is unclear if t-test would be suitable for statistical analysis.

Version 1:

Reviewer comments:

Reviewer #1

(Remarks to the Author)
I appreciate the authors’ thoughtful and thorough responses to all of my comments. The concerns raised during the review process have been satisfactorily addressed, and the manuscript has been substantially improved. I believe the revised manuscript is suitable for publication in its current form and support its acceptance.

Reviewer #2

(Remarks to the Author)
This reviewer greatly appreciates the authors’ efforts in comprehensively revising the manuscript. The revised manuscript has adequately addressed the previous comments. This reviewer has no further questions or comments.

Open Access This Peer Review File is licensed under a Creative Commons Attribution 4.0 International License, which permits use, sharing, adaptation, distribution and reproduction in any medium or format, as long as you give appropriate credit to the original author(s) and the source, provide a link to the Creative Commons license, and indicate if changes were made.
In cases where reviewers are anonymous, credit should be given to 'Anonymous Referee' and the source.
The images or other third party material in this Peer Review File are included in the article’s Creative Commons license,

unless indicated otherwise in a credit line to the material. If material is not included in the article's Creative Commons license and your intended use is not permitted by statutory regulation or exceeds the permitted use, you will need to obtain permission directly from the copyright holder.
To view a copy of this license, visit https://creativecommons.org/licenses/by/4.0/

Reviewer #1 (Remarks to the Author):

The authors introduce integrated two-photon and photoacoustic microscopy (TPM-PAM) based on their developed novel micro-ring resonator ultrasound sensor, which enables simultaneous imaging of neuronal dynamics and oxygen metabolism at single-cell resolution in vivo. They also incorporate nonlinear optical manipulation of the neurovascular unit into the imaging system, making it a powerful platform for neurometabolic coupling research. In vivo experiments on awake mice have demonstrated the unique performance of this platform as an effective tool for imaging and modulation of neurometabolic coupling. This work presents a significant methodological advance and is insightful for understanding oxygen-metabolic regulation in brain health and disease. I am very much enthusiastic about the work and support the publication in Nature Communications. Here are some suggestions to strengthen the manuscript.

Response:

Thank you for the positive feedback and valuable evaluation of our work. Our detailed responses to the specific comments are provided below.

Major comments:

1. Integrated photoacoustic/fluorescence microscopy has been proven to be an effective tool for NVC imaging. Several approaches have been reported or shown potential for the integration of these two modalities. In addition to the scheme based on miniaturized ultrasonic transducers mentioned in the Introduction section, there are other schemes which could be realized based on transparent ultrasound transducers (TUTs)[1][2], photoacoustic remote sensing (PARS)[3] or optical sensors such as fiber Bragg gratings (FBGs) [4] . Please include a full comparison of these different technical approaches.

Ref:

[1] Mirg, S. et al. Awake mouse brain photoacoustic and optical imaging through a transparent ultrasound cranial window. Opt. Lett. 47, 1121 (2022).

[2] Qiu, C. Transparent ultrasonic transducers based on relaxor ferroelectric crystals for advanced photoacoustic imaging. Nature Communications 15, 10580 (2024).

[3] Zhou, J., Wang, W., Jing, L. & Chen, S.-L. Dual-modal imaging with non-contact photoacoustic microscopy and fluorescence microscopy. Opt. Lett. 46, 997 (2021).

[4] Zhong, X. et al. Free-moving-state microscopic imaging of cerebral oxygenation and hemodynamics with a photoacoustic fiberscope. Light Sci Appl 13, 5 (2024).

Response:

Thank you for this constructive suggestion. We fully agree that a comprehensive comparison

of different technical approaches is essential to justify the adoption of the micro-ring resonator in this work.

When evaluating different ultrasound sensors for the integration of TPM and PAM for neurovascular imaging, we should consider the following criteria: **(1) High detection sensitivity and wide bandwidth** to achieve sufficient signal-to-noise ratio for low-amplitude photoacoustic signals of capillaries, ensuring clear identification of capillary structures and quantification of oxygen dynamics at the single-RBC level. Wide bandwidth is essential because the center frequency and bandwidth of excited photoacoustic signals both scale inversely with the absorber size: signals from single RBCs (~5 μm diameter) in capillaries contain higher and broader frequency components (often >50 MHz) than those from RBC clusters in larger vessels. Therefore, high sensitivity at low frequencies alone is insufficient, and wide bandwidth is needed to capture the high-frequency components of capillary signals. **(2) High optical transparency** across both visible and near-infrared wavelengths, particularly at the photoacoustic excitation wavelengths (545 and 558 nm) and the two-photon excitation wavelength (930 nm), to minimize the optical attenuation and aberration induced by the ultrasound sensor. **(3) Low thickness** to allow its placement beneath the high-NA objective, which typically has a limited working distance (2 mm in this work). **(4) Compatible with laser scanning** to enable high-speed imaging of microvascular dynamics over a considerable field of view (>200 × 200 μm^2), rather than relying on slow mechanical scanning.

Piezoelectric-based transparent ultrasound transducers (Table R1) have facilitated the integration of PAM with other optical imaging modalities. A range of transparent piezoelectric materials, including PVDF (polyvinylidene fluoride)[1], LN (lithium niobate)[2,3], PMN-PT (lead magnesium niobate–lead titanate)[3], and PIN-PMN-PT (lead indium niobate-lead magnesium niobate-lead titanate)[4], have been explored, achieving a noise equivalent pressure (NEP) down to several pascals. Despite these advances, **the bandwidths of these transducers are typically limited to <30 MHz**. This limitation mainly arises from the low acoustic impedance of epoxy-based matching layers (~3 MRayl), which is insufficient to bridge the large acoustic impedance mismatch between piezoelectric materials (LN, PMN-PT, PIN-PMN-PT, ~30 MRayl) and water (~1.5 MRayl), leading to considerable acoustic reflection and the attenuation of high-frequency acoustic components[5]. **The restricted bandwidth limits the sensitivity to capillary photoacoustic signals and hinders the performance for neurovascular imaging at the single-cell level.** Future exploration of transparent matching materials with enhanced acoustic impedance will be critical to overcoming this limitation.

Table R1. Summary of Piezoelectric-based Transparent Ultrasound Transducers (TUT)				
**Piezoelectric material**	**Focused PVDF[1]**	**LN[2,3]**	**PMN-PT[3]**	**PIN-PMN-PT[4]**
**Sensitivity (NEP)**	lower sensitivity than LN-based TUT[5]	36 Pa	18 Pa	9 Pa
**Center frequency**	24 MHz	7.2/11.8 MHz	7.8/13.2 MHz	28.5 MHz
**Bandwidth (-3 dB)**	26 MHz	10 MHz	10 MHz	22 MHz
**Optical transmittance (532 nm | 930 nm)**	60% | 57%	91% | 87%	73% | 73%	70% | 60%
**Thickness**	~0.5 mm (with curved substrate)	2.39 mm (with backing layer)	2.34 mm (with backing layer)	0.72 mm (with backing layer)
**Field of view with laser scanning**	0.13 × 0.13 mm^2 (acoustic focal spot)	(2–6) × (2–6) mm^2 (depending on material size)		

Optical-based transparent ultrasound sensors (Table R2) mainly include MRR (micro-ring resonator)[6,7], free-space or fiber-based FP (Fabry-Perot)[8] and fiber laser sensors[9,10], all of which feature high optical transparency and compact form factors desired for multimodal integration. Among them, FP sensors have the largest acceptance angle and can be readily configured as detector arrays, either in free space[11] or at fiber-bundle tips[12], for photoacoustic tomography. However, locking the probe laser wavelength to the resonance frequency of FP sensors is highly susceptible to thermal drifts. By using heterodyne phase detection at a fixed wavelength, fiber laser sensors are insensitive to temperature fluctuations and demonstrate superior stability[13]. Despite these advantages, **both FP sensors and fiber laser sensors are also constrained by the limited detection bandwidth (<30 MHz)**, making them suboptimal for single-RBC imaging. In contrast, **MRR offers the highest sensitivity and largest bandwidth** among the optical-based ultrasound sensors, making it ideally suited for single-cell neurovascular imaging presented in this work.

Table R2. Summary of Optical-based Transparent Ultrasound Sensors			
	**MRR**[6,7]	**Fiber-based FP**[8]	**Fiber laser sensor**[9,10]
**Sensitivity (NEP)**	0.49 Pa	9.3 Pa	6.7 Pa
**Center frequency**	56 MHz	14 MHz	8/22 MHz
**Bandwidth (-3 dB)**	100 MHz	20 MHz	20 MHz
**Thickness**	0.37 mm (with substrate)	0.125 mm (fiber diameter)	0.125 mm (fiber diameter)
**Acceptance angle (field of view at 1 mm distance)**	±15° @ 50 MHz (0.54×0.54 mm^2)	±90° @ 22 MHz	0–60° (1.7×3 mm^2)

Photoacoustic remote sensing microscopy (PARS) eliminates the need for ultrasound sensors, enabling non-contact photoacoustic imaging that can be readily integrated with other optical imaging modalities[14]. By using a low-coherence beam to probe the reflectivity modulation induced by the initial pressure rise, PARS has demonstrated *in vivo* microvascular imaging with a sensitivity comparable to conventional transducer-based PAM and a detection bandwidth of up to 54 MHz[15,16]. Despite these advantages, its application in functional imaging, such as measuring the oxygen saturation of hemoglobin (sO_2), remains to be fully established[17,18]. Notably, theoretical predictions of the initial pressure-induced reflectivity modulations have been found to be orders of magnitude smaller than those typically observed experimentally[19]. This discrepancy suggests that **the detected PARS signals may also arise from other nonlinear effects, such as laser pulse-induced absorber motion or microbubble formation,** as previously reported[19]. Because the spectroscopic measurement of sO_2 relies on a linear relationship between signal amplitude and optical absorption, these nonlinear mechanisms could compromise measurement fidelity. Therefore, further understanding of the mechanisms underlying PARS signal generation is required to address these concerns and ensure reliable functional measurements in neurovascular research.

We have included a concise discussion in the Introduction section of the revised manuscript (Lines 69–77).

2. Descriptions of the scanning method adopted in the imaging experiments are not quite clear. Please clarify it in the manuscript or the supplementary videos. Were all the imaging experiments performed using a combination of raster scanning and kymographic scanning? Specifically, how was the sequence designed? Was kymographic scanning performed after

each frame of raster scanning, or after several consecutive frames of imaging? Additionally, what is the stability of the combination of raster scanning and kymographic scanning? Will kymographic scanning cause disturbances to the stability of the galvanometer during raster scanning at a 3 Hz frame rate? Moreover, when kymographic scanning needs to scan different numbers of blood vessels (such as 4 capillaries in Fig.1h and 7 capillaries in Fig.3a) in different experiments, will it affect the imaging frame rate?

Response:

Thank you for this constructive suggestion.

In this work, all experiments were performed using a combination of raster scanning and kymographic scanning, with one kymographic scan conducted after every two consecutive raster scans.

Here is the procedure for designing the scanning path:

Step 1: Perform raster scanning to obtain a PAM image of the vasculature.

Step 2: Visualize the acquired image in MATLAB and manually define an initial kymographic path. This path forms a closed loop covering the central axes of all vessels of interest, typically consisting of 300–600 uniformly spaced sampling points.

Step 3: Load the path into LabVIEW to drive the galvanometer for kymographic scanning, where the light focus is repeatedly scanned along the path to evaluate whether the blood flow in each vessel is resolvable (i.e., whether a clear RBC motion slope is visible in the kymograph). With an 80-kHz laser repetition rate, a path with 300 sampling points corresponds to a line rate of 267 Hz. Assuming a maximum distinguishable RBC displacement of 10 μm between adjacent lines, this line rate translates to a maximum resolvable flow speed of ~2.7 mm/s. While sufficient for most capillaries, this may fall short for measuring faster flow in some arteries.

Step 4: Refine the path design by assigning an independent closed loop to each vessel whose flow is too fast to be resolved in Step 3, while grouping the remaining vessels into a separate loop (e.g., allocating 100 sampling points for a fast-flow vessel and 200 for the rest). Each loop is repeatedly scanned for ~40 cycles before the system proceeds to the next loop. This multi-loop strategy effectively increases the line rate for fast-flow vessels (from 267 Hz to 800 Hz in this work), thereby extending the maximum resolvable flow speed from ~2.7 mm/s to ~8 mm/s. The final path typically consists of 1–4 loops with step sizes of 2–5 μm. Notably, simply applying a high line rate (e.g., 800 Hz) to all vessels via a single large loop is not preferred, as the galvanometer may not reliably track the large loop at such a high speed. Instead, assigning a high line rate only to fast-flow vessels within relatively small spatial regions ensures galvanometer stability.

Step 5: Perform TPM-PAM imaging experiment using the optimized multi-loop path, with every two raster scans followed by one kymographic scan.

The scanning parameters and paths for all imaging experiments in this work are summarized in Table R3 and Fig. R1. Using the parameters from Fig. 1f-k as an example, the imaging field of view (FOV) is 150 × 150 μm^2 with 150 × 150 pixels, corresponding to a step size of 1 μm. Given a PAM laser repetition rate of 80 kHz, each raster scan takes 0.28 s. After two raster scans, a kymographic scan is performed using two closed loops with 100 and 350 sampling points (pts.), which correspond to line rates of 800 Hz and 230 Hz, respectively. Each loop is repeated for 40 cycles (c.). Each kymographic scan takes 0.23 s. Together, this combined scanning strategy yields an effective raster frame rate of 2.54 Hz and an effective kymography frame rate of 1.27 Hz.

Table R3. Summary of Scanning Parameters

	Raster scanning				Kymographic scanning									Effective frame rate (Hz)
	FOV (μm)	pixel number	step size (μm)	time (s)	loop #1		loop #2		loop #3		loop #4		time (s)	
					pts.	c.	pts.	c.	pts.	c.	pts.	c.		
**Fig.1 c-e**	400	400	1.0	2.00	400	120							0.60	0.43
**Fig.1 f-k**	150	150	1.0	0.28	100	40	350	40					0.23	2.54
**Fig.2**	200	150	1.3	0.28	50	40	40	40	360	40			0.23	2.54
**Fig.3**	150	150	1.0	0.28	300	60							0.23	2.54
**Fig.4**	200	150	1.3	0.28	40	40	50	40	50	40	460	40	0.30	2.32

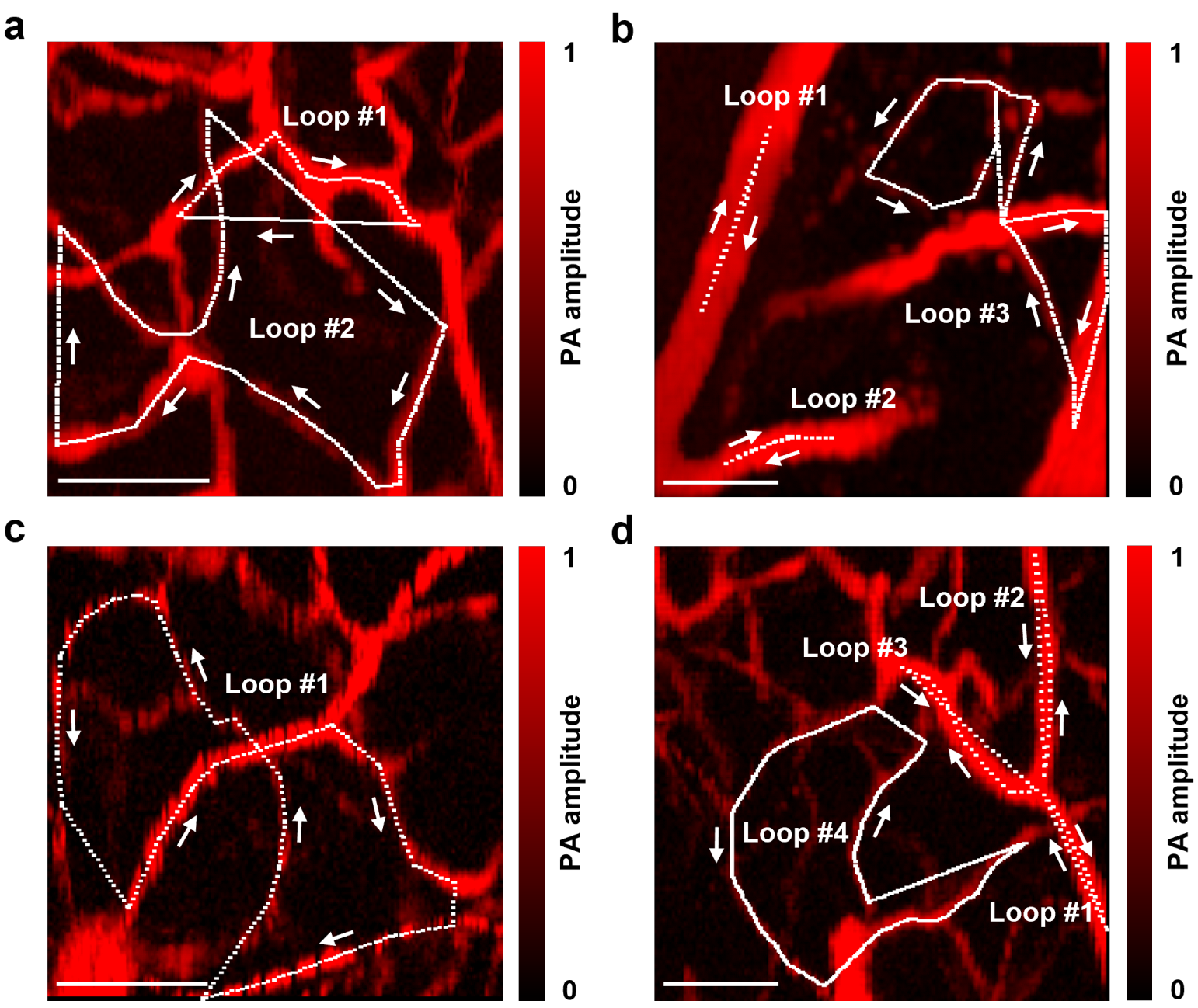

Fig. R1 | Kymographic scanning paths used in the experiments corresponding to Fig. 1f-k (a), Fig. 2 (b), Fig. 3 (c), and Fig. 4 (d). White arrows indicate scanning directions. Scale bar: 50 μm.

To demonstrate the stability of the combined raster and kymographic scanning, we performed a new experiment by imaging a resolution target with standard bar patterns (Fig. R2). First, a reference image was acquired using raster scanning alone (Fig. R2a). Next, the same FOV was imaged using the combined approach, where kymograph scanning across four bar patterns (paths denoted by the green lines in Fig. R2b) was interleaved after every two consecutive raster-scan frames (Fig. R2b). The scanning parameters were identical to those used in the *in vivo* experiments (i.e., FOV: 200 × 200 μm^2; pixel number: 150 × 150; total kymographic sampling points: 450; effective frame rate: 2.54 Hz; imaging duration: 300 s). Finally, a structural similarity (SSIM) analysis was performed to compare the frames acquired using the combined scanning scheme against the raster-only reference. The results show consistently high SSIM values (~0.99) throughout the entire 300-s acquisition period (Fig. R2c), demonstrating that the interleaved kymographic scanning introduces minimal disruption to the stability of raster scanning.

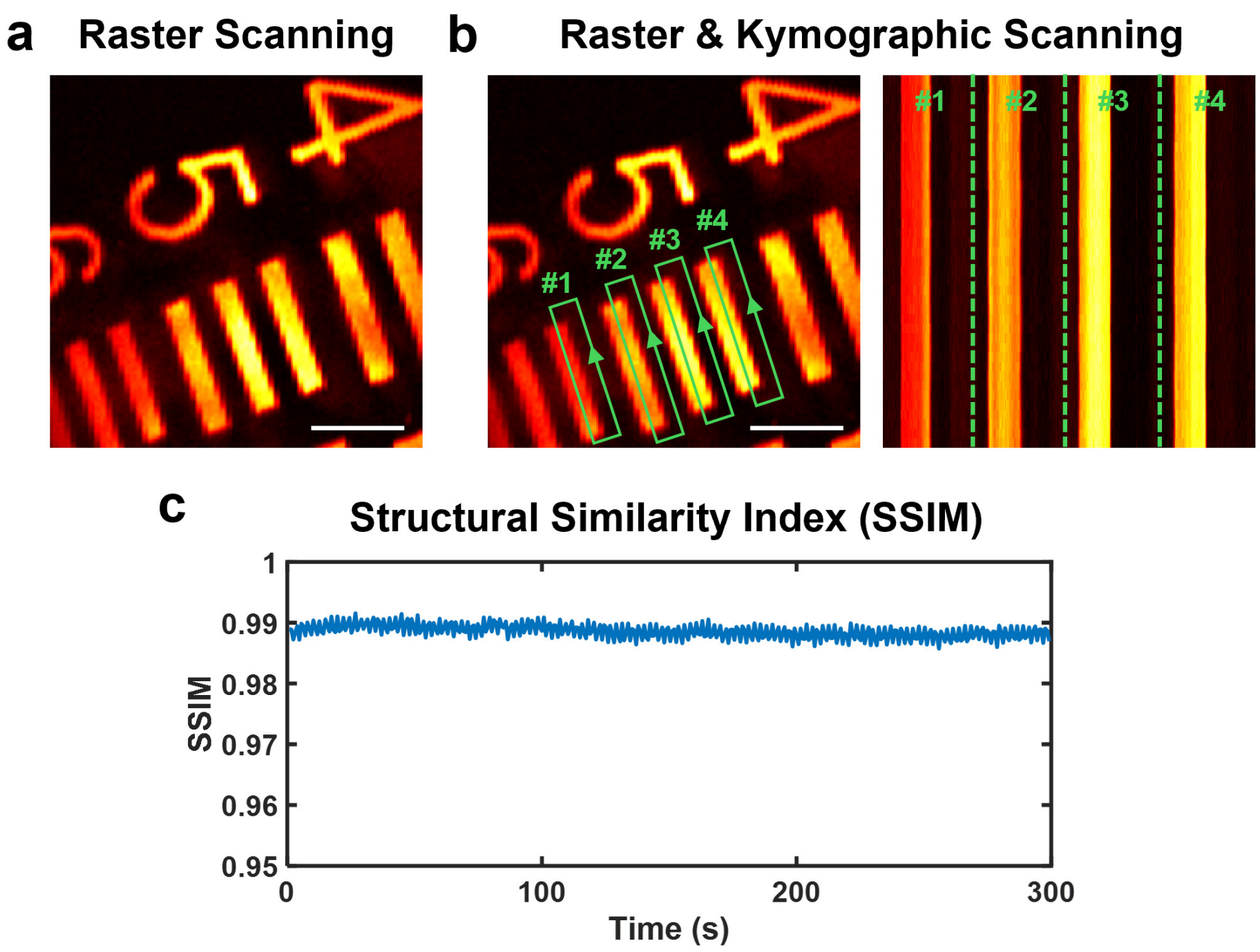

Fig. R2 | Stability evaluation of the combined raster and kymographic scanning. a, Reference image of a resolution target acquired using raster scanning alone. **b,** Imaging results obtained with the combined raster and kymographic scanning scheme (left, raster frame; right, kymographs across four bar patterns). Green lines in the left panel indicate the kymographic scanning paths. **c,** Structural similarity (SSIM) analysis comparing frames acquired with the combined scanning scheme and the raster-only reference over the 300-s acquisition period. Scale bar: 50 μm.

We have updated the Methods section of the main manuscript to provide a clearer description of the scanning scheme (Lines 464–501), together with the newly added Supplementary Fig. 9 and Supplementary Table 1 detailing the scanning paths and parameters.

3.The independent and active modulation of individual neurons or microvessels represents an impactful experimental paradigm for investigating NVC. Moreover, this exploration holds substantial significance for investigating the mechanisms, diagnosis and treatment of cerebral disorders such as ischemic stroke. The manuscript presents the experimental procedures and results technically, while lacking elaboration on the research background. It is recommended to add the scientific significance and broader implications of the modulation experiments to make the manuscript more attractive to a wider research community.

Response:

Thank you for this constructive suggestion. We fully agree that further clarifying the scientific significance of the modulation experiments will make this work more compelling to a wider research community.

The broader implications of the modulation experiments presented in this work (i.e., single-neuron stimulation and single-capillary occlusion) can be summarized in three key aspects:

(1) Establish bidirectional causality of NVC to improve interpretation of clinical neuroimaging data.

Clinical neuroimaging modalities, such as BOLD-fMRI, infer brain activity indirectly by measuring oxygen-hemodynamic changes. Because the BOLD signal represents a composite readout of blood oxygenation and flow, its interpretation remains ambiguous without a mechanistic ground truth[20,21]. In this context, our modulation experiments of single-neuron stimulation and single-capillary occlusion lay the technical foundation for exploring the bidirectional causality of NVC. By enabling the independent perturbation of single-neuron activity and single-vessel oxygen-hemodynamics while simultaneously monitoring the corresponding neurovascular responses, this causal framework allows for explicit dissection of neuron-to-vascular and vascular-to-neuron interactions. Therefore, these experiments hold the potential to provide microscopic insights that are essential for refining NVC models and improving the interpretability of clinical neuroimaging data.

(2) Resolve the micro-scale heterogeneity of NVC.

NVC is highly heterogeneous, varying across vessel types (arteriole, capillary, and venule), spatial distances from active neurons, and local capillary network topologies[22,23]. This intrinsic micro-scale heterogeneity is difficult to capture using conventional

approaches that average signals over a group of neurons and vessels. In contrast, our modulation experiments enable direct mapping of how individual neurons recruit nearby vascular elements and how local capillary perturbations propagate through the network, thereby disentangling vessel-type-specific and spatially dependent NVC mechanisms.

(3) **Model microvascular occlusion and evaluate therapeutic interventions.** Although most ischemic strokes arise from the occlusion of arteries or arterioles, microvascular blockages at the capillary level can induce microinfarcts that underlie neurodegenerative disease[24]. In this work, targeted single-capillary occlusion precisely models microvascular blockage and provides direct observation of how focal ischemia propagates through the capillary network and impairs neuronal function. Moreover, this modulation paradigm offers a platform to evaluate therapeutic interventions—such as reperfusion strategies or neurovascular protective agents[25]—by directly assessing their ability to restore microcirculation, oxygen delivery, and neurovascular coupling at the specific site of injury.

We have updated the Discussion section to further clarify the scientific significance and broader implications of the modulation experiments (Lines 322–327).

4.The spatial analysis of NVC illustrated in Fig.3e and Fig.4e is impressive and insightful. However, since the number of neurons and capillaries recorded in the FOV are relatively small, it may raise concerns about the accuracy of the analysis. Is the coupling characteristic presented in the figures derived solely from this single observation, or is it generalizable to some extent? It is recommended to provide some discussions and future improvements in the Discussion section.

Response:

Thank you for this critical and insightful comment. We fully agree that a careful discussion regarding the accuracy and generalizability of the observations presented in Fig. 3e and Fig. 4e is necessary.

Because these analyses are based on single observations, we acknowledge that they may be subject to statistical uncertainty due to the limited sample size. However, **we wish to clarify that the primary scope of this work is to demonstrate the technical capability of the TPM-PAM system for neurovascular imaging and modulation at the single-cell level, rather than conducting a hypothesis-driven biological study that requires large-scale statistical validation.**

We evaluate the accuracy and scope of these observations from the following perspectives:

(1) FOV vs. oxygen diffusion length

According to the classical Krogh model[26], each capillary supplies oxygen to a surrounding cylindrical tissue volume with a radius of 30–70 μm, corresponding to the oxygen diffusion length. Experimental measurements also show that tissue pO_2 decreases gradually within tens of micrometers from vessels[27], indicating that oxygen delivery is spatially confined to this micro-scale range. Therefore, the FOV used in this work (~200 × 200 μm^2) is sufficient to capture the essential spatial features of local NVC without compromising the accuracy of the spatial analysis.

(2) Future improvements to address limited neuronal imaging volume

One factor that may limit the accuracy of the spatial analyses is the restricted imaging volume of neuronal activity in TPM. In our current implementation, TPM only captures neuronal activity at a single depth and thus might miss information from neurons located within a capillary's oxygen diffusion volume but outside the TPM focal plane. This mismatch in imaging volume between TPM and PAM might lead to partial sampling of neurovascular interactions. To address this issue, advanced strategies for high-speed volumetric TPM, such as spatiotemporal multiplexing, are needed for simultaneous recording of neuronal activity across multiple depths. **We have discussed this as a technical limitation and outlined future developments in the Discussion section (Lines 329–337).**

(3) Generalizability vs. intrinsic micro-scale heterogeneity

To examine the generalizability of the observations presented in Fig. 3e and 4e, we conducted additional experiments of both single-neuron stimulation and single-capillary occlusion in awake GCaMP mice. Our results reveal considerable heterogeneity across animals:

- ***New single-neuron stimulation experiment (Fig. R3):*** Unlike the results shown in Fig. 4 of the main manuscript, the activation of the targeted neuron (N1) did not immediately trigger calcium responses in neighboring neurons. This discrepancy suggests the intrinsic heterogeneity of synaptic connectivity in the mouse brain.

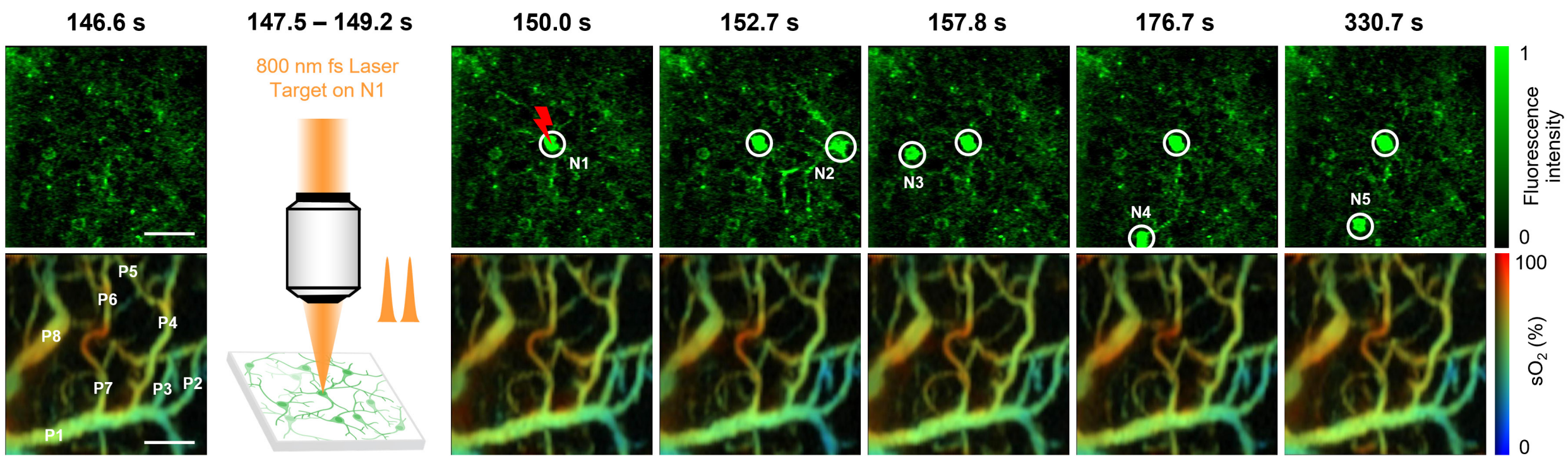

Fig. R3 | TPM-PAM snapshots of single-neuron stimulation in the awake GCaMP

mouse brain. Top row, TPM images; bottom row, PAM sO_2 maps. Red lightning marks the stimulation site. Scale bar: 50 μm.

- ***New single-capillary occlusion experiment (Fig. R4):*** In contrast to the immediate disappearance of the targeted capillary shown in Fig. 3 of the main manuscript, the targeted capillary (P1) exhibited PAM signals from clotted RBCs following manipulation and gradually diminished thereafter. In addition, adjacent capillaries P2 and P4 displayed intermittent non-perfusion, differing from the observations in Fig. 3 and reflecting the topological heterogeneity of local capillary networks.

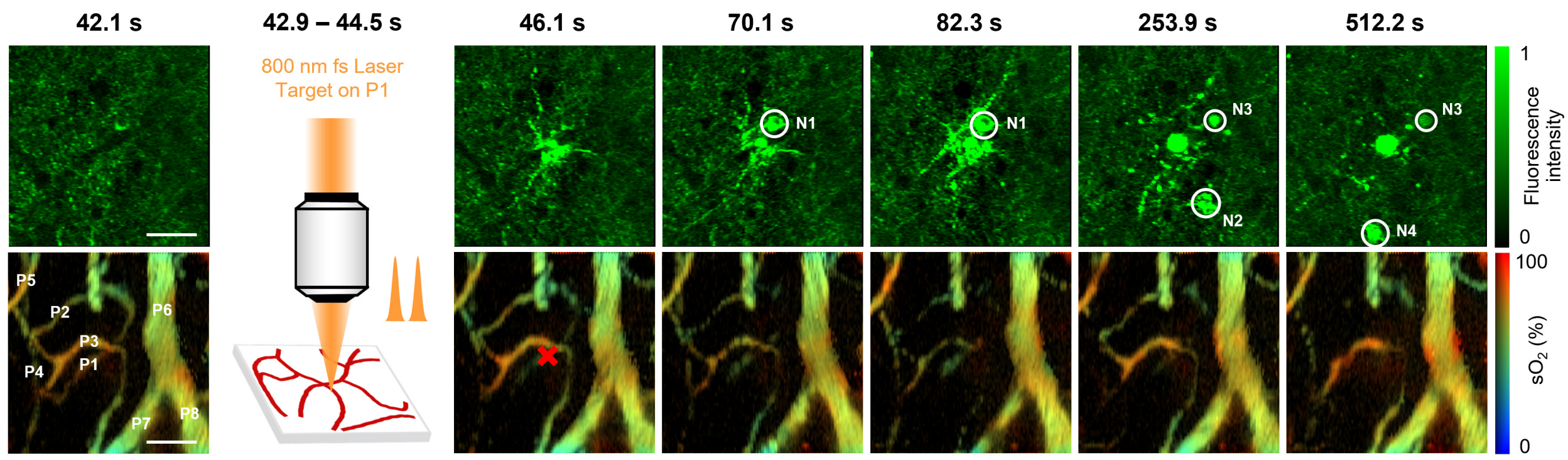

Fig. R4 | TPM-PAM snapshots of single-capillary occlusion in the awake GCaMP mouse brain. Top row, TPM images; bottom row, PAM sO_2 maps. Red cross marks the occlusion site. Scale bar: 50 μm.

- ***Discussion*:** The clear repeatability across 10 trials in our whisker stimulation study on the same animal demonstrates the robustness of our TPM-PAM measurements (Fig. 2 of the manuscript). Thus, the inter-animal differences observed in the single-neuron stimulation and single-capillary occlusion experiments are likely driven by biological heterogeneity. **Rather than viewing the distinct neurovascular responses observed in individual animals as a limitation, we think they reflect the intrinsic heterogeneity of NVC at the micro-scale.** Conventional mesoscopic studies average the responses across groups of neurons and vessels, effectively masking these critical single-event dynamics. In contrast, the single-cell imaging and manipulation enabled by TPM-PAM is well suited to reveal this hidden complexity. Generalizing these local, micro-scale neurovascular responses will require future studies explicitly designed to map, rather than average out, the intrinsic heterogeneity of synaptic connectivity and capillary network topology.

We have updated the Discussion section to outline future technical developments that will enhance the accuracy of our spatial analysis (Lines 329–337) and to address the generalizability of our observations (Lines 319–322).

5.What are the advantages and disadvantages of directly stimulating neurons by using focused femtosecond laser beams compared to adopting optogenetic protocols? The latter is the most common approach for optical neuromodulation. Will the high-energy focused laser beam utilized in the single-neuron stimulation experiment have adverse effects on the target neuron?

Response:

Thank you for this critical comment.

We have summarized the relative advantages and disadvantages of focused femtosecond laser stimulation *vs.* optogenetics below. Also, we have explicitly addressed the potential adverse effects of the high-energy femtosecond laser on the target neuron.

Pros of optogenetics: **(1) Reversibility**. Both optogenetic activation and inhibition are rapidly reversible, allowing for precise temporal control of neuronal activity without permanent cellular damage. **(2) Cell-type specificity**. Optogenetics allows selective manipulation with high cellular specificity via targeted opsin expression using genetic promoters. **(3) Scalable manipulation volume**. Optogenetics can be flexibly scaled from large populations to single neurons. One-photon excitation supports wide-field activation of large volumes, while two-photon excitation supports stimulation of single neurons or groups of neurons when combined with holographic approach[28].

Cons of optogenetics: Crosstalk between imaging and manipulation. Commonly used optogenetic opsins all exhibit substantial one-photon absorption at the PAM excitation wavelengths of 545 nm and 558 nm (e.g., ChR2 absorption spectrum: 400–570 nm; C1V1: 400–600 nm; ReaChR: 400–700 nm). When incorporated into the TPM-PAM system, this spectral overlap induces unintended optogenetic activation by the PAM laser during image acquisition. Furthermore, the high peak power of the nanosecond PAM laser (pulse energy: 100 nJ; pulse width: 5 ns; peak power: 20 W; average power: 8 mW) can lead to the phototoxicity or photodamage of opsin. To assess the crosstalk *in vivo*, we labeled the cortical neurons in transgenic GCaMP mice with AAV-induced ChR2 expression and used a 450-nm LED for wide-field, one-photon optogenetic stimulation. ChR2 was selected due to its lowest absorption at the PAM imaging wavelength among currently available opsins, therefore the smallest crosstalk in theory. As shown in Fig. R5a, when the PAM laser was off, the targeted neuron in the white box exhibited a clear response to LED optogenetic stimulation (applied during the first ~50 ms of TPM imaging, highlighted by the blue shaded time window). In contrast, when the PAM laser was on (Fig. R5b; pulse energy, 40 nJ; pulse repetition rate, 80 kHz) during image acquisition, the same neuron showed elevated baseline activity and no additional response to LED optogenetic stimulation. The time course of the GCaMP fluorescence intensity averaged over the neuronal ROI (Fig. R5c) further confirms

these observations. Notably, even after the PAM laser was turned off, the amplitude of the neuron's GCaMP response remained substantially reduced compared to the initial condition, implying possible photodamage to the ChR2 opsin by the PAM laser.

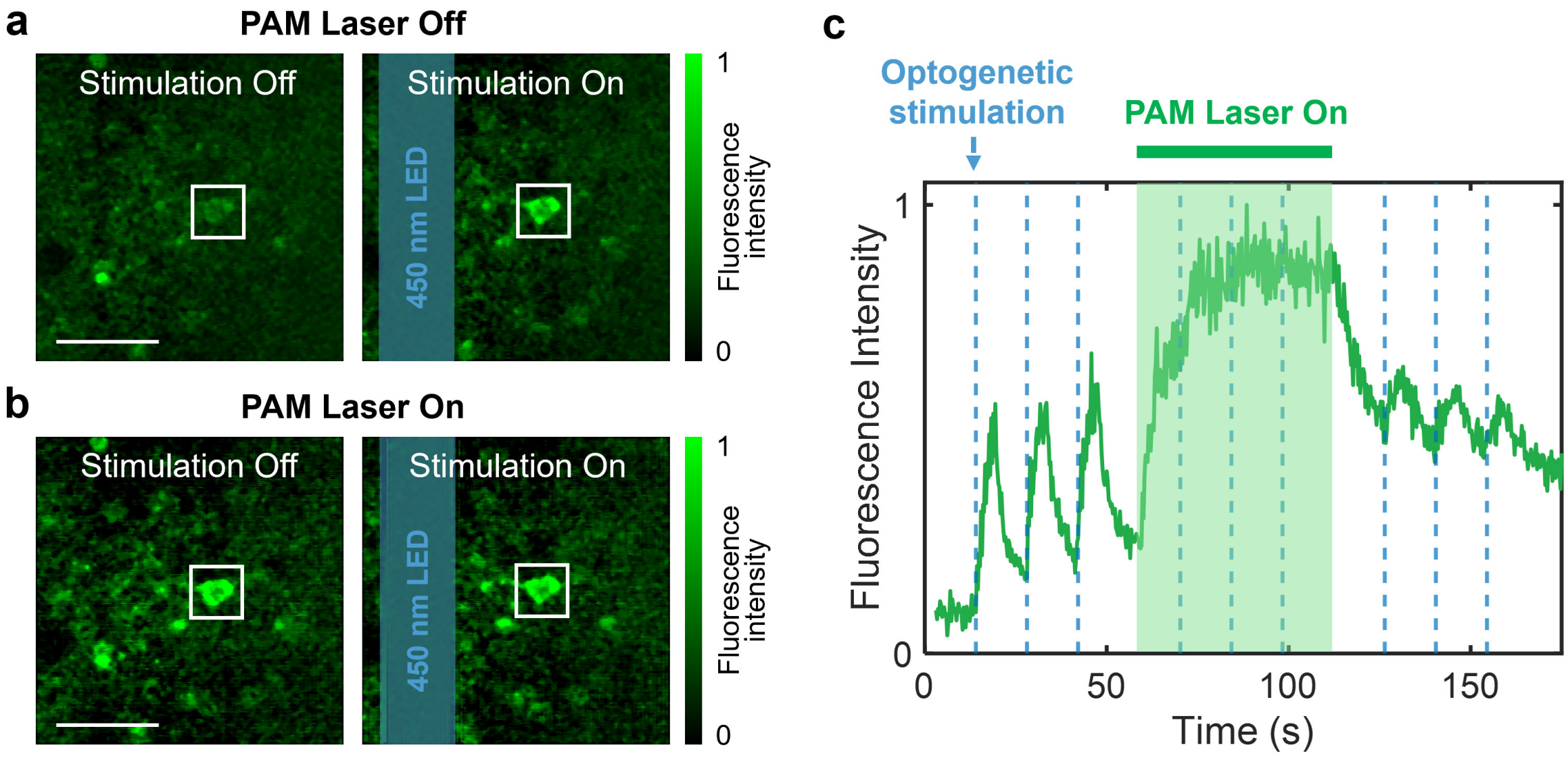

Fig. R5 | Crosstalk between PAM imaging and optogenetic manipulation. a, TPM images acquired before (left) and after (right) LED-based optogenetic stimulation with the PAM laser off. The LED stimulation was applied during the first ~50 ms of TPM imaging (blue shaded region). **b,** TPM images of the same field of view before (left) and after (right) LED stimulation with the PAM laser on. **c,** GCaMP response of the neuron in the white box over time. The blue lines indicate the LED stimulation (repeated 9 times). Green shading denotes the time window during which the PAM laser was turned on. Scale bar: 50 μm.

Pros of focused femtosecond laser stimulation: **(1) Label-free.** It does not require the expression of opsin in animal models, greatly simplifying animal preparation. **(2) Minimal crosstalk with imaging**. This type of stimulation requires tightly focusing high-power femtosecond laser (wavelength: 800 nm; average power: ~200 mW) on the neuronal membrane for a sustained duration (~1 s). So, the laser beams used for raster-scanning do not have sufficient power (PAM: wavelength, 545/558 nm; average power; 8 mW; TPM: wavelength, 930 nm; average power, 50 mW) and dwell time (12.5 μs) to induce neuronal activation. As a result, unintended stimulation is effectively avoided.

Cons of focused femtosecond laser stimulation: **Irreversibility.** This type of stimulation relies on the transient disruption of neuronal membrane induced by high-power femtosecond laser pulses, which may cause permanent cellular damage and is therefore irreversible. As a result, the stimulated neuron exhibits prolonged activation (>1 min) as observed both in cell culture[29] and *in vivo* (see calcium activity of N1 in Fig. 4b of the manuscript), which is in contrast to the transient dynamics of physiological spiking.

In summary, while the label-free femtosecond laser stimulation used in our study effectively avoids the crosstalk between imaging and manipulation, optogenetics is highly desirable for future studies because it offers reversible, cell-type-specific modulation. To this end, the development of new opsins with minimal spectral overlap with the green laser used for PAM imaging is needed. **We have provided a detailed discussion about the refinement of the optical manipulation approaches in the Discussion section (Lines 352–359).**

6. The authors mention optimizing the cladding materials of the MRR to enhance the ultrasound detection sensitivity of PAM. Specifically, they changed the top cladding from PDMS (refractive index ≈1.40) to a low-refractive-index optical acrylic resin (~1.31). Could the authors provide specific parameters of this optical acrylic resin and directly compare it with PDMS? Additionally, should the acoustic matching between the material and water also be considered, rather than focusing solely on improving the Q-factor? A more detailed discussion on this point would be helpful.

Response:

Thank you for this constructive suggestion.

The transition from PDMS to low-index optical acrylic resin was a deliberate design choice, motivated by the need to optimize both the ultrasound detection sensitivity and the acoustic impedance matching. A detailed analysis is provided below:

(1) <u>Ultrasound detection sensitivity</u>

MRR's ultrasound detection sensitivity is closely related to the Q-factor of the resonance supported by the whispering-gallery mode, which is governed by optical energy losses in the micro-ring waveguide. PDMS (Polydimethylsiloxane) is widely used as an elastomeric material, but it is not optimized for low-loss optical applications. As a result, **PDMS exhibits non-negligible optical absorption (extinction coefficient k ~10^{-6}), which can limit the achievable Q-factor of the MRR.** So, our primary interest is to find alternative soft optical materials with lower loss to replace PDMS as the cladding layer.

We investigated specially designed optical coating materials, including CYTOP and **MY-131-MC** as replacements for PDMS in the cladding layer. These materials exhibit low refractive indices near 1.3 and **significantly reduced optical loss (k < 10^{-7})**. Among them, MY-131-MC was selected based on cost considerations.

While precise material parameters are not available for rigorous quantitative comparison, our experimental observations confirm that **MRRs with low-index optical cladding offer an average Q-factor of 1.7×10^5, outperform those using PDMS cladding which have an average Q-factor of 3.4×10^4.**

Furthermore, the use of low-index cladding materials enables a tight confinement of the guided mode within the waveguide, reducing evanescent field leakage. This improved confinement makes the MRR sensor less susceptible to environmental perturbations and potential contaminations *in vivo*.

(2) Acoustic impedance mismatch

The large acoustic impedance mismatch in the case of PDMS is another reason that motivated us to search for alternative cladding materials. **Standard PDMS (e.g., Sylgard 184) has an acoustic impedance (Z) of ~1.1 MRayl**[30], which is in a clear contrast of water (Z ~1.5 MRayl[31]) and MRR made of polystyrene (Z ~2.5 MRayl[32]). Although the acoustic impedance of MY-131-MC is not explicitly reported by the manufacturer, it can be estimated based on its material properties. With a density of ~1.74 g/cm^3[33] and a Young's modulus of ~2 MPa[34], **MY-131-MC's acoustic impedance is expected to be between 1.7–2.3 MRayl**, assuming a longitudinal sound velocity of typical soft elastomer or fluorinated polymers (970–1310 m/s[35])**.**

Although PDMS is better matched to water acoustically, its significantly lower acoustic impedance leads to a pronounced mismatch with MRR's polystyrene waveguide, resulting in stronger internal reflection at the cladding-resonator interface. In contrast, MY-131-MC, whose acoustic impedance is closer to that of polystyrene, provides improved matching at this critical interface while maintaining a moderate mismatch with water. As a result, **MY-131-MC offers a more balanced impedance profile for the sandwiched configuration (water 1.5 MRayl → MY-131-MC 1.7–2.3 MRayl → polystyrene 2.5 MRayl),** reducing the internal reflection and improving overall acoustic transmission within the MRR structure.

We have updated both the main manuscript (Lines 103–104) and Supplementary Note 1 to provide a more detailed discussion.

7. The authors refer to anti-Stokes signals induced by the PAM nanosecond laser as a source of crosstalk in TPM imaging. Are these anti-Stokes signals generated by stimulated Raman scattering in the fiber? Can such signals not be removed by narrow-band optical filters used in the paper? In my understanding, could the crosstalk actually arise from back-reflected Stokes light from the sample? Could the authors provide additional experimental data to support their conclusion and further clarify this mechanism?

Response:

Thank you for this insightful comment.

Based on our new data (Fig. R6), the crosstalk signals likely arise from two primary sources:

Stray light of the PAM 545/558 nm laser. When the PAM laser was off, the PMT signal only consisted of random noise (Fig. R6a). When the PAM laser was turned on, a weak crosstalk signal with a fixed interval of 12.5 μs that matches the pulse repetition rate of the PAM laser (80 kHz) appeared in the PMT signal, even in the absence of any sample beneath the objective (Fig. R6b). In our system, stimulated Raman scattering in the optical fiber is used to shift the 532 nm nanosecond laser light to 545 and 558 nm for PAM imaging. However, the output light from the fiber is filtered using narrow-band bandpass filters (546/10 and 560/10), which do not have a spectral overlap with the fluorescence filter (503/40) used in the TPM channel. Therefore, direction transmission of the PAM laser beam to the TPM channel is highly unlikely. Instead, the crosstalk observed in Fig. R6b should be attributed to the stray PAM excitation light, such as backscattered light at the air-lens interfaces of the microscope objective. Due to the non-normal incidence angle, this stray light may partially bypass the rejection band of the PMT filter (503/40), resulting in crosstalk. When imaging the brain of a wild-type C57 mouse (~200 μm below the dura), the amplitude of the crosstalk increases (Fig. R6c) due to the enhanced backscattering of PAM laser light by the tissue.

Anti-Stokes fluorescence in the GCaMP mouse. Anti-Stokes fluorescence emission is a single-photon excitation process of a fluorophore at the long-wavelength tail of the excitation spectrum, so that the fluorescence emission actually occurs at a wavelength shorter than the excitation[36]. Previous studies have shown that green fluorescent protein can be excited at 532 nm and emit fluorescence peaking near 509 nm[36]. Consistent with this phenomenon, we observed that the crosstalk signal in a GCaMP mouse (Fig. R6d) was approximately twofold stronger than the scattering-induced crosstalk in a wild-type C57 mouse (Fig. R6c), under the same experimental conditions (~200 μm depth; 100 nJ pulse energy). This result supports the presence of anti-Stokes fluorescence from GCaMP as an additional source of crosstalk in TPM imaging.

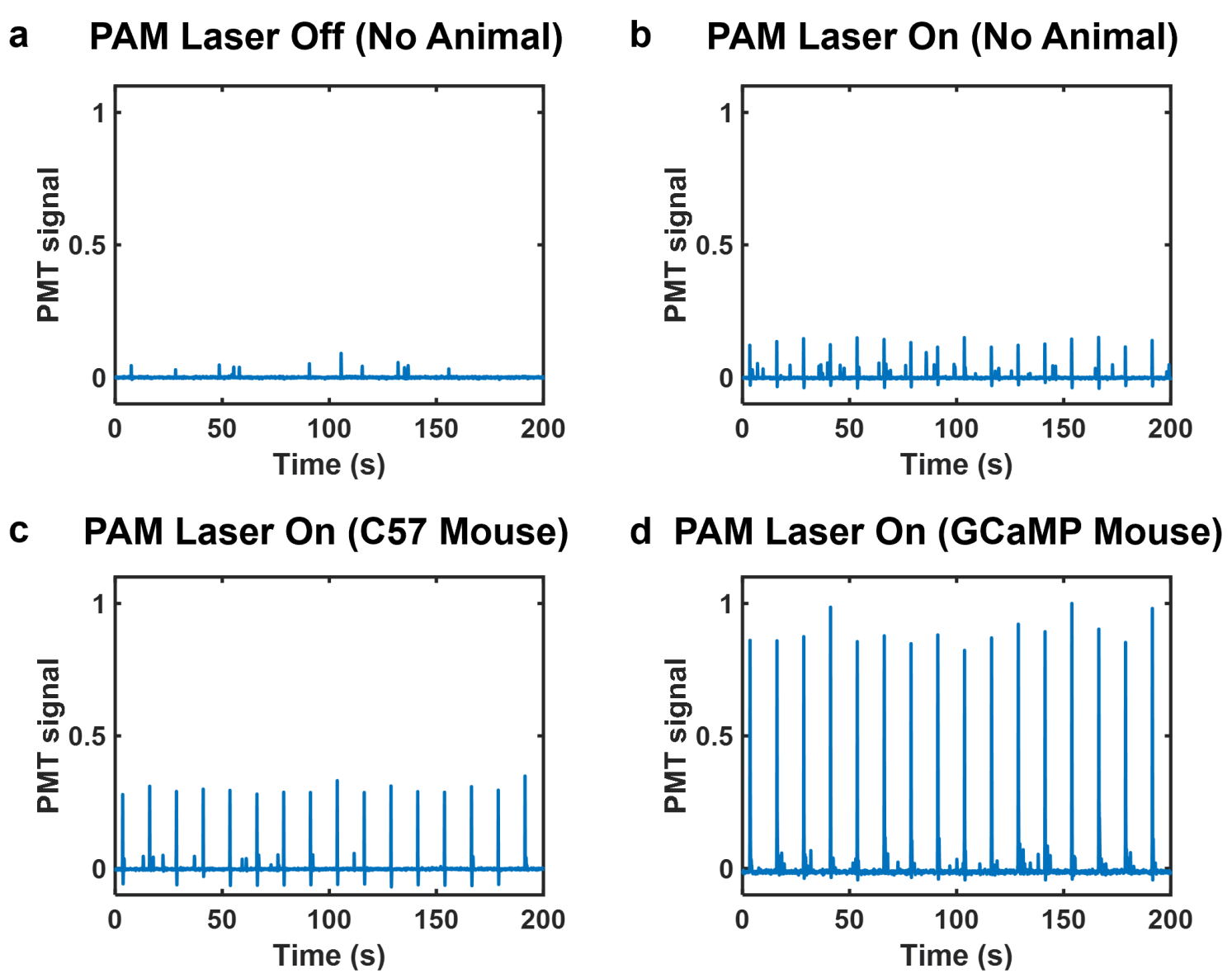

Fig. R6 | Crosstalk of the PAM laser in TPM detection channel. a, PMT signal with the PAM laser turned off. **b,** PMT signal with the PAM laser turned on, acquired without any sample beneath the microscope objective. **c,** PMT signal with the PAM laser turned on, acquired while scanning a wild-type C57 mouse brain. **d,** PMT signal with the PAM laser turned on, acquired while scanning a transgenic GCaMP mouse brain. Identical experimental conditions and PAM laser parameters (80 kHz, 100 nJ) were maintained across all measurements.

We have updated the Methods section in the manuscript to clarify the primary sources of the crosstalk (Lines 702–708).

Minor comments:

1.The colors of the upper part of Fig.3c and Fig.4c are too pale to see clearly. Please revise them.

Response:

Thank you for this suggestion. **We have updated the figures to address this issue.**

2.It is recommended to provide detailed imaging parameters such as the pixel numbers, pulse repetition rate and pulse energy for the in vivo imaging experiment in the Methods section or the supplementary file.

Response:

Thank you for this suggestion. **We have added the following description to the Methods section (Lines 504–510):**

For all experiments in this work, the PAM nanosecond laser operated at a repetition rate of 80 kHz with a pulse energy of 100 nJ. The TPM femtosecond laser operated at 80 MHz with an average power of 40 mW. The FOV for all dynamic imaging was set to 150 × 150 or 200 × 200 μm^2 with a fixed pixel number of 150 × 150, corresponding to an imaging frame rate of 3.56 Hz and an effective imaging frame rate of 2.54 Hz when considering kymographic scanning. A detailed summary of the key parameters for each experiment is provided in Table R3.

3.What are the depths of field of TPM and PAM in the imaging system? What are the depths of the neurons and blood vessels, respectively?

Response:

The depth of field of TPM is equal to the axial resolution, which was estimated to be 2.4 μm

in Extended Data Fig. 4f. The depth of field of PAM, determined by the Rayleigh range of the focused Gaussian beam, was calculated to ~64 μm with a numerical aperture of 0.1 at 545 nm.

The maximum imaging depth of PAM is limited to ~300 μm due to the strong absorption and scattering of green excitation light in the brain tissue. Since the focal planes of TPM and PAM are axially co-aligned, the imaged neurons and blood vessels should all located within a depth range of approximately 100–300 μm below the dura (cortical layers II/III) in all experiments presented in this work.

We have included information about the imaging depth of neurons and blood vessels in the section of imaging parameters in the Methods (Lines 510–512).

4.In the experiment of single-capillary occlusion, supplementary video 3 shows that an abnormal fluorescence signal appeared at 83.2 s (marked by the red rectangle in the following image). How to explain this interesting phenomenon? It is quite similar to the imaging result of single-neuron stimulation shown in supplementary video 4. Does the 800-nm fs laser beam used to occlude the blood vessel have an impact on the nearby neurons? Or is it the physiological response of the neurons by the moment when the blood vessel (marked by P1) is occluded?

Response:

Thank you for this insightful comment.

We think that the observed GCaMP signal spreading from the occlusion site represents a physiological neuronal response to capillary occlusion, based on following considerations:

(1) Highly confined nonlinear interaction volume

Capillary occlusion is induced by the nonlinear absorption of high-energy ultrashort pulses at the focal point, resulting in ionization within a highly confined femtoliter-scale volume[37]. In our system, the two-photon absorption volume is ~0.7 μm laterally and ~2.4 μm axially, substantially smaller than the ~10 μm capillary diameter. Therefore, the manipulation is tightly confined within the lumen of the targeted capillary, ensuring minimal collateral damage to the surrounding extravascular tissue and neurons.

(2) Distinct spatiotemporal characteristics

The fluorescence signals observed in response to single-capillary occlusion differ from those in the single-neuron stimulation experiment. In the neuronal stimulation study, activation of the targeted neuron propagates through the synaptic network and leads to transient (<5 s), spatially confined calcium signals which are primarily localized to neuronal somata. In contrast, the capillary occlusion induces a radially spreading

fluorescence signal from the occlusion site, extending to both somata and dendrites, while exhibiting a more prolonged temporal profile (>1 min). These differences suggest distinct underlying physiological mechanisms.

(3) Astrocyte-mediated signaling within the neurovascular unit

We hypothesize that astrocytes, which ensheathe capillaries and serve as intermediaries between endothelial cells and neurons[38], may play a key role in this phenomenon. Specifically, during capillary occlusion, the laser-induced ionization can generate cavitation bubbles and shock waves, imposing mechanical stress on endothelial cells[37]. This perturbation may activate astrocytes ensheathing the capillary wall, which subsequently propagate intracellular signals to nearby neurons and lead to the observed fluorescence response.

5. Please ensure the numerical labeling (e.g., DM1, DM2, …) for “DM” is consistent between Fig. 1 and Extended Fig. 3 to improve readability.

Response:

Thank you for this suggestion. **We have updated the labeling in Extended Fig. 3 to ensure consistency.**

Reviewer #2 (Remarks to the Author):

This manuscript presents a comprehensive study of neurometabolic coupling (NMC) at the level of single microvessels and individual neurons, enabled by an innovative dual-modal imaging platform that unified two-photon fluorescence microscopy (for GCaMP-based neural activity imaging) with photoacoustic imaging for quantitative hemodynamic assessment. A key technical innovation is the development of a thin, optically transparent, and acoustically sensitive micro-ring resonator (MRR) ultrasound detector, which permits the passage of a tightly focused excitation beam for two-photon imaging (as well as beams for single-capillary occlusion and single-neuron stimulation).

In vivo quantitative imaging demonstrates the feasibility of this unified platform for assessing multiple NMC-related parameters, including arteriole diameter, blood flow velocity, volumetric flow rate, oxygen saturation, oxygen flux, oxygen release rate, and oxygen extraction fraction. The study design is scientifically sound, the results are comprehensive, and the conclusions are well supported by appropriate analyses. Overall, this platform is highly valuable and has strong potential to enable fundamental investigations of neurological diseases.

While this is an excellent manuscript, a few points would benefit from clarification prior to publication. Most of the concerns are minor:

Response:

Thank you for the positive comments and constructive suggestions. Our detailed responses to the specific comments are provided below.

1. Sample size and experimental design.

Please specify the number of mice used for each type of experiment. Were experiments repeated across different days? If so, how many sessions were performed per mouse on each day? These details are important for determining the appropriate statistical model.

Response:

Thank you for the constructive suggestion.

Below please find a summary of the number of animals and trials used for each type of experiment:

- ***Whisker stimulation***: We performed imaging and 10 trials of stimulation on 1 mouse within the same day. The results in Fig. 2 are derived from the responses of 9 neurons and 8 vessels across all 10 trials.
- ***Single-capillary occlusion***: We performed imaging and 1 trial of manipulation on 1

mouse. The results in Fig. 3 are derived from the responses of 40 neurons and 7 capillaries.

- ***Single-neuron stimulation***: We performed imaging and 1 trial of manipulation on 1 mouse. The results in Fig. 4 are derived from the responses of 24 neurons and 5 capillaries.

We wish to take this opportunity to clarify that the primary scope of this work is to demonstrate the technical capability of the TPM-PAM system for neurovascular imaging and manipulation at the single-cell level, rather than conducting a hypothesis-driven biological study that requires large-scale population statistics. Thus, large sample size and cross-animal statistical modeling were not applied. Instead, our experimental design and analysis focus on demonstrating robust signal extraction and technical reliability at the single-event level.

To further demonstrate the technical repeatability and robustness of the TPM-PAM system, we conducted additional experiments across all three types of neurovascular manipulation:

- ***Whisker stimulation (Fig. R7)***: We performed imaging over 5 trials of stimulation on 1 mouse within the same day. Representative snapshots acquired immediately before and after stimulation in the first and second trials are shown in Fig. R7. In both trials, clear vasodilation in arterioles and increased sO_2 in both venules and capillaries are observed, consistent with the results presented in Fig. 2 of the main manuscript.
- ***Single-neuron stimulation (Fig. R3) and single-capillary occlusion (Fig. R4)***: For a detailed discussion of these new results, please refer to our response to Reviewer #1, Comment 4, Point (3).

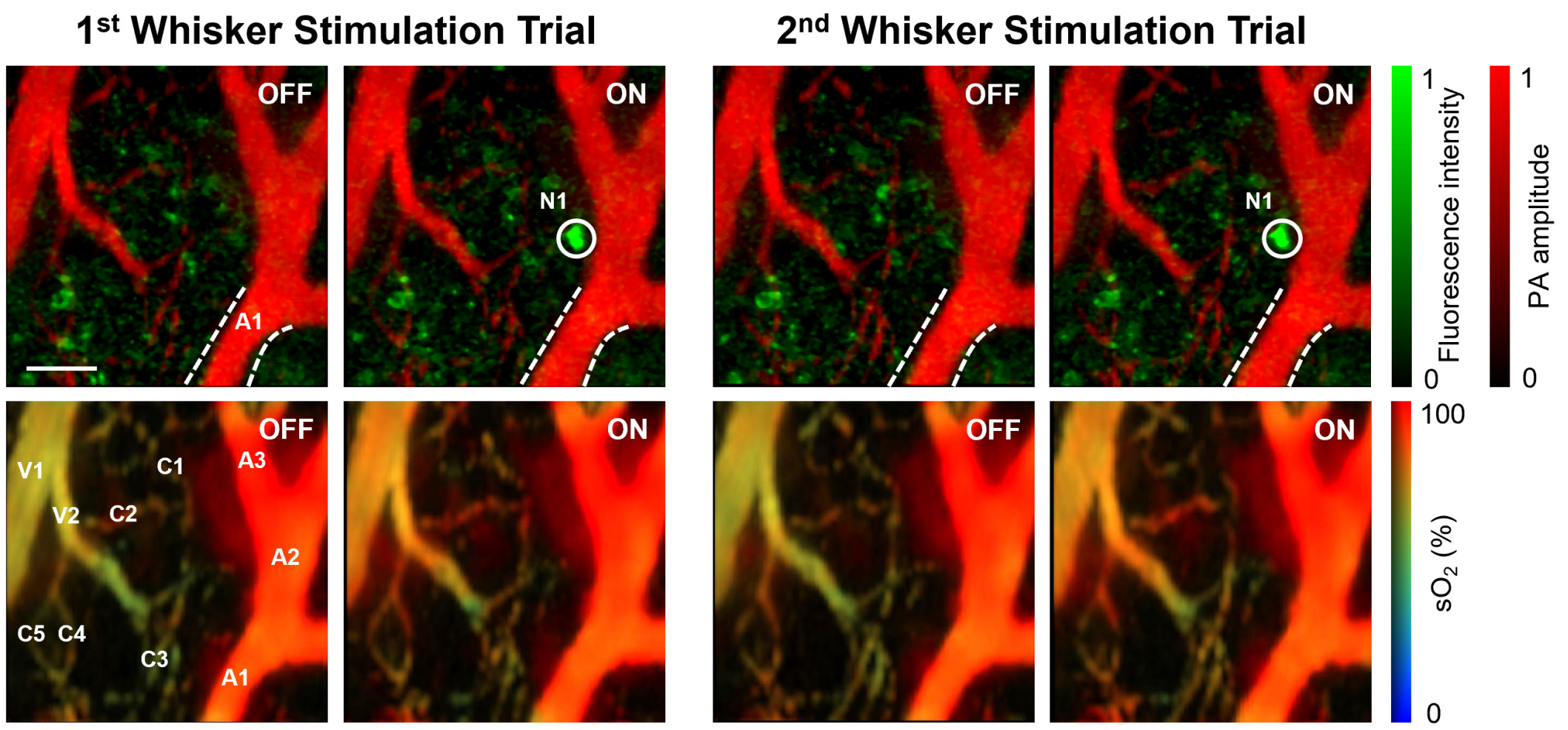

Fig. R7 | TPM-PAM snapshots of whisker stimulation across two trials in the awake GCaMP mouse brain. Top, overlay of neuronal activity and microvascular structure. The dotted outline marks the boundary of arteriole A1 after stimulation, clearly showing

vasodilation. Bottom, corresponding sO_2 maps. Scale bar: 50 μm.

We have included detailed information about the number of mice used for each type of experiment in the section of imaging parameters in Methods (Lines 513–518).

2. Imaging depth and cortical layer.
Which cortical layer(s) of the somatosensory cortex were imaged? Please provide the approximate imaging depth below the dura.

Response:

Thank you for this constructive suggestion.

Across all experiments, the imaging depth was approximately 100–300 μm below the dura, corresponding to cortical layers II/III. This imaging depth is mainly limited by the green excitation laser used for PAM imaging, which suffers from strong tissue absorption and scattering.

We have clarified the imaging depth and cortical layer in the section of imaging parameters in the Methods (Lines 510–512).

3. Field-of-view (FOV) selection.
Please briefly describe the criteria used to select imaging FOVs. If FOVs were selected randomly, please state it explicitly in the manuscript.

Response:

Thank you for this constructive suggestion.

For whisker stimulation, imaging was performed in the primary somatosensory barrel cortex. To identify an optimal FOV, we manually surveyed the cortical tissue using a 3D translation stage during real-time imaging and repeated whisker stimulation. The final FOV was selected based on the following criteria:

- at least one neuron within the FOV exhibited robust activation in response to stimulation;
- the FOV contained all major vessel types (i.e., arterioles, venules, and capillaries) to enable comparative analysis of their distinct responses.

For single-capillary occlusion and single-neuron stimulation, the FOV was selected based on the following criterion:

- the vascular network within the FOV was predominantly composed of capillaries rather than large vessels, as the primary focus here was to analyze oxygen release from capillaries to neurons.

We have clarified the FOV selection criteria in the Methods (Lines 520–531).

4. Data presentation.
Where appropriate, consider presenting quantitative results in the form of (mean ± SD).

Response:

Thank you for this constructive suggestion.

We have updated the quantitative results for the whisker stimulation experiments in the form of (mean ± SD) in the manuscript (Lines 196–206; Line 307):

- Arterioles: D (14% ± 2%); CBF (35% ± 7%); fO_2 (39% ± 7%);
- Venules: V_f (38% ± 8%); D (4% ± 1%); CBF (58% ± 11%); sO_2 (6% ± 1%); fO_2 (70% ± 14%);
- Capillaries: V_f (67% ± 24%); C_{Hb} (9% ± 4%); sO_2 (10% ± 2%); rO_2 (113% ± 48%);
- OEF (10% ± 2%).

5. Line 95–96: "four-fold improvement in coupling."
Please clarify what "coupling" refers to (e.g., for the single-model light in and out of the MRR for acoustic signal detection?). Additionally, what specific design features contributed to this improvement—MRR material properties, thickness, geometric dimensions, or fabrication precision (e.g., ring dimensional control)?

Response:

Thank you for this insightful comment.

In this work, "coupling" refers to the ratio of the optical power at the output multimode fiber to that at the input single-mode fiber, accounting for coupling losses at both the input and output ports of the MRR waveguide. Because the coupling loss from the MRR waveguide to the multimode fiber (with a large collection numerical aperture of 0.4) is significantly smaller than that from the single-mode fiber to the MRR waveguide, the overall coupling loss is predominantly from the input port due to mode mismatch. Accordingly, the coupling improvement in this work is primarily focused on reducing the input coupling loss.

The coupling improvement at the input port was achieved by the geometric design of adiabatically tapered waveguide for mode conversion. Specifically, in our previous MRR design, the single-mode fiber (SMF) with a core diameter of 3.5 μm was directly coupled to a straight bus waveguide (straight WG) with a cross-section of 850 nm × 850 nm. The large mode mismatch between the SMF and straight WG resulted in a low coupling efficiency of < 5%. To mitigate this issue, we introduced adiabatically tapered waveguides (Tapered WGs)

for mode conversion. The tapered WG was 3-μm wide at the entrance, matching the size of the SMF core, and gradually narrowed to 850 nm to match the straight WG. This design effectively increased the coupling efficiency from 5% to 19%, corresponding to a four-fold improvement.

We have updated both the main manuscript (Lines 105–107) and Supplementary Note 1 to provide a clearer description.

6. Line 137: axial resolution.
The reported axial resolution of 2.4 μm appears unusually high for the stated 0.7 μm lateral resolution. Please clarify the method used to characterize axial resolution, as the value may depend on the measurement approach or robustness.

Response:

Thank you for this constructive comment.

The method used to characterize the axial resolution is clarified as follows: (1) TPM imaging was performed on a 0.5-μm-diameter fluorescent bead. A motorized translation stage was used to scan the bead along the axial direction, enabling the acquisition of a 3D image stack. (2) The axial intensity profile at the center of the bead was extracted (Extended Data Fig. 4f), based on which the axial resolution of TPM was quantified to be 2.4 μm by extracting the full width at half maximum (FWHM) value.

According to previous studies, the optical diffraction-limited lateral and axial resolutions of TPM can be estimated using the following formulas[39]:

$$d_{xy} = \begin{cases} \dfrac{0.64\lambda}{\sqrt{2}NA} & NA \le 0.7 \\ \dfrac{0.65\lambda}{\sqrt{2}NA^{0.91}} & NA > 0.7 \end{cases}, \quad d_z = \frac{1.064\lambda}{\sqrt{2}} \times \frac{1}{\left(n - \sqrt{n^2 - NA^2}\right)},$$

where d_{xy} and d_z are the 1/e diameter for the square of the illumination point spread function ($IPSF^2$), λ is wavelength, NA is numerical aperture, n is the refractive index of the immersion medium. According to the above formulas, for our TPM system (λ = 930 nm, NA = 1.0, n = 1.326), d_{xy} and d_z are calculated to be 0.43 μm and 1.54 μm, respectively, indicating that the theoretical axial resolution is ~3.6-fold poorer than the lateral resolution.

Both the experimentally measured lateral and axial resolutions of our TPM system are lower than the optical diffraction-limited values due to practical aberrations in the system. Despite this, **the measured axial resolution (2.4 μm) is 3.4-fold poorer than the lateral resolution (0.7 μm), which is in good agreement with the theoretical estimation.**

7. Line 147: Radon transform.

Please briefly explain what the Radon transform was used for in kymography-based flow speed measurements, which can be valuable to for readers who may be less familiar with this approach.

Response:

Thank you for this constructive suggestion.

The kymograph acquired in this work represents the spatiotemporal trajectories of RBCs, with the horizontal axis corresponding to spatial position (x-axis) and the vertical axis representing time (t-axis). As RBCs move, they create distinct diagonal streaks across the kymograph. Therefore, the orientation angles of these motion streaks in the kymograph directly encode the RBC flow speed. The Radon transform is a mathematical tool applied to extract the dominant orientation angle objectively and accurately[40].

As shown in Extended Data Fig. 5, the Radon transform is applied to project the kymograph over a range of angles, and the variance is then calculated for each projection. The angle corresponding to the maximum variance, denoted as θ_{peak}, represents the dominant streak orientation. Finally, the flow speed is calculated based on the orientation angle by $V_f = \frac{\Delta x}{\Delta t \times \tan(\theta_{\mathrm{peak}})}$, where Δx and Δt are the pixel size along the x and t axes, respectively.

We have updated the section of data analysis in the Methods to provide a clearer description (Lines 626–637).

8. Lines 184–189: quantitative changes.

The manuscript reports a 35% increase in CBF and a 70% rise in fO_2. Were these values directly measured, or analytically derived from other measured parameters? Please clarify.

Response:

Thank you for this important comment.

In whisker stimulation, the temporal dynamics of a total of 8 oxygen-metabolic parameters are quantified (Fig. 2d). Among these, vessel diameter (D), flow speed (V_f), oxygen saturation (sO_2), hemoglobin concentration (C_{Hb}), and oxygen release rate (rO_2) are directly measured, while **volumetric blood flow (CBF), oxygen flux rate (fO_2), and oxygen extraction fraction (OEF) are derived from those direct measurements.**

Specifically, the CBF of each vessel was calculated from the corresponding values of D and V_f at each time point (see the data analysis section in Methods for detailed formulas). The temporal response of CBF was then obtained for each vessel, and the averaged result

across vessels of the same type and across 10 stimulation trials was shown in Fig. 2d (first row, third panel), demonstrating a ~35% peak increase in arterioles (red curve).

Similarly, fO_2 was computed based on CBF, C_{Hb}, and sO_2 (see the data analysis section in Methods for detailed formulas). Its temporal dynamics was presented in Fig. 2d (second row, second panel), showing a ~70% peak increase in venules (purple curve).

We have updated the section of data analysis in the Methods to provide a clearer clarification (Lines 664–667).

9. Lines 197–204: statistical analysis.
Given the expected inter-animal variability and potentially limited sample size, the assumption of Gaussian distribution may not hold and thus it is unclear if t-test would be suitable for statistical analysis.

Response:

Thank you for this insightful comment. We fully agree that the assumption of normal distribution should be verified prior to applying a *t*-test.

First, regarding inter-animal variability, we wish to clarify (as detailed in our response to Comment #1) that, for each of the three manipulation experiments, the statistical analysis was performed using data acquired from a single animal. Therefore, inter-animal variability does not confound these intra-animal comparisons.

Second, to ensure the validity of our chosen statistical model, we assessed the normality of individual data distributions before performing the *t*-tests shown in Fig. 2f-g. Specifically, the normality was assessed using the Lilliefors test (*lillietest* function in MATLAB), which provides a test decision for the null hypothesis that the data comes from a distribution in the normal family against the alternative that it does not come from such a distribution. In all cases, the test returned a value of 0, indicating that the null hypothesis cannot be rejected at the 5% significance level. These results support the assumption of a normal distribution and the validity of applying the *t*-test in our analysis.

We have included information about the normality test in the Methods (Lines 693–701).

References

1. Fang, C., Hu, H. & Zou, J. A Focused Optically Transparent PVDF Transducer for Photoacoustic Microscopy. *IEEE Sensors Journal* **20**, 2313–2319 (2020).
2. Mirg, S. *et al.* Awake mouse brain photoacoustic and optical imaging through a transparent ultrasound cranial window. *Opt. Lett., OL* **47**, 1121–1124 (2022).
3. Chen, H. *et al.* A High Sensitivity Transparent Ultrasound Transducer based on PMN-PT for Ultrasound and Photoacoustic Imaging. *IEEE Sens Lett* **5**, 2500804 (2021).
4. Qiu, C. *et al.* Transparent ultrasonic transducers based on relaxor ferroelectric crystals for advanced photoacoustic imaging. *Nat Commun* **15**, 10580 (2024).
5. Ren, D., Sun, Y., Shi, J. & Chen, R. A Review of Transparent Sensors for Photoacoustic Imaging Applications. *Photonics* **8**, (2021).
6. Li, H. *et al.* Disposable ultrasound-sensing chronic cranial window by soft nanoimprinting lithography. *Nat Commun* **10**, 4277 (2019).
7. Li, H., Dong, B., Zhang, Z., Zhang, H. F. & Sun, C. A transparent broadband ultrasonic detector based on an optical micro-ring resonator for photoacoustic microscopy. *Sci Rep* **4**, 4496 (2014).
8. Guggenheim, J. A. *et al.* Ultrasensitive plano-concave optical microresonators for ultrasound sensing. *Nature Photon* **11**, 714–719 (2017).
9. Zhong, X. *et al.* Free-moving-state microscopic imaging of cerebral oxygenation and hemodynamics with a photoacoustic fiberscope. *Light Sci Appl* **13**, 5 (2024).
10. Liang, Y. *et al.* Optical-resolution functional gastrointestinal photoacoustic endoscopy based on optical heterodyne detection of ultrasound. *Nat Commun* **13**, 7604 (2022).
11. Huynh, N. T. *et al.* A fast all-optical 3D photoacoustic scanner for clinical vascular imaging. *Nat. Biomed. Eng* **9**, 638–655 (2025).
12. Ansari, R., Zhang, E. Z., Desjardins, A. E. & Beard, P. C. All-optical forward-viewing photoacoustic probe for high-resolution 3D endoscopy. *Light Sci Appl* **7**, 75 (2018).
13. Optical ultrasound sensors for photoacoustic imaging: a review. https://www.spiedigitallibrary.org/journals/journal-of-biomedical-optics/volume-29/issue-S1/S11523/Optical-ultrasound-sensors-for-photoacoustic-imaging-a-review/10.1117/1.JBO.29.S1.S11523.full.
14. Zhou, J., Wang, W., Jing, L. & Chen, S.-L. Dual-modal imaging with non-contact photoacoustic microscopy and fluorescence microscopy. *Opt. Lett., OL* **46**, 997–1000 (2021).
15. Hajireza, P., Shi, W., Bell, K., Paproski, R. J. & Zemp, R. J. Non-interferometric photoacoustic remote sensing microscopy. *Light Sci Appl* **6**, e16278–e16278 (2017).
16. Yao, J. When pressure meets light: detecting the photoacoustic effect at the origin. *Light Sci Appl* **6**, e17062–e17062 (2017).

17. Reza, P. H., Bell, K., Shi, W., Shapiro, J. & Zemp, R. J. Deep non-contact photoacoustic initial pressure imaging. *Optica, OPTICA* **5**, 814–820 (2018).
18. Bell, K. L., Reza, P. H. & Zemp, R. J. Real-time functional photoacoustic remote sensing microscopy. *Opt. Lett., OL* **44**, 3466–3469 (2019).
19. Haven, N. J. M., Martell, M. T., Li, H., Hogan, J. D. & Zemp, R. J. Investigating mechanisms of laser pulse-induced reflectivity modulations in photoacoustic remote sensing with a 10 million frames-per-second camera. *Sci Rep* **13**, 3751 (2023).
20. Hillman, E. M. C. Coupling Mechanism and Significance of the BOLD Signal: A Status Report. *Annu Rev Neurosci* **37**, 161–181 (2014).
21. Buxton, R. B., Griffeth, V. E. M., Simon, A. B. & Moradi, F. Variability of the coupling of blood flow and oxygen metabolism responses in the brain: a problem for interpreting BOLD studies but potentially a new window on the underlying neural activity. *Front. Neurosci.* **8**, (2014).
22. Huneau, C., Benali, H. & Chabriat, H. Investigating Human Neurovascular Coupling Using Functional Neuroimaging: A Critical Review of Dynamic Models. *Front Neurosci* **9**, 467 (2015).
23. Wilson, D. F. & Matschinsky, F. M. Cerebrovascular Blood Flow Design and Regulation; Vulnerability in Aging Brain. *Front. Physiol.* **11**, (2020).
24. Zhu, L. *et al.* Single-microvessel occlusion produces lamina-specific microvascular flow vasodynamics and signs of neurodegenerative change. *Cell Reports* **42**, 112469 (2023).
25. Shi, L. *et al.* A new era for stroke therapy: Integrating neurovascular protection with optimal reperfusion. *J Cereb Blood Flow Metab* **38**, 2073–2091 (2018).
26. Krogh, A. The supply of oxygen to the tissues and the regulation of the capillary circulation. *J Physiol* **52**, 457–474 (1919).
27. Devor, A. *et al.* 'Overshoot' of O_2 is required to maintain baseline tissue oxygenation at locations distal to blood vessels. *J Neurosci* **31**, 13676–13681 (2011).
28. Yang, W., Carrillo-Reid, L., Bando, Y., Peterka, D. S. & Yuste, R. Simultaneous two-photon imaging and two-photon optogenetics of cortical circuits in three dimensions. *eLife* **7**, e32671 (2018).
29. Hosokawa, C., Sakamoto, Y., Kudoh, S. N., Hosokawa, Y. & Taguchi, T. Femtosecond laser-induced stimulation of a single neuron in a neuronal network. *Appl. Phys. A* **110**, 607–612 (2013).
30. Xu, G. *et al.* Acoustic Characterization of Polydimethylsiloxane for Microscale Acoustofluidics. *Phys. Rev. Appl.* **13**, 054069 (2020).
31. Lin, D.-S., Zhuang, X., Wong, S. H., Kupnik, M. & Khuri-Yakub, B. T. Encapsulation of Capacitive Micromachined Ultrasonic Transducers Using Viscoelastic Polymer. *Journal of Microelectromechanical Systems* **19**, 1341–1351 (2010).
32. Xie, Y. *et al.* Acoustic Cell Separation Based on Density and Mechanical Properties. *J*

Biomech Eng **142**, 0310051–0310059 (2020).
33. MY-131-MC: Optical Coating Material. *MY Polymers* https://www.mypolymers.com/p/2255/.
34. MY-130: Optical Coating / Adhesive. *MY Polymers* https://www.mypolymers.com/p/2268/.
35. Rae, P. j. & Brown, E. n. Some Observations on Measuring Sound Speeds in Polymers Using Time-of-Flight. *Experimental Techniques* **n/a**,.
36. Chiu, L. *et al.* Protein expression guided chemical profiling of living cells by the simultaneous observation of Raman scattering and anti-Stokes fluorescence emission. *Sci Rep* **7**, 43569 (2017).
37. Nishimura, N. *et al.* Targeted insult to subsurface cortical blood vessels using ultrashort laser pulses: three models of stroke. *Nat Methods* **3**, 99–108 (2006).
38. Astrocyte-neuron metabolic cooperation shapes brain activity: Cell Metabolism. https://www.cell.com/cell-metabolism/fulltext/S1550-4131(21)00322-3.
39. Young, M. D., Field, J. J., Sheetz, K. E., Bartels, R. A. & Squier, J. A pragmatic guide to multiphoton microscope design. *Adv. Opt. Photon.* **7**, 276 (2015).
40. Drew, P. J., Blinder, P., Cauwenberghs, G., Shih, A. Y. & Kleinfeld, D. Rapid determination of particle velocity from space-time images using the Radon transform. *J Comput Neurosci* **29**, 5–11 (2010).
